# Whole-genome, transcriptome, and methylome analyses provide insights into the evolution of platycoside biosynthesis in *Platycodon grandiflorus*, a medicinal plant

**DOI:** 10.1038/s41438-020-0329-x

**Published:** 2020-07-01

**Authors:** Jungeun Kim, Sang-Ho Kang, Sin-Gi Park, Tae-Jin Yang, Yi Lee, Ok Tae Kim, Oksung Chung, Jungho Lee, Jae-Pil Choi, Soo-Jin Kwon, Keunpyo Lee, Byoung-Ohg Ahn, Dong Jin Lee, Seung-il Yoo, In-Gang Shin, Yurry Um, Dae Young Lee, Geum-Soog Kim, Chang Pyo Hong, Jong Bhak, Chang-Kug Kim

**Affiliations:** 1 Personal Genomics Institute, Genome Research Foundation, Osong, 28160 Korea; 2 Genomics Division, National Institute of Agricultural Sciences (NAS), Jeonju, 54874 Korea; 3 Theragen Etex Bio Institute, Suwon, 16229 Korea; 4 Department of Plant Science, Plant Genomics and Breeding Institute, and Research Institute of Agriculture and Life Sciences, College of Agriculture and Life Sciences, Seoul National University, Seoul, 08826 Korea; 5 Department of Industrial Plant Science & Technology, Chungbuk National University, Cheongju, 28644 Korea; 6 Department of Herbal Crop Research, National Institute of Horticultural and Herbal Science, Rural Development Administration (RDA), Eumseong, 27709 Korea; 7 Clinomics Inc, Ulsan, 44919 Korea; 8 Green Plant Institute, Yongin, 16954 Korea; 9 Korean Genomics Center (KOGIC), Ulsan National Institute of Science and Technology (UNIST), Ulsan, 44919 Korea; 10 Department of Biomedical Engineering, School of Life Sciences, Ulsan National Institute of Science and Technology (UNIST), Ulsan, 44919 Korea

**Keywords:** Plant molecular biology, Secondary metabolism

## Abstract

Triterpenoid saponins (TSs) are common plant defense phytochemicals with potential pharmaceutical properties. *Platycodon grandiflorus* (Campanulaceae) has been traditionally used to treat bronchitis and asthma in East Asia. The oleanane-type TSs, platycosides, are a major component of the *P. grandiflorus* root extract. Recent studies show that platycosides exhibit anti-inflammatory, antiobesity, anticancer, antiviral, and antiallergy properties. However, the evolutionary history of platycoside biosynthesis genes remains unknown. In this study, we sequenced the genome of *P. grandiflorus* and investigated the genes involved in platycoside biosynthesis. The draft genome of *P. grandiflorus* is 680.1 Mb long and contains 40,017 protein-coding genes. Genomic analysis revealed that the *CYP716* family genes play a major role in platycoside oxidation. The *CYP716* gene family of *P. grandiflorus* was much larger than that of other Asterid species. Orthologous gene annotation also revealed the expansion of *β-amyrin synthases* (*bASs*) in *P. grandiflorus*, which was confirmed by tissue-specific gene expression. In these expanded gene families, we identified key genes showing preferential expression in roots and association with platycoside biosynthesis. In addition, whole-genome bisulfite sequencing showed that *CYP716* and *bAS* genes are hypomethylated in *P. grandiflorus*, suggesting that epigenetic modification of these two gene families affects platycoside biosynthesis. Thus whole-genome, transcriptome, and methylome data of *P. grandiflorus* provide novel insights into the regulation of platycoside biosynthesis by *CYP716* and *bAS* gene families.

## Introduction

Triterpenoid saponins (TSs) are naturally occurring, amphipathic, structurally, and functionally diverse phytochemicals consisting of triterpenoid or steroidal aglycones linked to oligosaccharide moieties^1^. TSs play important ecological functions, contribute to pest and pathogen resistance in plants, regulate crop quality, and are used for a wide range of applications in the pharmaceutical, pesticide, cosmetic, and food sectors^1,2^. Previous studies indicate that TSs exhibit diverse number of therapeutic properties, including immunostimulatory, hypocholesterolemic, hepatoprotective, anticarcinogenic, neuroprotective, anti-inflammatory, antiviral, antiprotozoan, molluscicidal, and antioxidative properties^1,3^.

The structural diversity of TSs arises from their modular biosynthesis. First, TSs are synthesized via the mevalonic acid (MVA) and methylerythritol 4-phosphate (MEP) pathways by the cyclization of 2,3-oxidosqualene, giving rise to their oleanane- (β-amyrin) or dammarane-type triterpenoid skeletons^4,5^. Then the triterpenoid skeletons undergo various modifications, such as oxidation, hydroxylation, or glycosylation, mediated by cytochrome P450 monooxygenases (CYP450s), UDP-glycosyltransferases (UGTs), and other enzymes. The CYP450 and UGT family proteins play essential roles in creating the structural diversity of TSs across plant species^1,2,6,7^. For example, CYP716 family proteins synthesize oleanane-type TSs, platycosides, by catalyzing the C-28 oxidation of β-amyrin in *Platycodon grandiflorus*^7–9^.

*P. grandiflorus* (2*n* = 18), a herbaceous perennial with bell-shaped flowers, belongs to the bellflower family, Campanulaceae. *P. grandiflorus* roots have been used as a popular food additive with therapeutic effects and also as a traditional medicine to treat respiratory diseases (bronchitis, asthma, tonsillitis, and pulmonary tuberculosis) and cold-related symptoms in East Asia for >2000 years^10^. Platycosides, especially platycodin D/E, are the most important bioactive compounds in the root extract of *P. grandiflorus*^8,11^. Studies demonstrating the immunological and pharmacological properties of platycosides have generated considerable clinical interest in these compounds^9^. Platycosides exhibit anti-inflammatory, antiobesity, antiallergy, and antitumorigenic activities and inhibit hepatitis C infection^9^. Thus the level of platycosides in commercial products is the major factor that determines product quality^9^. The platycosides of *P. grandiflorus* are structurally distinct from the dammarane-type TS of *Panax* species, ginsenoside^12,13^. This suggests that *P. grandiflorus* may be a useful medicinal plant model for studying evolution and molecular pathways related to the biosynthesis and production of oleanane-type TS by comparing with *Panax* species. For example, the preferential functions of β-amyrin synthase (*bAS*) in *P. grandiflorus*^7,8^ and dammarenediol synthase (*DDS*)^10^ in *Panax* species may imply that those genes are evolved specifically with the production of platycosides and ginsenosides, respectively. Moreover, CYP716 family is also known to evolve specifically toward the biosynthesis of TS^7^. However, the evolutionary history of platycoside biosynthesis-related genes remains unknown, although ≥70 different platycosides have been isolated from *Platycodon* species^9,10^.

Here we report the whole-genome assembly, transcriptome, and methylome of *P. grandiflorus*. This genome-wide analysis revealed that the *CYP716* and *bAS* gene families play a major role in platycoside biosynthesis with species-specific expansion. Furthermore, the genomic data were supported by the methylome data, indicating the role of epigenetics in platycoside biosynthesis. Overall, our results provide key insights into the evolutionary expansion and transcriptional regulation of platycoside biosynthesis genes in *P. grandiflorus*.

## Results and discussion

### Genome assembly of *P. grandiflorus*

*P. grandiflorus* cultivar Jangbaek-doraji was used for whole-genome sequencing (WGS) after four generations of self-fertilization. Karyotype analysis confirmed that *P. grandiflorus* has a diploid genome (2*n* = 2*x* = 18) with 4 metacentric chromosome pairs (length: 2.19–2.57 μm) and 5 sub-metacentric chromosome pairs (length: 1.91–3.48 μm) (Supplementary Fig. S1). In addition, *k*-mer analysis estimated the genome size of *P. grandiflorus* as approximately 683.3 Mb (Supplementary Fig. S2). A hybrid assembly of short and long reads resulted in a 680.1 Mb draft genome, with 4815 scaffolds (N50 value = 277.1 kb) and 1.35% gaps (Table 1; Supplementary Fig. S3). The draft genome assembly captured 96.9% of the complete Benchmarking Universal Single-Copy Orthologs (BUSCOs) with the Virdiplantae_odb10 database (update date: 20-11-2019) (Supplementary Table S4); 84.9%, 12.0%, 2.4%, and 0.7% of BUSCO genes were predicted as complete and single-copy, complete and duplicated, fragmented, and missing, respectively (Supplementary Tables S4 and S17). However, erroneous assembly by duplication was assessed to be 12% even if showing high level of complete BUSCOs (scores for duplicated BUSCOs are provided in Supplementary Table S18). We also validated the assembly by comparing the read spectrum with the copy number in the assembly using KAT^14^. The *k*-mer showed the homozygous distribution without a certain heterozygous peak (Supplementary Fig. S16), consistent with the low heterozygosity observed by GenomeScope (Supplementary Fig. S2c). Furthermore, the absent *k*-mers (black in *k*-mer comparison plot) at the frequency of average sampling depth was relatively low (Supplementary Fig. S16), suggesting a good quality of assembly completeness. In addition, we aligned short reads to itself. The results showed that 98.1% of the short reads (816,532,377 mapped reads out of 831,574,902 clean raw reads) were successfully re-aligned to the assembly, with 98% effective coverage (Supplementary Fig. S4a, b; Supplementary Table S20), and the unassembled genomic fraction was only 2%. The analysis also showed 0.44% of the nucleotides in the *P. grandiflorus* genome were heterozygous (Supplementary Fig. S4c), indicating the low heterozygous characteristics of the draft genome assembly of *P. grandiflorus*. Overall, the genome assembly of *P. grandiflorus* was of good quality.

**Table 1:** Genome assembly and gene prediction of *Platycodon grandiflorus*

Parameters	Value
Genome assembly	
Scaffold number	4815
Total scaffold length	680.1 Mb
Scaffold N50 value	277,181 bp
Longest scaffold	1,387,349 bp
GC content	36.2%
Gene prediction	
Gene number	40,017
Gene number supported by RNA-Seq	39,188
Mean gene length	5051 bp
Total length of gene models	224.8 Mb
Exons	
Exon number	212,565
Average exon number per gene	4.77
Average exon length	221 bp
Introns	
Intron number	168,058
Average intron number per gene	3.77
Average intron length	1044 bp

### Genome annotation of *P. grandiflorus*

A total of 40,017 non-redundant (NR) protein-coding genes were predicted in the *P. grandiflorus* genome, with an average length of 5051 bp from repeat-masked genomic sequence (Table 1; Supplementary Tables S2 and S3) using evidence-driven gene prediction methods coupled with ab initio prediction. The BUSCO analysis for the gene set showed that 83.3%, 7.1%, 7.8%, and 1.9% of BUSCO genes were predicted as complete and single-copy, complete and duplicated, fragmented, and missing, respectively (the part of gene set in Supplementary Table S4; BUSCO scores are listed in Supplementary Table S19). The gene models were supported by 89.1% PacBio isoform sequencing (Iso-Seq) data, comprising 92,368 assembled isoforms derived from leaf, stem, and root tissues (Supplementary Fig. S6a, b) and 98.1% Illumina RNA-Seq data of *P. grandiflorus* derived from 8 different tissues (Supplementary Fig. S7); these data indicate that genes were predicted with a high level of confidence. Gene annotation revealed a high abundance of genes associated with lipid metabolism and carbohydrate biosynthesis (Supplementary Figs. S8 and S9). This trend was conserved among the genomes of five herbal species, including sunflower (*Helianthus annuus*), coffee (*Coffea canephora*), carrot (*Daucus carota*), and *Panax ginseng*. We also identified a total of 9027 genes encoding transcription factors (TFs) in the *P. grandiflorus* genome, with a predominance of genes encoding basic helix-loop-helix (bHLH) TFs (11%) (Supplementary Fig. S10). Interestingly, four *triterpene saponin biosynthesis activation regulator* (*TSAR*) duplicates in the bHLH family were identified (Supplementary Table S5); the *TSAR* genes regulate β-subunit of tryptophan synthase in alfalfa (*Medicago truncatula*)^15^ and quinoa (*Chenopodium quinoa*)^16^. Among the four gene duplicates, only PGJG172350 (*TSAR2* homolog) was expressed in root, stem, flower, and leaf tissues of *P. grandiflorus* (Supplementary Table S5), suggesting its role in the regulation of platycoside biosynthesis. In addition, evidence-based de novo prediction indicated that 36.2% (248 Mb) of the *P. grandiflorus* genome was composed of transposable elements (TEs) (Supplementary Table S5). This value was lower than that in other Asterid species, including carrot (46%)^17^, coffee (50%)^4^, and *P. ginseng* (80%)^12,13^, suggesting that the genome structure of *P. grandiflorus* is relatively simple.

### Evolution of the *P. grandiflorus* genome in the Asterid lineage

**Fig. 1:**
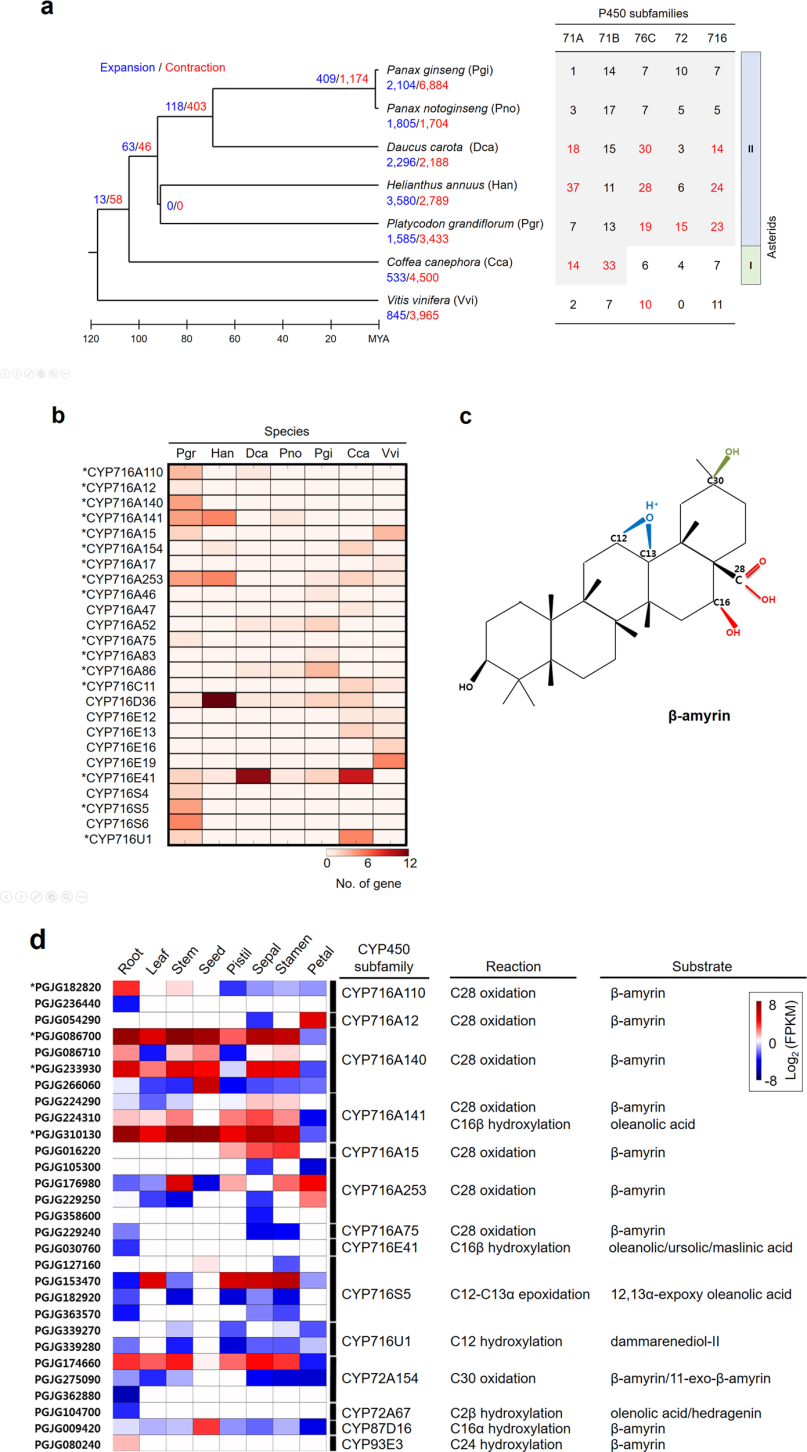
Evolution of the CYP716 family in *P. grandiflorus*. **a** Expansion and contraction of the CYP716 family in *P. grandiflorus* based on a time-calibrated phylogeny of six Asterid species. Branch numbers indicate the number of expanded (blue) and contracted (red) gene families after the split from the most recent common ancestor (MRCA). The table (right) shows the number of CYP450 families identified in Asterids analyzed on the left. In the table, the gray shaded regions indicate expansions of the CYP450 family in the MRCA, and red-color numbers indicate expansions specific to the corresponding species. **b** Heatmap showing the abundance of CYP716 proteins in six Asterids. Asterisks (*) indicate CYP716 proteins that modify triterpene scaffolds. **c** β-Amyrin modifications in *P. grandiflorus*. The structure of PubChem (ID: 73145) was modified to use the β-amyrin scaffold. Different reactions involved in the modification of β-amyrin are indicated in red, blue, and green in **d**. **d** Heatmap showing the expression of *CYP450* genes and known reactions on platycoside scaffolds. Gene expression is presented as log_2_ fragments per kilobase of transcript per million mapped reads (FPKM) of RNA-Seq data from eight different tissues. Asterisks (*) indicate genes with significantly higher expression in roots than in other tissues (*p* < 0.001; Fisher’s exact test)

To understand the evolutionary history of *P. grandiflorus* in the Asterid II lineage, we conducted a genome-wide orthologous gene comparison among Asterid II species, including *P. grandiflorus*, *P. ginseng*^12^, *Panax notoginseng*^5^, carrot^17^, and sunflower^18^ (Supplementary Table S6), while the coffee^4^ genome was used as an Asterid I representative. Of the 25,929 orthologous gene families identified in *P. grandiflorus*, 5498 gene families were conserved among Asterid II species (Supplementary Fig. S11). We then constructed a phylogenetic tree based on 606 single-copy orthologs (Fig. 1a). This tree assigned *P. grandiflorus* in the early Asterid II lineage, consistent with a previous study showing early divergence in the Campanulaceae family based on chloroplast genomes^19^. Based on these findings, we analyzed the expansion and contraction of gene families according to their evolutionary histories. In *P. grandiflorus*, 13 gene families showed expansion relative to the most recent common ancestor (MRCA) of Asterids, which arose 104 million years ago (MYA), and 63 gene families showed expansion relative to the MRCA of Asterids II, which diverged 92 MYA (Fig. 1a). Of the 63 gene families that were expanded relative to the MRCA of Asterids II, 9 gene families contained various functional domains, including zinc knuckle, legume lectin domain, transferase, salt stress response/antifungal, F-box domain, protein kinase domain, CYP450, protein tyrosine kinase, and UDP-glucoronosyl/UDP-glucosyl transferase (Supplementary Figs. S12 and S13). Interestingly, our in-depth annotation revealed expansion of CYP71A/B, CYP72, CYP76C, and CYP716 families shared within the Asterid clade (Supplementary Table S7). For example, CYP71A/B families expanded in the MRCA of Asterids; CYP72, CYP76C, and CYP716 families expanded in the MRCA of Asterids II (right panel in Fig. 1a); and CYP82 and CYP75 families contracted in the MRCA of Asterids II (Supplementary Table S8). Furthermore, the analysis revealed species-specific expansion and contraction of those families (Fig. 1a; Supplementary Table S8), suggesting their recursive expansions and contractions and its genetic divergence. Remarkably, CYP76C, CYP72, and CYP716 families were specifically expanded in *P. grandiflorus*. In the CYP716 family, CYP716A and CYP716S subfamilies were abundant in *P. grandiflorus* relative to other Asterid species (Fig. 1b; Supplementary Table S9). Thus our results provide novel insights into the evolution of the *P. grandiflorus* genome and CYP450 subfamilies.

### Expansion of the *CYP716* family contributes to the diversification of platycoside scaffolds in *P. grandiflorus*

We identified a total of 158 putative TS scaffold-modifying genes, belonging to CYP716, CYP72, CYP87, and CYP93 families, in 7 plant species, including *P. grandiflorus*, *P. ginseng*, *P. notoginseng*, carrot, sunflower, coffee, and grape (Supplementary Table S9). This analysis revealed different distribution patterns of those families in six Asterid species and in grape (*Vitis vinifera*), thus possibly contributing to TS diversification, consistent with previous results^20^. Of the genes identified in *P. grandiflorus* (35 genes out of 158), the *CYP716A* subfamily genes were the most abundant, comprising 19 paralogs within 7 subfamilies, all of which were specialized in the C-28 oxidation of the β-amyrin skeleton (Fig. 1c; Supplementary Table S9). The CYP716A subfamily seems to play a major role in the diversification of scaffolds involved in platycoside biosynthesis in *P. grandiflorus*^7,9^. Three *CYP716A* genes, including *CYP716A12*, *CYP716A140*, and *CYP716A75*, were identified only in *P. grandiflorus*. In addition to the *CYP716A* subfamily, the *CYP716S5* subfamily was also specifically expanded in *P. grandiflorus* (Supplementary Table S9). The CYP716S5 proteins are involved in the epoxidation at C12-C13α of β-amyrin^7^ (Fig. 1c, blue) and play a role in producing the heterocyclic saponin, 12α-hydroxy-β-amyrin-13,28β-lactone^7^. The cytotoxic effects of the heterocyclic saponin have been demonstrated in the human ECA-109 cell line. However, only a small amount of the heterocyclic saponin was isolated from the roots of *P. grandiflorus*^21,22^; thus a better extraction method is needed to isolate a sufficient amount of this chemical for clinical use. The *CYP72A154* subfamily was also specifically expanded in *P. grandiflorus* (Fig. 1c, green**)**^23^; CYP72A154 proteins catalyze the C-30 hydroxylation of the β-amyrin skeleton, although their role in platycoside biosynthesis is not as important as that of CYP716 proteins (Supplementary Table S9). Therefore, our results suggest that the *CYP716* gene family evolved specifically in *P. grandiflorus*, thus contributing to the diversification of platycoside scaffolds, given its oxidation activity.

### Divergent expression of the *CYP716* family genes in different tissues of *P. grandiflorus*

Expression analysis of 35 *CYP716* family genes in different *P. grandiflorus* tissues, including leaf, root, stem, seed, petal, pistil, sepal, and stamen (Supplementary Table S2), revealed tissue-specific expression profiles (Supplementary Table S10). Four *CYP716* family genes including PGJG182820, PGJG233930, PGJG086700, and PGJG310130 showed significantly higher expression in roots than in other tissues (*p* values: 3.8 × 10^−8^, 5.9 × 10^−6^, 5.0 × 10^−14^, 1.3 × 10^−12^, respectively), whereas other *CYP716* family genes were specifically expressed in other tissues; for example, PGJG266060 in seeds (*p* = 1.2 × 10^−49^), PGJG054290 in petals (*p* = 5.2 × 10^−29^), and PGJG016220 in stamen (*p* = 2.9 × 10^−4^) (Fig. 1d). Besides *CYP716* genes, we identified four *CYP716S5* genes, of which PGJG153470 exhibited higher expression in flower tissues, including pistil (*p* = 4.9 × 10^−4^), sepal (*p* = 1.4 × 10^−5^), and stamen (*p* = 3.0 × 10^−16^), than in roots (Fig. 1d). Therefore, the divergent tissue-specific expression patterns of *CYP716* family genes, along with *bAS* genes, lead to the accumulation of platycosides to varying levels in different tissues of *P. grandiflorus*. In addition, our results suggest that *CYP716* paralogs may be sub-functionalized in various tissues for platycoside biosynthesis.

Exogenous application of methyl jasmonate (MeJA or MJ) increases the expression of triterpenoid saponin biosynthesis (TSB)-related genes in *Panax* species^24^. Therefore, we examined the expression of *CYP716* family genes in *P. grandiflorus* plants treated with MJ for 12, 24, and 48 h. Two *CYP716* family genes including PGJG086700 (*CYP716A140*) and PGJG310130 (*CYP716A141*) were highly expressed in roots at all three time points in response to the MJ treatment (Supplementary Table S10), indicating their association with the induction of platycoside biosynthesis^25^. Moreover, both these genes are involved in oleanolic acid biosynthesis, as shown previously^7^. Therefore, our transcriptome data can be useful for researchers to identify and confirm key genes involved in TSB.

### Genomic expansion and divergent expression of TSB-related genes in *P. grandiflorus*

**Fig. 2:**
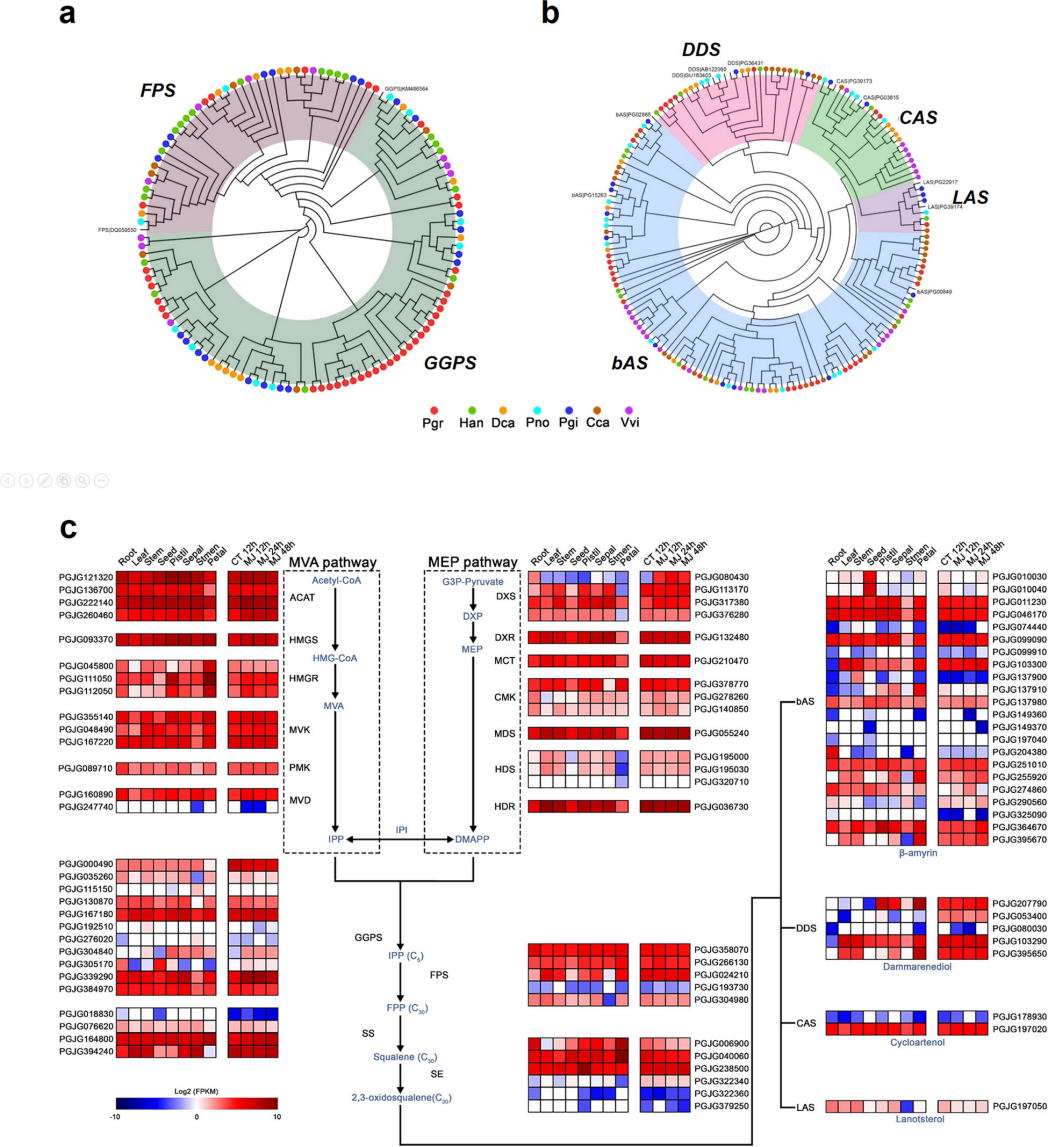
Expansion and diverged expression patterns of triterpenoid saponin biosynthesis (TBS)-related genes in *P. grandiflorus*. **a**, **b** Phylogeny of the *GGPS* (**a**) and *bAS* (**b**) gene families in *P. grandiflorus*. Phylogenetic trees were constructed using the maximum likelihood (ML) method. **c** Expression profiles of TBS-related genes in *P. grandiflorus*. Data represent log_2_ FPKM values of RNA-Seq data generated from eight different tissues, including root, leaf, stem, seed, and four flowers, of plants treated with methyl jasmonate (MJ) for 12, 24, and 48 h (MJ 12 h, MJ 24 h, and MJ 48 h, respectively); CT 12 h indicates the control

We manually selected genes involved in various TSB pathways, including MVA, MEP, isopentenyl pyrophosphate, and OSC pathways (Supplementary Table S11), and analyzed conserved domains in the proteins encoded by these genes (Supplementary Table S12). Based on protein domain and phylogenetic analyses, a total of 827 TSB-related genes were identified in 7 model plant species analyzed in the study (Supplementary Fig. S15; Supplementary Table S13). Furthermore, we analyzed the expression of TSB-related genes in eight tissues of plants treated with MJ for three different time periods (Supplementary Table S14). A similar gene number was identified in the MVA and MEP pathways of six Asterid species and grape (Supplementary Table S13). Interestingly, expansion of the *geranylgeranyl pyrophosphate synthase* (*GGPS*) gene family was identified in *P. grandiflorus* (32 genes, Fig. 2a); these genes function downstream of the MVA and MEP pathways (Fig. 2c). The *GGPS* gene family of *P. grandiflorus* was twofold larger than that of *P. ginseng*^5,12,13,26^. Phylogenetic analysis revealed a *GGPS* gene cluster in *P. grandiflorus* (Fig. 2a), suggesting that the expansion of the *GGPS* gene family in *P. grandiflorus* was relatively recent. Despite the considerable amount of gene duplication in *P. grandiflorus*, many *GGPS* paralogs (20/32) were not expressed in any tissue, being further validated by quantitative reverse transcriptase polymerase chain reaction (qRT-PCR) (Supplementary Table S15) and supported by high correlation between RNA-Seq and qRT-PCR (*r*^2^ = 0.952–1.0 in root, leaf, stem, and flower). Five *GGPS* genes showed significantly higher expression in flowers and leaves than in other tissues (Fig. 2c; Supplementary Tables S14 and S15): PGJG000490 (leaf, *p* = 5.27 × 10^−8^; stamen, *p* = 4.75 × 10^−10^), PGJG339290 (stamen, *p* = 3.80 × 10^−53^), PGJG305170 (seed, *p* = 3.36 × 10^−17^), PGJG304840 (sepal, *p* = 0.0016), and PGJG167180 (stamen, *p* = 1.42 × 10^−12^). We also identified a relatively high number of *bAS* duplicates (24 copies) in *P. grandiflorus* (Fig. 2b, c); these genes synthesize the precursor of oleanane-type TS (β-amyrin) by cyclizing 2,3-oxidosqualene. The *bAS* gene duplicates also showed tissue-specific expression profiles. Of the 24 *bAS* genes, 4 genes, including PGJG046170, PGJG204380, PGJG290560, and PGJG099090, showed significantly higher expression in roots than in other tissues (*p* values: 3.67 × 10^−4^, 1.11 × 10^−53^, 1.89 × 10^−5^, and 5.5 × 10^−3^, respectively; Fig. 2c). In addition, we identified four *dammarenediol synthase* (*DDS*) genes in the *P. grandiflorus* genome (Fig. 2b, c); the *DDS* genes synthesize the scaffold of the dammarane-type TS enriched in *Panax* species^13^. However, all of these *DDS* genes showed low level of expression in roots, which may be associated with less attention paid to the dammarane-type TS in *P. grandiflorus*. Altogether, our results demonstrate the expansion of *GGPS* and *bAS* gene families in *P. grandiflorus*, and their divergent expression patterns in various tissues, similar to the *CYP716* family genes. In addition, the identification of TSB-related candidate genes with tissue-specific expression profiles may help elucidate their targets and molecular mechanism underlying platycoside biosynthesis (Fig. 2c).

### Hypomethylation of *CYP716* and *bAS* genes of *P. grandiflorus*

**Fig. 3:**
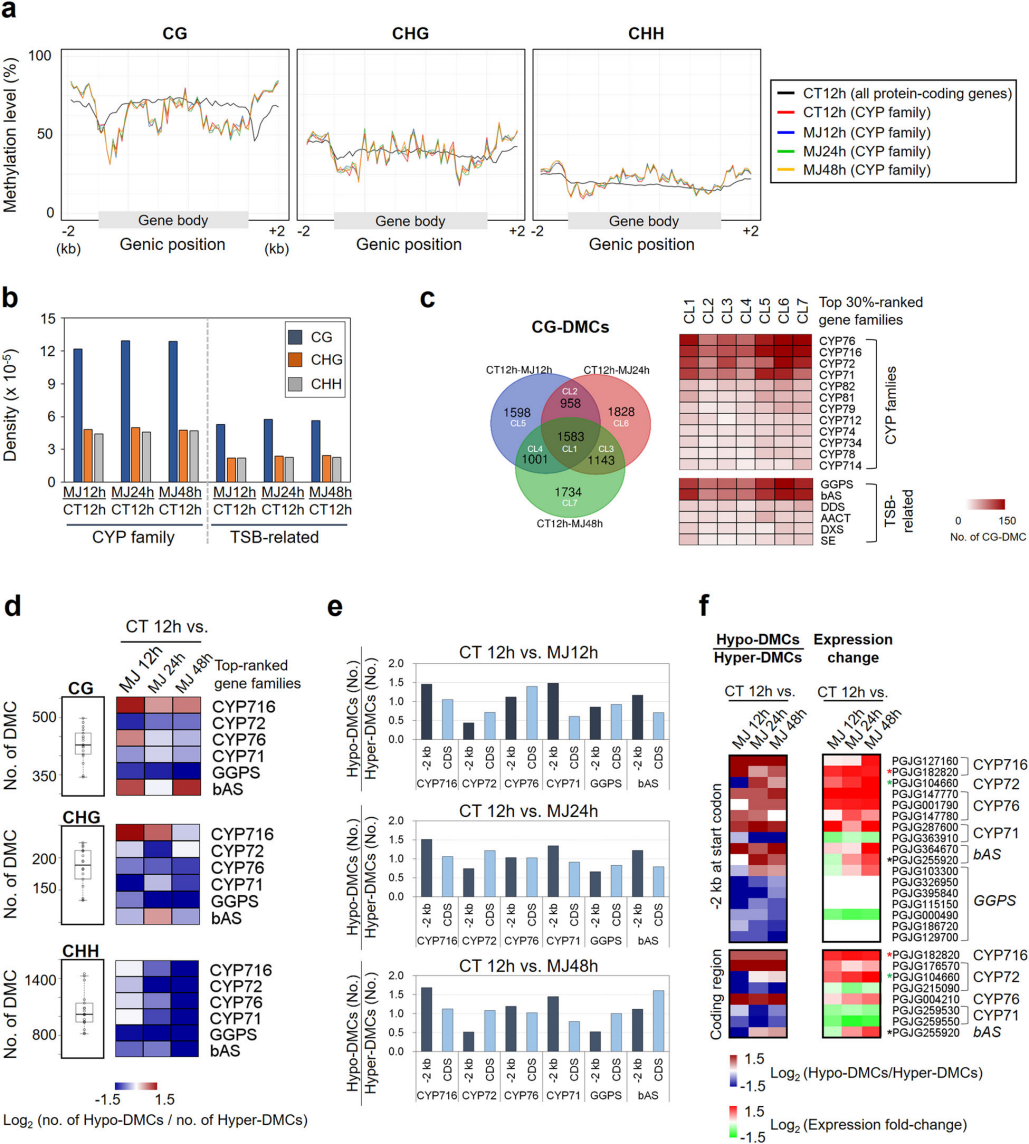
DNA methylation landscape of *CYP450* family and other TSB-related genes in *P. grandiflorus* genome. **a** Methylation status of the *CYP450* family genes in the CG, CHG, and CHH contexts. Based on the distribution of methylation levels of all protein-coding genes (including gene bodies and ~2-kb upstream and downstream regions) in the control sample (CT 12 h_AllGenes; black line), the methylation levels of *CYP450* genes were measured in the control treatment (CT 12 h; red) and at three time points in the MJ treatment (MJ 12 h [blue], MJ 24 h [green], and MJ 48 h [orange]). **b** Density of differentially methylated cytosines (DMCs) in all three contexts in *CYP450* and other TSB-related genes between the control and MJ treatments (CT vs. MJ 12 h, CT vs. MJ 24 h, and CT vs. MJ 48 h). **c** Abundance of CG-DMCs in the top 30% *CYP450* and TSB-related genes in the MJ treatment. **d** Relative abundance of hypermethylated and hypomethylated cytosines (hyper-DMCs and hypo-DMCs, respectively) in the CG context of *CYP716s*, *CYP72s*, *CYP76s*, *CYP71s*, *GGPSs*, and *bAS* in the MJ treatments. The ratio was transformed to the log_2_ scale. **e** Relative abundance of hyper-DMCs and hypo-DMCs in the CG context within ~2-kb upstream regions and coding regions of the primary *CYP450* and TSB-related genes. **f** Identification of genes showing inverse correlation between CG methylation levels and gene expression in the MJ treatment. Asterisk (*) indicates a gene with similar DMC distribution in the ~2-kb upstream region and coding region

To understand the link between DNA methylation and TSB regulation in *P. grandiflorus*, we performed whole-genome bisulfite sequencing (WGBS) and analyzed the DNA methylation status of TSB-related genes. We generated 7.5–10.7 Gb of whole-genome methylome data from MJ-treated *P. grandiflorus* samples harvested at 3 time points (12, 24, and 48 h), covering >90% of all cytosine nucleotides (Supplementary Table S16). Average global methylation levels were estimated at 89.1%, 72.4%, and 18.9% in the CG, CHG, and CHH contexts, respectively, of a control (CT 12 h) and 3 MJ treatments (MJ 12 h, MJ 24 h, and MJ 48 h) (Supplementary Table S16). The average methylation levels of *CYP450* family and other TSB-related genes differed from those of the remaining protein-coding genes (Fig. 3a); the CG methylation level of *CYP450* family genes was lower in gene bodies but higher within ~2-kb upstream and downstream regions, whereas the CHG and CHH methylation levels were slightly increased in gene bodies. However, we could not find any global changes in methylation levels between control and MJ treatments (Fig. 3a), indicating only a small number of differentially methylated cytosines (DMCs). The *CYP450* and other TSB-related genes showed higher density of CG methylation than that of CHG/CHH methylation (Fig. 3b), indicating the epigenetic role of CG methylation in TS biosynthesis. Of the 9845 CG-DMCs identified between control and MJ treatments, 5140, 5512, and 5461 CpG sites showed differential methylation levels in the MJ treatment at 12, 24, and 48 h, respectively (Fig. 3c, top panel). The CG-DMCs were the most predominant among the *CYP76* (8.9%), *CYP716* (8.7%), *CYP72* (7.4%), *CYP71* (6.9%), *GGPS* (8.0%), and *bAS* (7.7%) genes, which were abundant within the top 30% *CYP450* families and other TSB-related genes (Fig. 3c, bottom panel). In addition, 16% of all CG-DMCs were detected at all three time points of the MJ treatment (Fig. 3c), indicating continuous changes in CG methylation.

We further examined the relative abundance of hypomethylated cytosines (hypo-DMCs) and hypermethylated cytosines (hyper-DMCs) in the *CYP450* family and other TSB-related genes. Among the six gene families ranked within the top 30%, the *CYP716* and *bAS* gene families showed relatively lower CG methylation in the MJ treatment (Fig. 3d). Furthermore, the relative dominance of hypo-CG-DMCs in the MJ treatment was detected at all three time points in the upstream and coding regions of *CYP716* family genes (Fig. 3e) but only at 48 h in the upstream and coding regions of *bAS* genes (Fig. 3e). We also identified 22 genes, belonging to *CYP716*, *CYP72*, *CYP71*, *bAS*, and *GGPS* families, showing inverse correlation between CG methylation and gene expression in the MJ treatment (Fig. 3f). This inverse correlation was detected in the upstream and coding regions of two genes, including PGJG182820 (*CYP716A*) and PGJG255920 (*bAS*) (Fig. 3f, asterisk), suggesting a possible connection between epigenetic modification and transcriptional changes. In addition, we observed the relative dominance of hyper-CG-DMCs in the upstream regions of *GGPSs*, resulting in low or no expression of these genes (Fig. 3f). Overall, our results suggest that *CYP716* and *bAS* family genes are likely hypomethylated in *P. grandiflorus*, indicating that epigenetic changes in both these gene families affect platycoside biosynthesis.

## Conclusions

*P. grandiflorus* is used as a popular medicinal and dietary resource throughout East Asia, given its therapeutic effects. Compared with the previously published genomes of six Asterid species, the draft genome assembly of *P. grandiflorus* revealed an expansion of *CYP716* and *bAS* gene families during evolution, which may explain the biosynthesis of platycosides and their divergent chemical structures. The transcriptome data were useful for identifying essential TSB-related candidate genes associated with the production of platycosides in various tissues, especially roots. Furthermore, WGBS provided evidence suggesting epigenetic modification of *CYP716* and *bAS* genes. Thus the *P. grandiflorus* genome will serve as a valuable resource for studying genes needed to improve agricultural traits and platycoside production efficiency through molecular breeding.

## Materials and methods

### Plant material and WGS

*P. grandiflorus* cultivar Jangbaek-doraji was used in this study after four generations of self-fertilization. Plants of *P. grandiflorus* were grown for 1 year in a bellflower field in the Department of Herbal Crop Research, Rural Development Administration (RDA), Republic of Korea. Karyotyping of *P. grandiflorus* was performed by fluorescence in situ hybridization analysis using two probes, 5S rDNA and 45S rDNA. Genomic DNA was isolated from young leaves of *P. grandiflorus* plants using the DNeasy Plant Mini Kit (Qiagen, USA). WGS of *P. grandiflorus* was performed using the Illumina HiSeq 2500 platform and TruSeq Synthetic Long Read (TSLR) technology to generate short reads (insert sizes: 270, 500, 700, and 360 bp) and long reads (average read length: 6.8 kb), respectively, according to the manufacturer’s instructions (Illumina, Inc., San Diego, CA) (Supplementary Table S1). The Illumina HisSeq 2500 platform was also used to generate long mate pair reads (insert sizes: 2, 5, and 10 kb) for generating scaffolds (Supplementary Table S1). The WGS of *P. grandiflorus* generated 325.5 Gb of Illumina short reads (476.4× coverage) and 4 Gb of TruSeq synthetic long reads (TSLRs; 5.9× coverage) (Supplementary Table S1).

### Genome assembly

The genome size of *P. grandiflorus* was estimated by the *k*-mer frequency analysis using SOAPec^28^ (version 2.01), Jellyfish (version 2.2.0), and GenomeScope (http://qb.cshl.edu/genomescope)^29^. For *k*-mer analysis, sequencing errors in short-read libraries (Supplementary Table S1) were corrected using SOAPec (version 2.01). After error correction, a peak (31 depth) in the *k*-mer depth distribution was identified. The whole genome of *P. grandiflorus* was assembled de novo using a hybrid approach involving short and long reads (Supplementary Fig. S3; Supplementary Table S3). A total of 132 Gb of short reads were error-corrected using SOAPec^28^ and assembled into contigs using SOAPdenovo2 (version 2.04)^28^, generating 1,450,347 contigs (528 Mb) with an N50 value of 3.5 kb. Long mate pair reads (193 Gb) were used to join contigs into scaffolds using SOAPdenovo2, and gaps within scaffolds were filled using GapFiller^30^, generating a total of 18,379 scaffolds (633 Mb; N50 = 363 kb). TSLRs (3.99 Gb) were separately assembled into contigs using Celera Assembler (version 8.3)^31^, generating 68,092 contigs (633 Mb; N50 = 15 kb). Assemblies generated from short and long reads were merged using GARM (version 0.7.5) and CAP3. The resulting contigs were further scaffolded with short reads, long reads, and long mate pair reads using SSPACE (version 3.0)^32^ and SSPACE-LongRead (version 1.1), followed by gap filling using GapFiller (version 1.10)^30^. The completeness of the genome assembly was assessed using BUSCO (version 4.0.5)^33^ with 425 BUSCOs of viridiplantae_odb10 database. The workflow of de novo assembly is summarized in Supplementary Fig. S3 and Supplementary Table S3.

### RNA-Seq and data analysis

Total RNA was isolated from eight tissues of *P. grandiflorus*, including leaves, stems, roots, petals, sepals, pistils, stamens, and seeds, and seedlings of *P. grandiflorus* were treated with MJ for 12, 24, and 48 h, with a control, using a total RNA extraction kit (Intron Biotechnology, Korea). RNA-Seq libraries were prepared from those RNA samples using the TruSeq Stranded mRNA Library Prep Kit (Illumina, Inc., San Diego, CA, USA) according to the manufacturer’s protocol. Paired-end sequencing with 100 cycles was performed using an Illumina HiSeq 2500 (Supplementary Table S2). Clean reads were mapped onto the draft genome of *P. grandiflorus* using TopHat. Gene expression levels were estimated in terms of FPKM (fragments per kilobase of transcript per million mapped reads) values using Cufflinks^27^ based on gene annotations, and tissue-specific expression levels were compared using Fisher’s exact test. Genes differentially expressed between the control (CT 12 h) and MJ treatments (MJ 12 h/24 h/48 h) (*p* < 0.01; |fold-change| ≥ 1.5) were analyzed using Cuffdiff^27^.

### Gene prediction and gene annotation

A combination of ab initio and evidence-based approaches was used for gene prediction. The genome assembly was pre-masked for repetitive DNA sequences using RepeatMasker (version 4.0.6) (http://www.repeatmasker.org/). An unsupervised training gene structure was generated using GeneMark-ET (version 4.10)^34^ by incorporating RNA-Seq and Iso-Seq data. Gene prediction was further performed using AUGUSTUS (version 3.3.1)^35^, based on gene structure information, such as exon–intron boundaries, predicted from RNA-Seq and homologous protein sequence alignment data. Gene models were predicted based on RNA-Seq and Iso-Seq data using TopHat (version 2.1.1)^36^, and amino acid sequence alignments with the NCBI NR protein database were performed using Exonerate (version 2.4.0)^37^. To validate the accuracy of gene models, RNA-Seq and Iso-Seq data were aligned with gene models using TopHat and GMAP^38^. To perform functional gene annotation, gene models were searched against UniProt, NCBI NR, and Plant RefSeq databases using BLASTP (version 2.3.0+) (*E*-value cutoff = 1*E* − 5). Protein domains were searched using InterProScan (version 5.19–58.0). Functional annotation of gene models was performed using gene ontology (www.geneontology.org) enrichment analysis, Kyoto Encyclopedia of Genes and Genomes (https://www.genome.jp/kegg) database, Plant Metabolic Pathway database (https://www.plantcyc.org), and Plant Transcription Factor Database (PlantTFDB; http://planttfdb.cbi.pku.edu.cn/). TE sequences were identified using RepeatMasker. To identify TE-related coding sequences, non-repeat masked assembled sequences were searched against the MIPS Repeat Element Database (mipsREdat_9.3p; http://www.transplantdb.eu/node/2249) using TBLASTX and BLASTN (version 2.3.0+) (*E*-value cutoff = 1*E* − 20).

### Gene family expansions and contractions

Genomic resources of one Asterid I species (*Coffea canephora* [Cca]^4^); four Asterid II species, including *P. ginseng* (Pgi)^12^, *P. notoginseng* (Pno)^5^, *D. carota* (Dca)^17^, and *H. annuus* (Han)^18^; and one outgroup species (*V. vinifera* [Vvi])^39^ were used to investigate the genomic and evolutionary features of *P*. *grandiflorus*. After removing non-canonical genes (i.e., genes with premature stop codons in the coding region or wrong codon length), all-vs.-all BLASTP searches (*E*-value cutoff <1*E* − 5) were performed, and clustering was conducted using the Markov cluster algorithm (MCL), with an inflation value (−*I*) of 1.5. Orthologous gene families were then analyzed using OrthoMCL (version v2.0.9)^40^. Single-copy orthologs (606 genes) were aligned using MUSCLE (version 3.8), and poorly aligned regions were trimmed using trimAI. A phylogenetic tree was constructed using the RAxML (version 8.2.8)^41^. The divergence time was obtained from the TimeTree database^42^. Gene family expansion throughout the plant lineage was analyzed using the CAFÉ (version 4.0)^43^. Functional domains were defined by Pfam (version 27.0), and enriched domains were estimated by Fisher’s exact test (adjusted *p* value <0.01) and odds ratio ≤2.

### WGBS and data analysis

Genomic DNA was extracted from control (CT 12 h) and MJ-treated (MJ 12 h, MJ 24 h, and MJ 48 h) *P. grandiflorus* seedlings, and libraries for WGBS were prepared as described previously^44^, according to the manufacturer’s instructions (Illumina Inc., San Diego, CA, USA). The WGBS libraries were sequenced on the Illumina HiSeq 2500 platform, generating 100-bp paired-end reads (Supplementary Table S16). Adapters and low-quality bases were trimmed using Trimmomatic (version 0.39). The trimmed reads were mapped onto the draft genome assembly of *P. grandiflorus* using Bismark (version 0.19.1)^45^, with a cutoff at ≥5 read depth. After filtering duplicate reads, the methylation status of each cytosine nucleotide was determined. The methylation data were further evaluated using the binomial test, followed by Benjamini–Hochberg false discovery rate (FDR) correction (FDR < 0.01)^46^. The methylation level was calculated using the following equation:

$${\mathrm{mC/}}\left( {{\mathrm{mC + umC}}} \right)$$

where mC and umC represent the number of methylated and unmethylated reads, respectively, in three contexts (CG, CHG, and CHH).

To measure the average methylation levels in protein-coding genes, the coordinates of upstream sequences (2 kb upstream of the start codon), downstream sequences (2 kb downstream of the stop codon), and gene bodies (including exons and introns) were extracted and divided into 10, 10, and 50 bins, respectively, and the average methylation level of each bin was calculated and plotted. To identify DMCs between control and MJ treatments in protein-coding regions, sites with |ΔmC| ≥ 10 were selected; the value of |ΔmC| was calculated using the following equation:

$$\left| {{\mathrm{mC}}_{{\mathrm{Control}}\_{\mathrm{12h}}} - {\mathrm{mC}}_{{\mathrm{MJ}}\_{\mathrm{12h/24h/48h}}}} \right| \ge 10.$$

## Supplementary information

Supplementary Information

Supplementary Table

## Data Availability

Sequencing data used in this study are available in the NCBI Sequence Read Archive (SRA) database under the following accession numbers: SPEA00000000 (WGS data), SRR8712510–SRR8712517 (RNA-Seq data derived from eight different tissues), SRR8712518–SRR8712529 (RNA-Seq data from MJ treatments), and SRR9005109–SRR9005120 (WGBS data from MJ treatments). In addition, the gene set of *P. grandiflorus* is available from our website http://platycodon.theragenetex.com.
