# Supplementary material for: Whole-genome, transcriptome, and methylome analyses provide insights into the evolution of platycoside biosynthesis in Platycodon grandiflorus, a medicinal plant
Source: Hortic Res. 2020 Jul 1;7:112. doi: 10.1038/s41438-020-0329-x (PMC7327020; doi:10.1038/s41438-020-0329-x)
Supplement: Supplementary file 1 — Supplementary Information [file 41438_2020_329_MOESM1_ESM.pdf]

SUPPLEMENTARY INFORMATION

SUPPLEMENTARY FIGRES

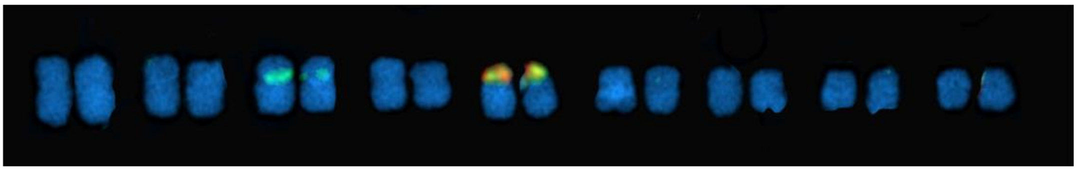

Chromosome number	Chromosome length			Chromosome type
	Short arm (S)	Long arm (L)	Total (S+L)	
1	1.30	2.18	3.48	sm[1]
2	1.28	1.88	3.17	sm
3	1.03	1.76	2.78	sm
4	1.13	1.45	2.57	m[2]
5	1.00	1.34	2.34	m
6	1.06	1.15	2.21	m
7	1.03	1.16	2.19	m
8	0.63	1.35	1.98	sm
9	0.80	1.13	1.91	Sm
Total	9.26	13.40	22.66	4m + 5 sm

[1] sm: Submetacentric chromosome; [2] m: Metacentric chromosome

Supplementary Figure S1. Karyotype of *Platycodon grandiflorus* cv. Jangbaek-doraji. Fluorescence in situ hybridization was conducted using 5S (green) and 45S (red) rDNA probes.

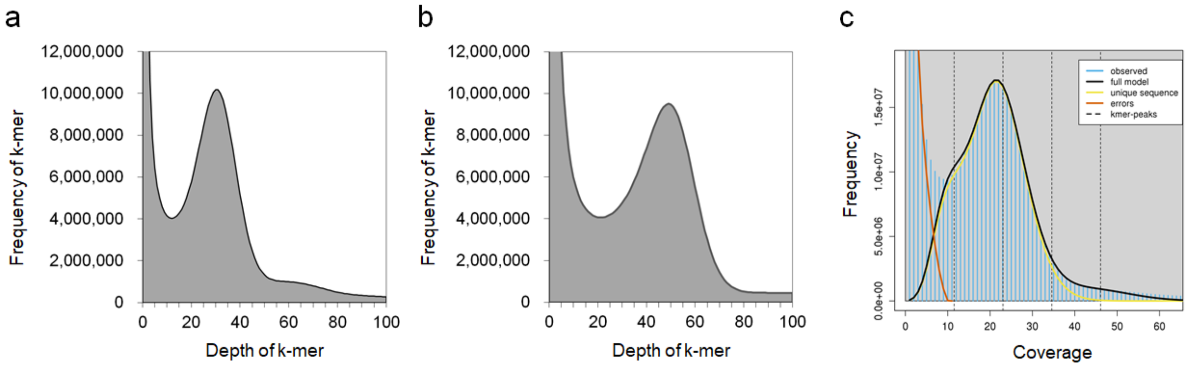

Supplementary Figure S2. *K*-mer analysis of *P. grandiflorus* genome. *K*-mer profile using SOAPec (a), Jellyfish (b), and GenomeScope 2.0 (c).

<u>Supplementary Note for Supplementary Figure S2</u>:

Based on the peak depth of *k*-mer and total number of *k*-mer, the genome size of *P. grandiflorus* was estimated to be approximately 694.4 Mbp for SOAPec, 691.7 Mbp for Jellyfish, and 683.3 Mbp for GenomeScope 2.0.

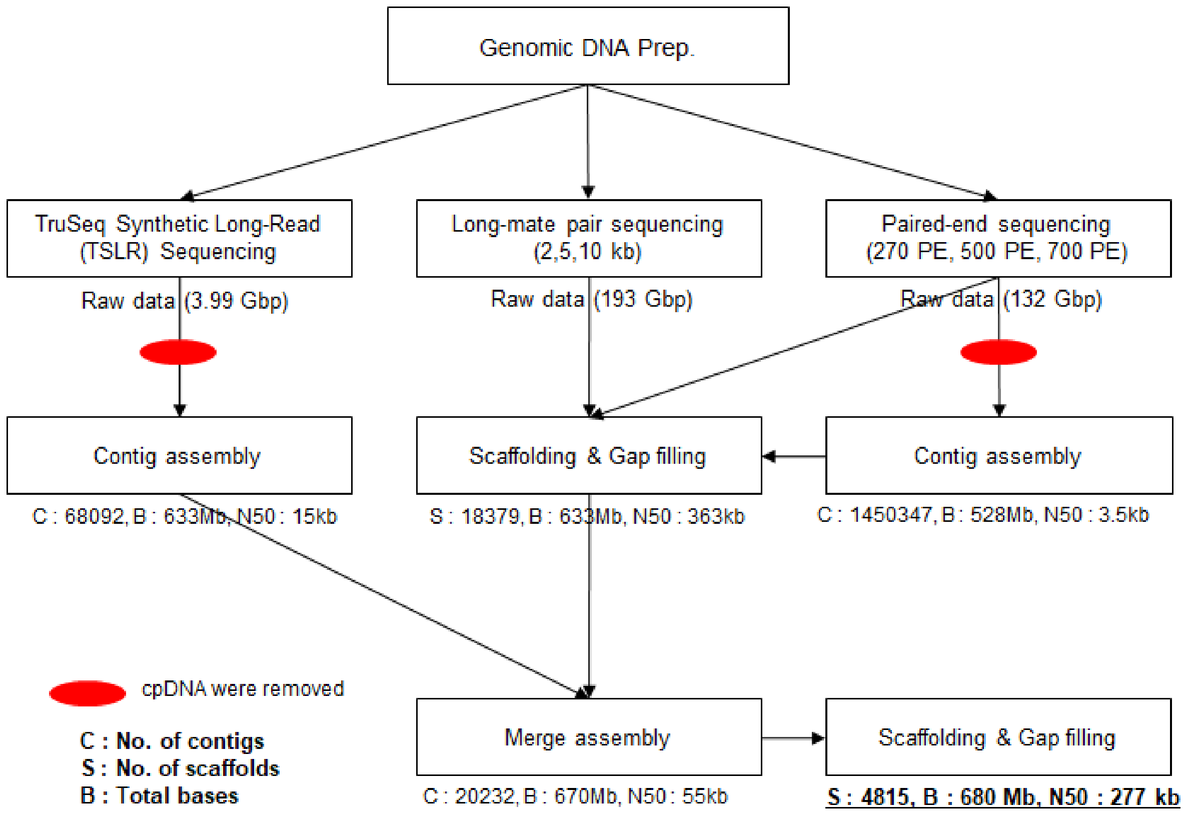

Supplementary Figure S3. Workflow of whole-genome sequencing (WGS) and *de novo* assembly of *P. grandiflorus*.

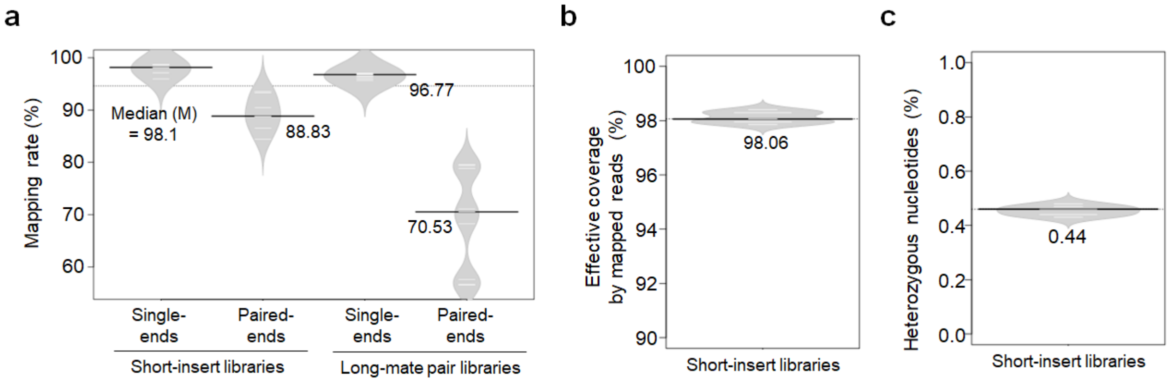

Supplementary Figure S4. Statistics of the re-alignment of short-insert reads and long mate-pair reads to the draft genome assembly of *P. grandiflorus* cultivar Jangbaek-doraji. **(a)** Mapping rate of Illumina short-insert reads and long-mate pair reads. **(b)** Effective coverage by mapped reads. The effective coverage of the draft genome assembly is defined as the actual amount of bases covered by short-insert reads. **(c)** The distribution of heterozygous nucleotides in the draft genome assembly.

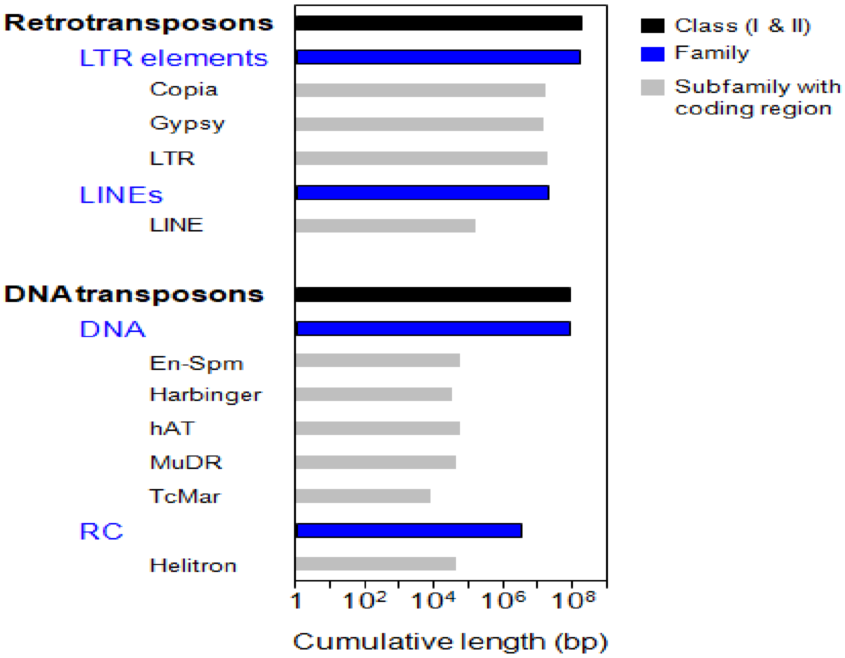

Supplementary Figure S5. Abundance of transposable elements (TEs) identified in the genome of *P. grandiflorus*.

Supplementary Note for Supplementary Figure S5:

The identified TE sequences were classified as retrotransposons or DNA transposons with more detailed families. Approximately 247.5 Mb (36.2% of the genome) was identified as TEs using RepeatMasker[1]. To identify the coding sequences of TEs, the genome assembly of *P. grandiflorus* was searched against the MIPS Repeat Element Database (mipsREdat_9.3p; http://www.transplantdb.eu/node/2249) using TBLASTX and BLASTN with a cutoff at 1×10^{-20}. The searched TE sequences were annotated with reference to the Gypsy database (Gydb; http://gydb.org/index.php/Main_Page). These TEs accounted for approximately 49.9 Mb in cumulative length. Among these TEs, long terminal repeat (LTR) retrotranspons were the most

predominant, especially *Ty3/Gypsy* and *Ty1/Copia* (8.49% and 10.03% of retrotransposons, respectively) (Fig. S5). In addition to LTRs, DNA transposons were also abundant, including En-Spm, hAT, MuDR, and Harbinger (0.07%, 0.06%, 0.05%, and 0.04% of all DNA transposons, respectively).

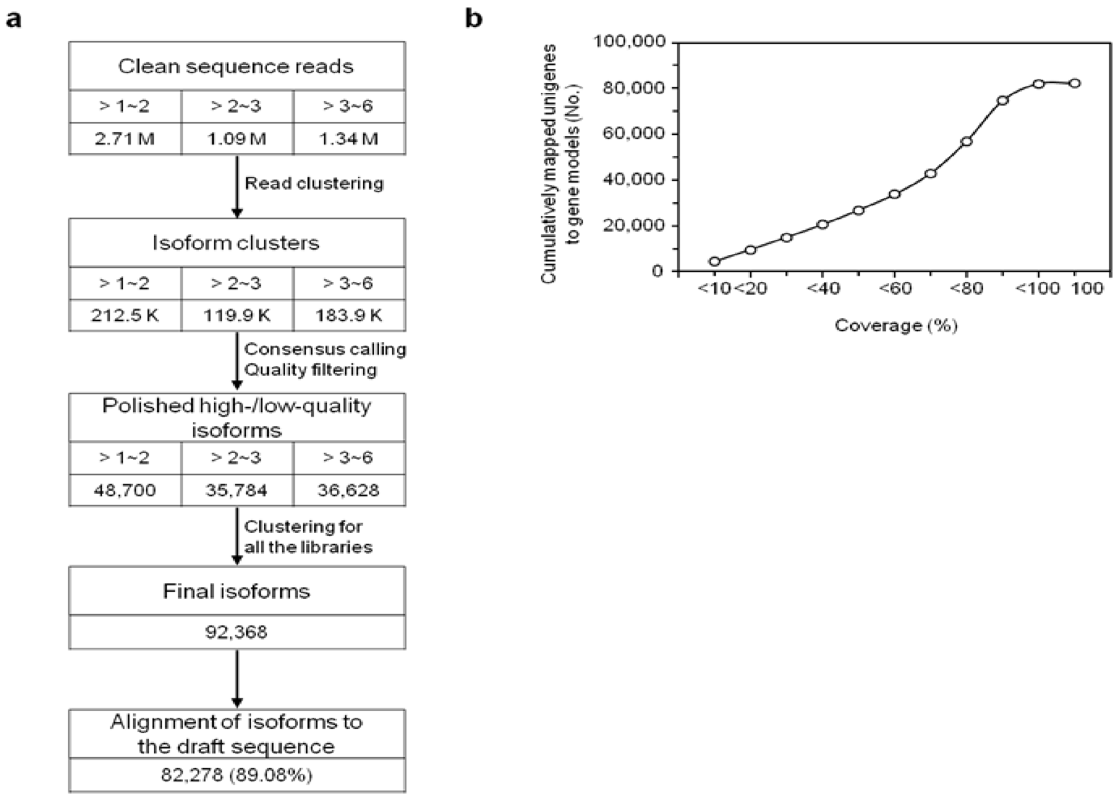

Supplementary Figure S6. Statistics of isoform sequencing (Iso-Seq) data. (a) Workflow showing transcript assembly using Iso-Seq. (b) Examination of the coverage of unigenes for gene models predicted from the whole-genome assembly of *P. grandiflorus*.

Supplementary Note for Supplementary Figure S6:

To obtain long transcripts, cDNAs ranging in size from 1–2, 2–3, and 3–6 kb were selected from pooled RNA samples of three tissues, including leaves, stems, and roots. A total of 5.14 million sequencing subreads were generated using the SMRT sequencing technology (PacBio sequencing), and the reads were merged to form 35,784 to 48,700 isoform clusters using the SMRT-Analysis software (version 2.3.0). After consensus sequence calling and quality filtration, the assembled transcripts or unigenes were clustered into a total of 92,368 isoforms

(N50 = 3,321 bp) using CD-HIT, with a sequence identity threshold of 0.99, based on the methodology described by Jo et al. (2017)[2]. Unigenes were aligned to the draft assembly of *P. grandiflorus* with predicted gene models using GMAP, which improved the overall accuracy of gene prediction by allowing high-precision gene annotation[3]. Out of 92,368 isoforms, 82,278 isoforms (89.08%) mapped to the predicted gene models.

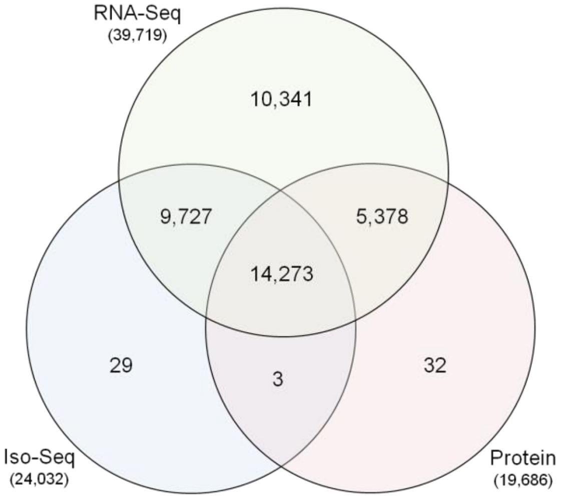

Supplementary Figure S7. Venn diagram showing the number of gene models supported by RNA-Seq data, Iso-Seq data, and homologous protein sequence alignment.

Supplementary Note for Supplementary Figure S7:

Among the evidence-based gene models of *P. grandiflorus*, 39,719 models were supported by RNA-Seq data, 24,032 by Iso-Seq data, 19,686 models by protein sequence alignment, and 700 models by *ab initio* prediction. Most of the gene models (98.2%)were covered by transcriptome data of *P. grandiflorus*. Although some genomes belonging to the Asterid clades, including *Panax ginseng*, *Daucus carota*, and *Helianthus annuus*, have been sequenced, the homology-based gene prediction method possibly reduced the accuracy of gene prediction because of sequence divergence between clades. Thus, transcriptome sequencing in eight various tissues of *P. grandiflorus* using different technologies likely increased the accuracy of gene prediction with the identification of exon-intron boundaries.

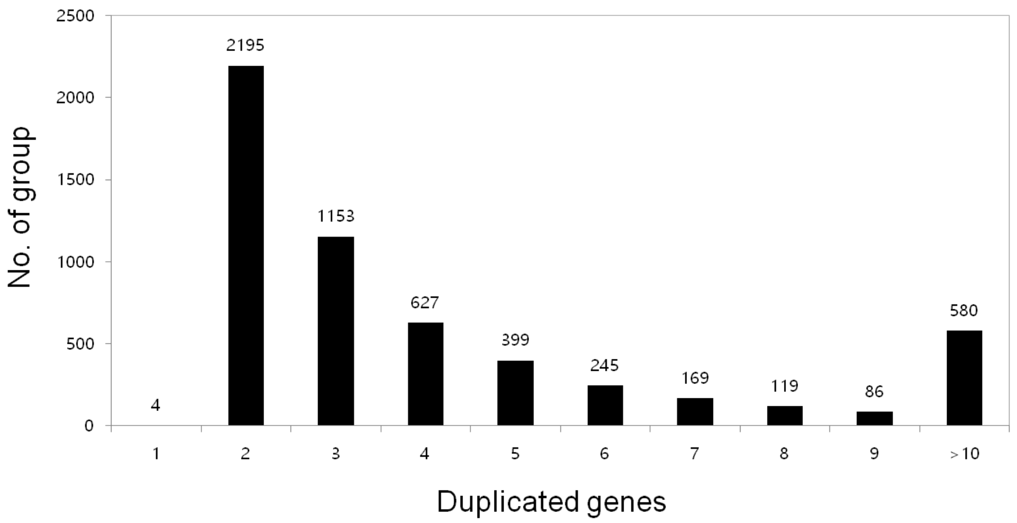

Supplementary Figure S8. Distribution of gene duplicates in the *P. grandiflorus* genome.

<u>Supplementary Note for Supplementary Figure S8</u>:

The distribution of gene duplications in the genome was examined by using OrthoMCL[4]. Most of the genes in *P. grandiflorus* were duplicated with more than two or three copies (E-value < 1×10^{-5}), accounting for approximately 60%. This indicates that the genome of *P. grandiflorus* underwent multiple rounds of whole-genome duplication, followed by myriad fractionation[5].

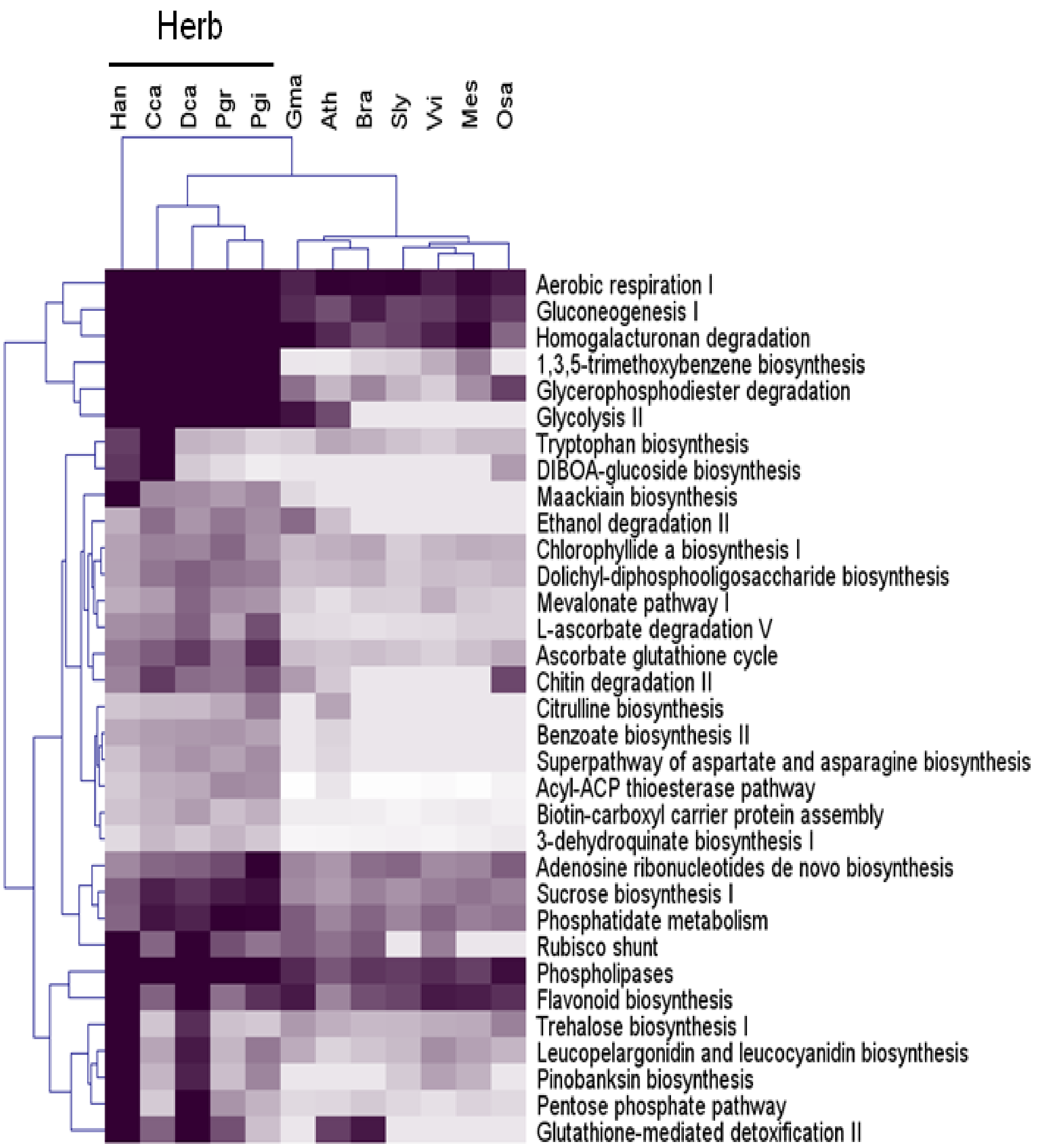

Supplementary Figure S9. Abundance of *P. grandiflorus* genes related to metabolic pathways. Gene models of 12 plant genomes including *Arabidopsis thaliana* (Ath), *Panax ginseng* (Pgi), *Daucus carota* (Dca), *Helianthus annuus* (Han), *Coffea canephora* (Cca), *Glycine max* (Gma), *Brassica rapa* (Bra), *Solanum lycopersicum* (Sly) *Vitis vinifera* (Vvi), *Manihot esculenta* (Mes), and *Oryza sativa* (Osa) were searched against Plant Metabolic Pathway database using BLASTP[6], with an *E*-value cutoff of 1 x 10^{-5}. The number of hits for each species was

transformed into a Z-score. Hierarchical clustering of Z-scores was performed with MeV (http://mev.tm4.org) using Euclidean distance and complete linkage method.

Supplementary Note for Supplementary Figure S9:

The results revealed an abundance of genes involved in gluconeogenesis, homogalacturonan degradation, 1,3,5-trimethoxybenzene biosynthesis, glycerophosphodiester degradation, glycolysis, sucrose biosynthesis, and phosphatidate metabolism, and genes encoding phospholipases (Supplementary Fig. S9). These data suggest that herbal plants, including *H. annuus*, *C. canephora*, *D. carota*, and *P. ginseng*, contains abundant genes associated with lipid metabolism and glucose biosynthesis. In addition, genes related to mevalonate pathway, also known as the isoprenoid pathway or HMG-CoA reductase pathway, were more abundant in herbal plant genomes than in non-herbal genomes.

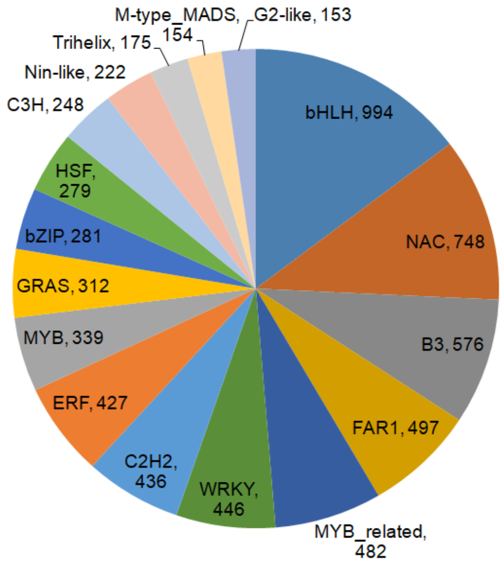

Supplementary Figure S10. Top 30% transcription factor (TF)-related Pfam domains in the genome of *P. grandiflorus*.

<u>Supplementary Note for Supplementary Figure S10</u>:

TFs in *P. grandiflorus* were identified by searching the Plant Transcription Factor Database (PlantTFDB)[7] using BLASTP[6] (*E*-value cutoff = 1 x 10^{-5}). A total of 9,027 gene models with TF-related Pfam domains were categorized into 57 known TF families. The top 30% of these families included bHLH, NAC, B3, FAR1, MYB_related, WRKY, C2H2, ERF, MYB, GRAS, bZIP, HSF, C3H, Nin-like, trihelix, M-type_MADS, and G2-like TFs. Among these, the bHLH family is known as one of the largest TF families found in eukaryotic organisms; bHLH TFs are involved in diverse regulatory processes, especially the regulation of secondary metabolism[8]. Chu et al.[9] recently reported the possibility that some bHLH genes in *P. ginseng* are involved in the regulation of ginsenoside biosynthesis.

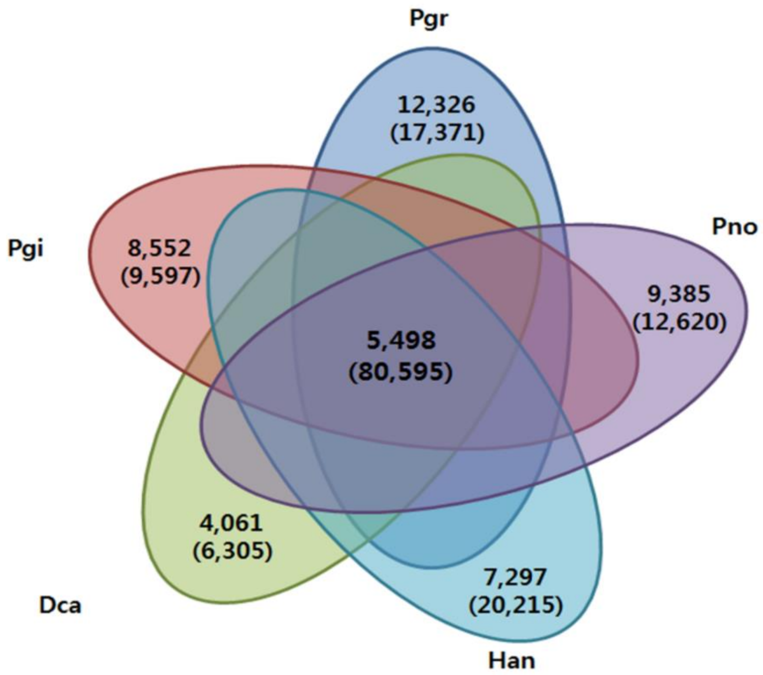

Supplementary Figure S11. Relationship among Asterid II species. Orthologous gene clusters in Asterid II plants were constructed using seven species listed in **Supplementary Table S6**. Gene families in only five Asterid II species are displayed in this figure. Pgr, *P. grandiflorus*; Pgi, *Panax ginseng*; Pno, *Panax notoginseng*; Dca, *Daucus carota*; Han, *Helianthus annuus*.

Supplementary Note for Supplementary Figure S11:

The OrthoMCL analysis identified 66,166 gene families comprising 240,337 (33.53%) genes among seven plant species. Among these, 5,498 gene families (80,595 genes) were common to all Asterid II species, while 12,326 gene families (17,371 genes) were observed only in *P. grandiflorus*.

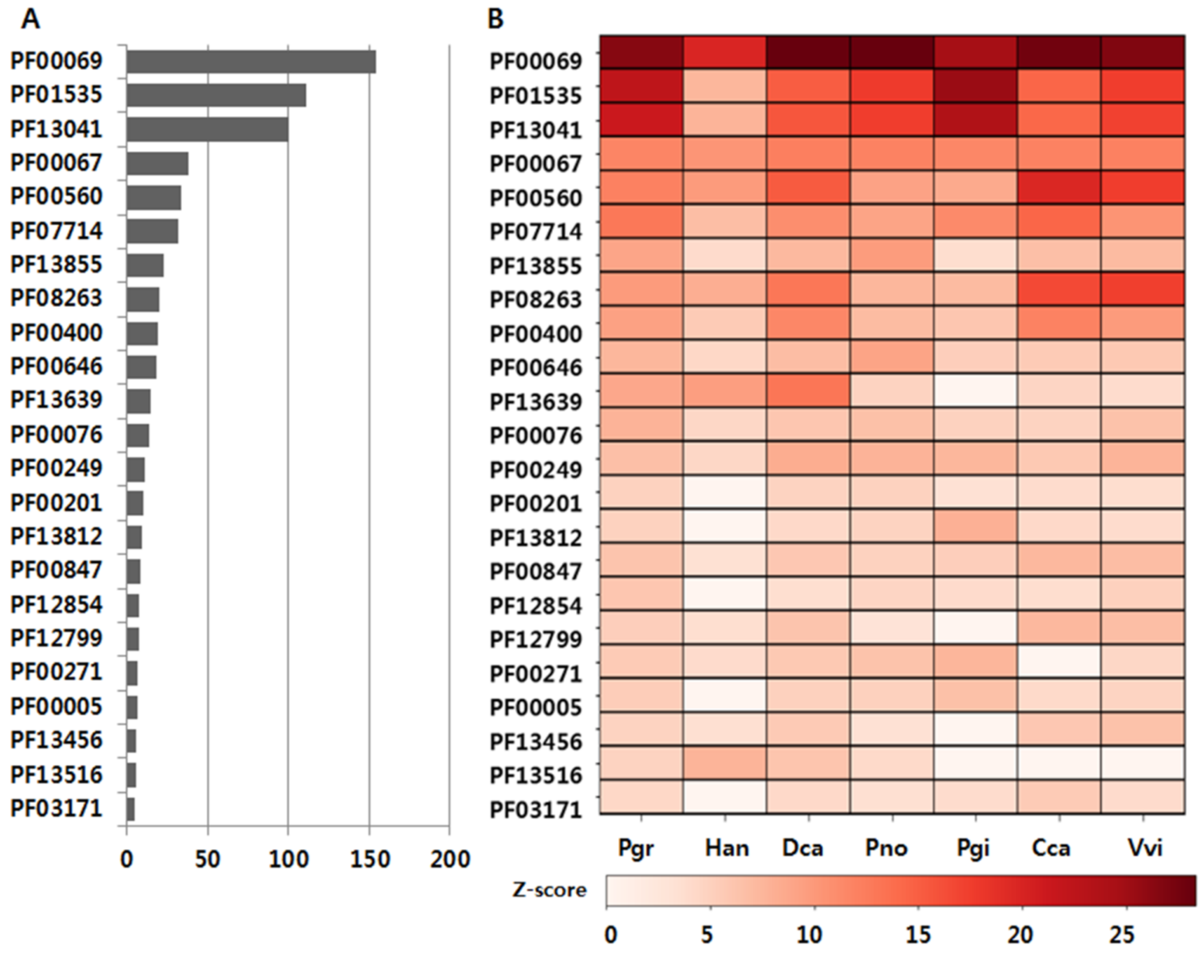

Supplementary Figure S12. Functional domains enriched in the genome of *P. grandiflorus*. **(a)** Functional domains identified by Pfam search and domain enrichments were analyzed by the Z-test (p-value < 0.001). A total of 23 functional domains were enriched in *P. grandiflorus* ($P < 1 \times 10^{-5}$). **(b)** Heatmap showing the relative abundance (Z-score) of functional domains across plant genomes.

<u>Supplementary Note for Supplementary Figure S12</u>:

To compare the abundance of functional domains in related species, the enriched functional

domains were first defined in each plant species with $P < 0.001$ and arranged Z-scores. In each plant species, a null value (0.0) was added for functional domains not meeting the Z-test criteria. Of 23 functional domains, 13 functional domains enriched in *P. grandiflorus* were also enriched in six other plant species analyzed in this study. Four highly ranked gene families, including ATPase (PF00069), ABC transporter (PF01535), cytochrome P450 (PF13041), and protein kinase (PF07714), were expanded in all six plant species. On the other hand, 10 functional domains showed selective expansion. These different levels of gene expansion in evolutionarily related plant species may affect the ability of a plant to synthesize different secondary metabolites[10]. ID and functional description are provided as follows; PF00069, protein kinase domain; PF01535, PPR repeat; PF13041, PPR repeat family; PF07714, protein tyrosine kinase; PF00560, leucine-rich repeat; PF00067, cytochrome P450; PF00400, WD domain; PF00400, G-beta repeat; PF13855, leucine-rich repeat; PF08263, leucine-rich repeat N-terminal domain; PF00076, RNA recognition motif; PF00646, F-box domain; PF13639, ring finger domain; PF00249, Myb-like DNA-binding domain; PF00271, helicase conserved C-terminal domain; PF00005, ABC transporter; PF00201, UDP-glucoronosyl and UDP-glucosyl transferase; PF13812, pentatricopeptide repeat domain; PF12799, PF13516, leucine-rich repeats; PF00847, AP2 domain; PF12854, PPR repeat; PF13456, reverse transcriptase-like; PF03171, 2OG-Fe(II) oxygenase superfamily.

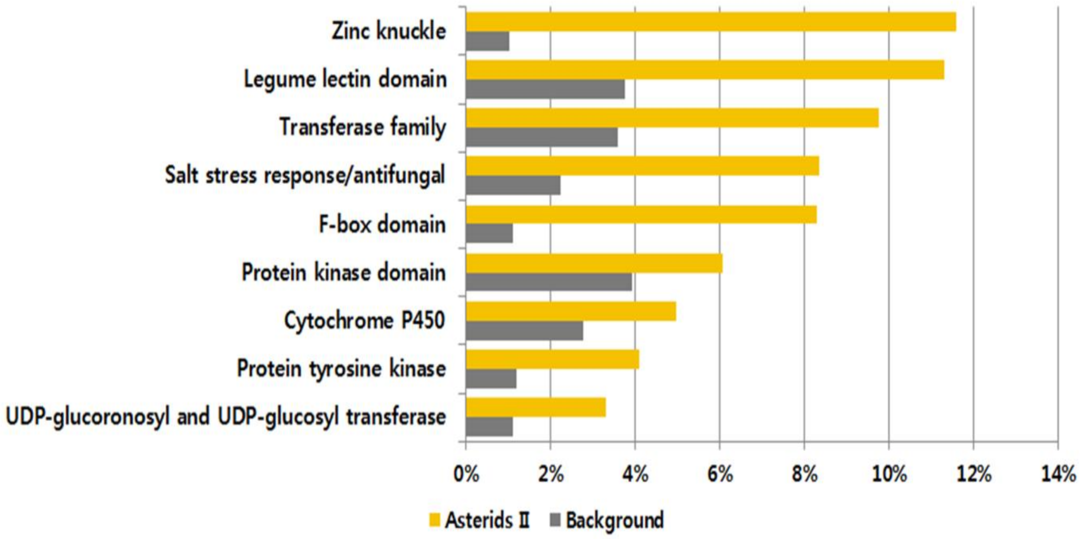

Supplementary Figure S13. Functional enrichment of genes that underwent expansion in the common ancestor of Asterid II species. The expanded gene families were identified using the CAFÉ program (p-value < 0.05). Domains were defined by Pfam analysis, and functional enrichments were analyzed by the modified Fisher's exact test (adjusted p-value < 0.001).

Supplementary Note for Supplementary Figure S13:

Interestingly, this result contained two gene families modifying triterpenoid saponins: UDP-glucoronosyl and UDP-glucosyl transferase (UGT, PF00201) (adjusted p-value = 2.3×10^{-3}) and CYP450 (adjusted p-value = 6.5×10^{-3}). This analysis also showed recursive gene expansion of the *CYP450* family during evolution by showing *CYPP450* expansion in each of the most recent common ancestors MRCA; 5.1×10^{-12} for (Pgi & Pno) and Dca, and 3.6×10^{-11} for Pgi and Pno. In addition, we also observed species-specific expansion of the *CYP450* family in *P. grandiflorus* (1.2×10^{-9}), *H. annuus* (2.8×10^{-5}), *D. carota* (4.0×10^{-3}), *P.*

notoginseng (3.6 x 10^{-3}), *P. ginseng* (5.8 x 10^{-8}), *C. canephora* (2.0 x 10^{-10}), and *V. vinifera* (1.2 x 10^{-10}). We further investigated the presence of a specifically expanded *CYP450* family in each divergent clade.

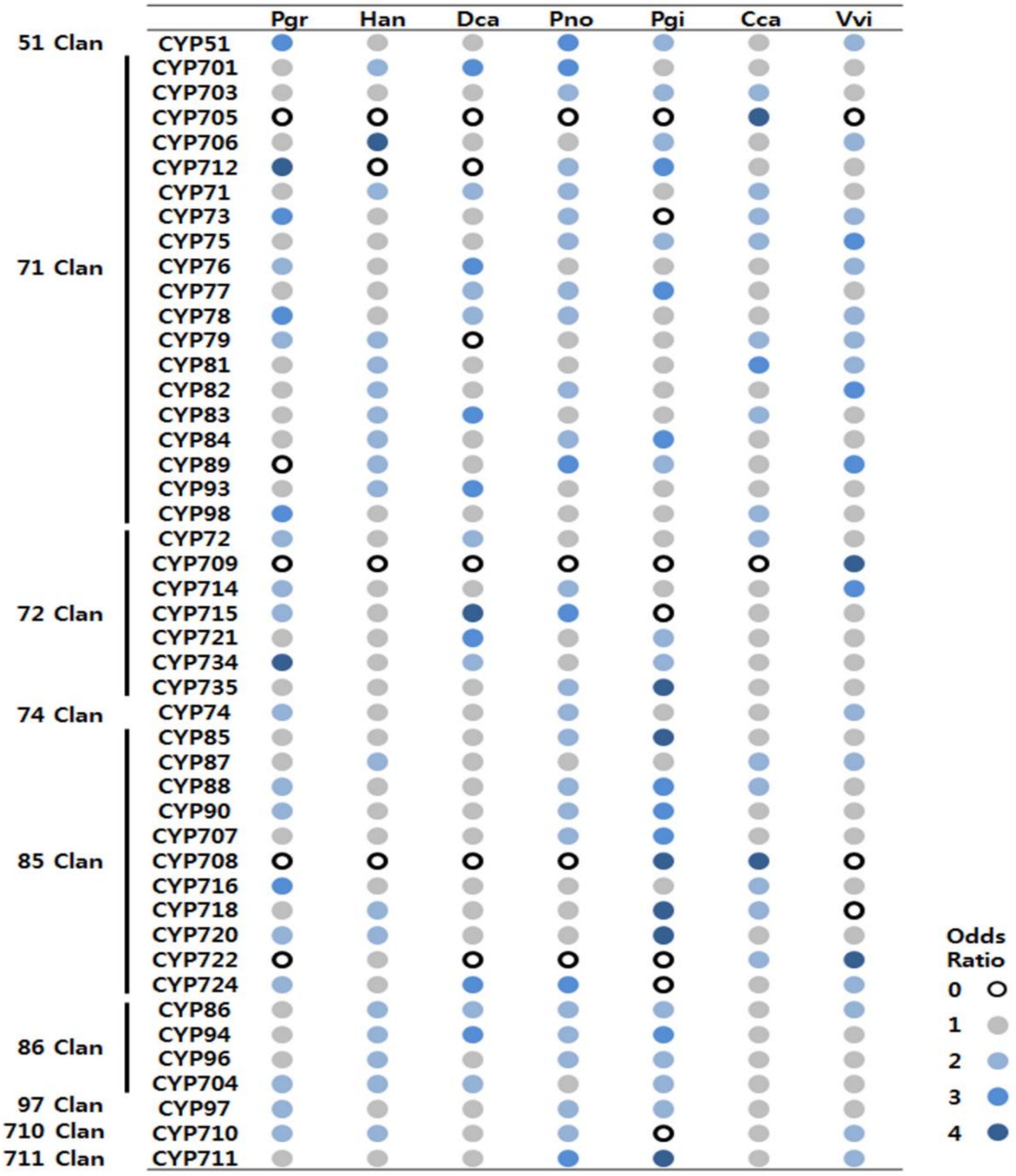

Supplementary Figure S14. Distribution of CYP450 family. Each dot represents the relative abundance of the *CYP450* family and subfamily, as calculated by the odds ratio. Empty circle indicates the absence of *CYP450* genes in that species.

Supplementary Note for Supplementary Figure S14:

To perform a detailed classification of *CYP450* genes, we downloaded the *Arabidopsis* CYP450 supergene family from the cytochrome P450 homepage (http://drnelson.uthsc.edu/CytochromeP450.html)[11], and manually added 62 CYP450 families previously known to synthesize TSs[12,13]. The homology search aligned 2,633 CYP450s against the above CYP450 families by implementing BLASTP, and the classification criterion was followed by the top matched CYP450s with identity ≥ 40%, as suggested by the CYP450 Nomenclature Committee (David Nelson: dnelson@uthsc.edu). A total of 2,337 genes (88.76%) were assigned into the nine CYP450 clans, including 46 subfamilies (Supplementary Table S7); *P. grandiflorus* (311, 83.83%), *H. annuus* (480, 92.13%), *D. carota* (355, 90.10%), *P. notoginseng* (194, 83.98%), *P. ginseng* (296, 84.81%), *C. canephora* (393, 86.56%), and *V. vinifera* (316, 95.18%). Five CYP450 clans (CYP51, CYP74, CYP97, CYP710, and CYP711) were similarly distributed among the seven species examined in this study. Four of these gene families were also observed in gymnosperms, except CYP711[14]. On the other hand, four clans (CYP71, CYP72, CYP85, and CYP86) were highly expanded in angiosperms. These gene families also showed dynamic patterns in this study. We observed that expansions of a particular CYP450 families occurred in the common ancestor of each divergent lineage (Supplementary Table S8). The CYP71B (OG00080) and CYP71A (OG00129) subfamilies were expanded in the MRCA of Asterids, while four CYP450 families, including CYP76C (OG00152 and OG00538), CYP72A (OG00305), CYP716A (OG00114), were expanded in the MRCA of Asterids II. The *P. grandiflorus* genome showed a statistically significant over-representation of *CYP716* (P = 2.0×10^{-5}), *CYP712* (P = 4.3×10^{-3}), and *CYP73* (P = $8.6 \text{ x } 10^{-3}$) genes.

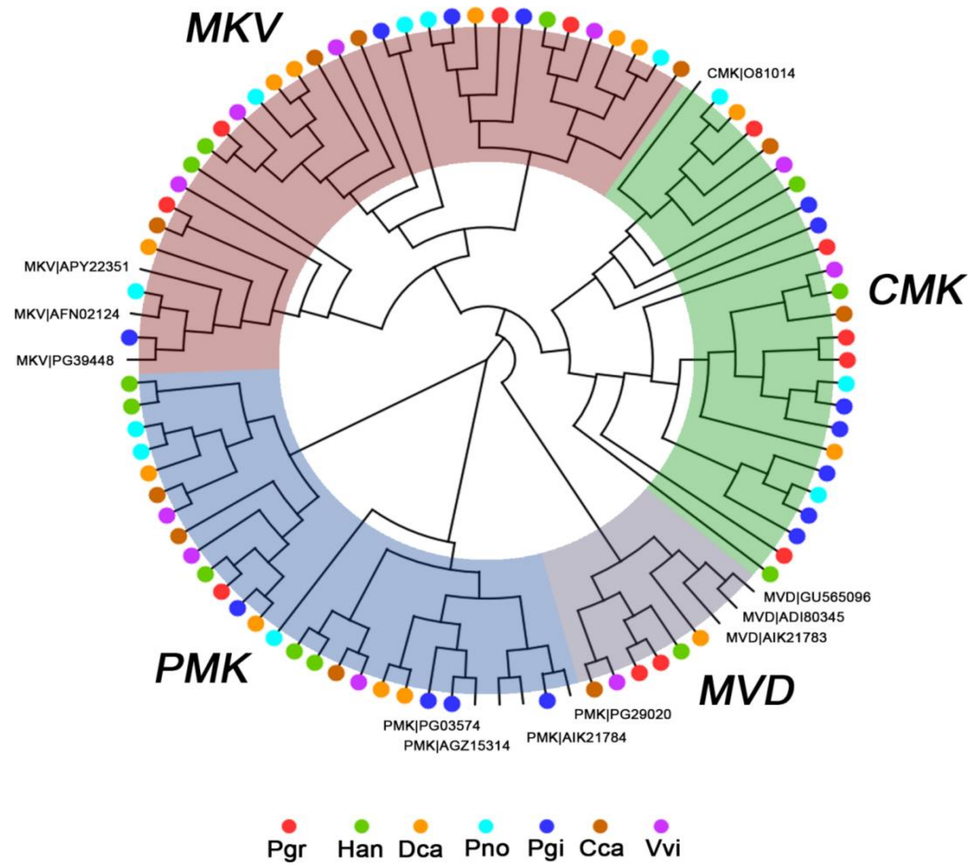

Supplementary Figure S15. Phylogenetic analysis of genes with GHMP kinases. MKV: mevalonate kinase, CMK: cytidylate kinase, MVD: diphosphomevalonate decarboxylase, PMK: phosephomevalonate kinase.

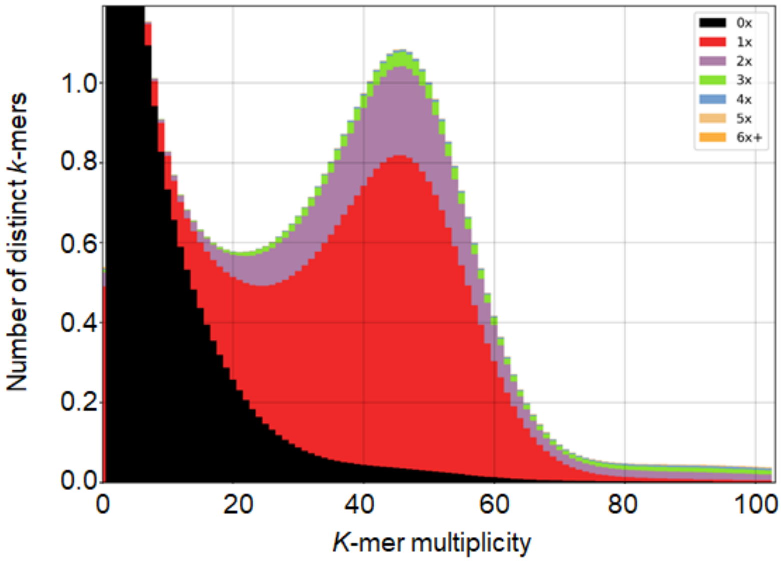

Supplementary Figure S16. *K*-mer spectra copy number plot for checking assembly completeness and heterozygous content. Different color on the stacked bars represents copy number on the assembly. Frequency counts (spectral distribution) are computed on the Illumina paired-end reads.

SUPPLEMENTARY METHODS

Identification of TSB-related genes in *P. grandiflorus*

Besides the *CYP450* family, genes related to TSB via the mevalonic acid (MVA) and methylerythritol 4-phosphate (MEP) pathways, 2,3-oxidosqualene, oxidosqualene cyclases (OSCs), and UGTs were manually selected from the NCBI database, based on literature review and curation (**Supplementary Table S11**). Moreover, several genes were also borrowed from the reference genomes of *Panax ginseng*[15] and *Arabidopsis thaliana*[16]. The functional domains of TSB-related genes were first analyzed (**Supplementary Table S12**). At least one domain was identified by the Pfam search, except for the *CAS* gene, and was used to identify TSB-related genes. For the *CAS* gene, PANTHER (PTHR11764), Gene3D (G3DSA:1.50.10.20), and SUPERFAMILY (SSF48239) databases were employed.

To identify the TSB-related genes in *P. grandiflorus* and six other related species (listed in **Supplementary Table S6**), InterProScan was implemented, and coding domains of genes with the same descriptions were parsed (listed on **Supplementary Table S12**). To perform detailed classification of TSB-related genes with the same domains (e.g., MVK, PMK, MVD), a phylogenetic tree was constructed with predicted genes and collected references (**Supplementary Fig. S15**). Subsequently, TSB-related genes were further classified based on tree topologies (**Fig. 2a and 2b; Supplementary Fig. S14**). Finally, based on domain-based and phylogenetic analyses, 827 putative TSB-related genes involved in the MVA and MEP pathways were identified (**Supplementary Table S13**). Additionally, 1,465 UGTs were identified using the same methods.

Validation of *GGPS* expression in *P. grandiflorus* using qRT-PCR

Total RNA was extracted from the leaves, stems, roots, and flowers of *P. grandiflorus,* and cDNA was synthesized from 1 μg of total RNA using the SuperScript III First-Strand Synthesis System for RT-PCR (Invitrogen). Then, quantitative real-time PCR (qRT-PCR) was performed on Light Cycler 480 II (Roche) in a 20-μl reaction mixture, containing 1 μl of cDNA (10-fold dilution), 10 μl of TOPreal qPCR 2X PreMIX (SYBR Green with low ROX, Enzynomics), and 0.5 μl (10 pmol) of sequence-specific primer (**Supplementary Table S15**), under the following conditions: initial denaturation at 95℃ for 30 s, followed by 40 cycles of denaturation at 95℃ for 5 s and annealing/extension at 60℃ for 30 s. The *Actin* gene of *P. grandiflorus* was used as an internal reference for data normalization. The expression level of genes was then calculated using the ΔCT method.

REFERENCES

1 Chen, N. Using RepeatMasker to identify repetitive elements in genomic sequences. *Current Protocols in Bioinformatics*. **Chapter 4**, Unit 4 10 (2004).

2 Jo, I. H. et al. Isoform sequencing provides a more comprehensive view of the *Panax ginseng* transcriptome. *Genes (Basel)*. **8**, 228 (2017).

3 Wang, B. et al. Unveiling the complexity of the maize transcriptome by single-molecule long-read sequencing. *Nat Commun*. **7**, 11708 (2016).

4 Li, L., Stoeckert, C. J., Jr. & Roos, D. S. OrthoMCL: identification of ortholog groups for eukaryotic genomes. *Genome Res*. **13**, 2178-2189 (2003).

5 Wendel, J. F., Jackson, S. A., Meyers, B. C. & Wing, R. A. Evolution of plant genome architecture. *Genome Biol*. **17**, 37 (2016).

6 Altschul, S. F., Gish, W., Miller, W., Myers, E. W. & Lipman, D. J. Basic local alignment search tool. *Journal of Molecular Biology*. **215**, 403-410 (1990).

7 Guo, A. Y. et al. PlantTFDB: a comprehensive plant transcription factor database. *Nucleic Acids Res*. **36**, D966-969 (2008).

8 Zhang, X. et al. Genome-wide characterisation and analysis of bHLH transcription factors related to tanshinone biosynthesis in *Salvia miltiorrhiza*. *Sci Rep*. **5**, 11244 (2015).

9 Chu, Y. et al. Genome-wide characterization and analysis of bHLH transcription factors in *Panax ginseng*. *Acta Pharm Sin B*. **8**, 666-677 (2018).

10 Chae, L., Kim, T., Nilo-Poyanco, R. & Rhee, S. Y. Genomic signatures of specialized metabolism in plants. *Science*. **344**, 510-513 (2014).

11 Nelson, D. R. The cytochrome p450 homepage. *Human genomics*. **4**, 59 (2009).

12 Miettinen, K. et al. The ancient CYP716 family is a major contributor to the diversification of eudicot triterpenoid biosynthesis. *Nat Commun*. **8**, 14153 (2017).

13 Ghosh, S. Triterpene structural diversification by plant cytochrome P450 enzymes. *Front Plant Sci*. **8**, 1886 (2017).

14 Nelson, D. R., Schuler, M. A., Paquette, S. M., Werck-Reichhart, D. & Bak, S. Comparative genomics of rice and Arabidopsis. Analysis of 727 cytochrome P450 genes and pseudogenes from a monocot and a dicot. *Plant Physiol*. **135**, 756-772 (2004).

15 Xu, J. et al. *Panax ginseng* genome examination for ginsenoside biosynthesis. *Gigascience*. **6**, 1-15 (2017).

16 Pulido, P., Perello, C. & Rodriguez-Concepcion, M. New insights into plant isoprenoid metabolism. *Mol Plant*. **5**, 964-967 (2012).

SUPPLEMENTARY TABLE

Supplementary Table S1. Summary of whole-genome sequencing of *Platycodon grandiflorus*.

Supplementary Table S2. Summary of RNA-Seq of different tissues and methyl jasmonate (MeJA or MJ) treatment of *P. grandiflorus*.

Supplementary Table S3. Summary of whole-genome *de novo* assembly of *P. grandiflorus*.

Supplementary Table S4. Assessment of the genome assembly of *P. grandiflorus* using Benchmarking Universal Single-Copy Orthologs (BUSCOs).

Supplementary Table S5. *Triterpene saponin biosynthesis activation regulator 1* (*TSAR1*) and *TSAR2* homologs in *P. grandiflorus*, and their expression levels in various tissues.

Supplementary Table S6. Plant genomes used for comparative analysis.

Supplementary Table S7. Statistics of *CYP450* clans.

Supplementary Table S8. *CYP450* family expansion and contraction patterns in orthologous groups.

Supplementary Table S9. CYP450s modifying triterpene scaffold.

Supplementary Table S10. Expression of *CYP450* genes in *P. grandiflorus*.

Supplementary Table S11. Reference genes involved in triterpenoid biosynthesis.

Supplementary Table S12. Functional domains of reference genes involved in triterpenoid biosynthesis.

Supplementary Table S13. Statistics of triterpenoid biosynthesis genes.

Supplementary Table S14. Expression profile of triterpenoid saponin biosynthesis (TSB)-related genes classified by phylogenetic analysis.

Supplementary Table S15. qRT-PCR validation for GGPS pralogs in *P. grandiflorus*

Supplementary Table S16. Statistics of whole-genome bisulfite sequencing (WGBS) data after quality control.

Supplementary Table S17. The information of BUSCOs analyzed from the draft genome assembly of *P. grandiflorus*.

Supplementary Table S18. Sequence similarity between duplicated gene fractions in scaffolds

Supplementary Table S19. The information of BUSCOs analyzed from geneset of *P. grandiflorus*.

Supplementary Table S20. Summary of re-mapped short reads to the draft genome assembly of *P. grandiflorus*.
