# Supplementary material for: Whole-genome, transcriptome, and methylome analyses provide insights into the evolution of platycoside biosynthesis in Platycodon grandiflorus, a medicinal plant
Source: Hortic Res. 2020 Jul 1;7:112. doi: 10.1038/s41438-020-0329-x (PMC7327020; doi:10.1038/s41438-020-0329-x)
Supplement: Supplementary file 2 — Supplementary Table [file 41438_2020_329_MOESM2_ESM.pdf]

SUPPLEMENTARY TABLES

Supplementary Table S1. Summary of whole-genome sequencing of *P. grandiflorus.*

Type	Insert size of library	Replicate	No. of reads	Total bases
Short-inserts	270 bp	1	138,425,472	20,902,246,272
		2	120,644,006	18,217,244,906
	500 bp	1	125,708,804	18,982,029,404
		2	146,499,570	22,121,435,070
	700 bp	1	116,565,076	17,601,326,476
		2	146,404,816	22,107,127,216
	360 bp	1	120,641,432	12,064,143,200
Long-mate pairs	2 kb	1	214,248,716	21,639,120,316
		2	202,340,020	20,436,342,020
		3	194,476,778	19,642,154,578
	5 kb	1	216,992,900	21,916,282,900
		2	253,474,850	25,600,959,850
		3	244,885,112	24,733,396,312
	10 kb	1	206,012,446	20,807,257,046
		2	197,558,902	19,953,449,102
		3	186,294,114	18,815,705,514
TSLR long reads	6,791 bp (±757)	1	103,114	394,733,594
		2	102,665	390,689,952
		3	103,067	377,928,304
		4	102,338	394,997,796
		5	82,502	329,216,185
		6	80,160	324,243,217
		7	83,397	332,509,301
		8	78,927	318,839,689
		9	297,686	953,672,389
		10	52,453	168,207,895

Supplementary Table S2. Summary of RNA-Seq of different tissues and methyl jasmonate (MeJA or MJ) treatment of *P. grandiflorus* .

RNA-Seq	Replicate	No. of raw reads	No. of clean reads	No. of mapped reads	No. of uniquely mapped reads
For tissues					
Leaf	Sample pooling	74,059,600	70,914,712	65,255,625	55,702,838
Root	Sample pooling	78,159,428	75,022,212	67,862,786	58,197,998
Stem	Sample pooling	68,667,512	65,879,826	60,264,458	51,708,695
Seed	Sample pooling	66,959,732	64,487,782	59,337,887	50,164,444
Petal	Sample pooling	75,716,052	72,632,398	67,707,666	57,994,330
Pistil	Sample pooling	79,979,182	76,700,112	71,641,213	61,379,959
Sepal	Sample pooling	67,752,160	64,927,762	60,667,487	51,885,721
Stamen	Sample pooling	75,430,728	71,935,884	67,162,525	57,420,997
For MeJA treatment					
Control (CT),<br>(12 h with no exposure to MeJA)	Triplicates	44,007,188	43,714,664	38,636,124	33,432,602
		48,730,380	48,269,348	44,986,099	38,926,377
		49,867,912	49,394,620	44,786,518	38,707,373
MeJA 12 h (MJ 12h),<br>(12 h after exposure to MeJA)	Triplicates	46,104,444	45,820,634	41,008,653	34,876,301
		46,309,014	45,799,192	42,999,494	37,155,964
		48,184,858	47,691,866	44,606,050	38,132,129
MeJA 24 h (MJ 24h),<br>(24 h after exposure to MeJA)	Triplicates	43,679,756	43,249,182	40,068,198	34,105,651
		51,252,656	50,800,366	46,756,153	40,388,543
		46,342,102	45,975,026	41,580,201	35,764,193
MeJA 48 h (MJ 48h),<br>(48 h after exposure to MeJA)	Triplicates	43,046,854	42,761,134	38,719,519	32,604,536
		57,232,018	56,598,656	52,845,623	45,775,973
		48,991,134	48,532,228	45,196,758	39,176,609

Supplementary Table S3. Summary of whole-genome *de novo* assembly of *P. grandiflorus.*

	No. of sequences	Total bases	Longest	N50	N90
Short-read assembly					
Scaffolds [SOAPdenovo2]*	18,379	633,889,740	2,393,941	363,828	43,033
Long-read assembly					
Contigs [Celera Assembler]	68,092	633,447,576	738,183	15,925	5,073
Merged assembly					
Contigs [GARM]	20,039	669,231,464	982,226	55,589	17,875
Gap-filled scaffolds	4,813	697,012,823	1,387,349	272,118	76,041
SSPACE and GapFiller					
Filtration of overlap [Mummer]	4,815	680,148,131	1,387,349	277,181	79,197

*Softeware used for whole-genome *de novo* assembly

Supplementary Table S4. Assessment of the genome assembly of *P. grandiflorus* using Benchmarking Universal Single-Copy Orthologs (BUSCO)

Type	Genome		Gene set			
	Orthologous gene groups		Orthologous gene groups DNA sequence		Orthologous gene groups Protein sequence	
	(No.)	(%)	(No.)	(%)	(No.)	(%)
Complete BUSCOs	412	96.9	384	90.4	386	90.8
Complete and single-copy BUSCOs	361	84.9	354	83.3	350	82.4
Complete and duplicated BUSCOs	51	12.0	30	7.1	36	8.5
Fragmented BUSCOs	10	2.4	33	7.8	35	8.2
Missing BUSCOs	3	0.7	8	1.9	4	0.9

Supplementary Table S5. Triterpene saponin biosynthesis activation regulator 1 (TSAR1) and TSAR2 homologs in *P. grandiflorus* , and their expression levels in various tissues.

Gene ID	Description	BLASTP search		Gene expression in various tissues (FPKM)							
		Bit score	*E* -value	Root	Leaf	Stem	Seed	Pistil	Sepal	Stamen	Petal
PGJG366410	Medtr7g080780.1| TSAR1	125	5.00E-12	0	0	0	0.12	33	1.26	0.35	0
PGJG037830	Medtr4g066460.1| TSAR2	123	3.00E-11	0	0	0	0	0	0	0	0
PGJG172350	Medtr4g066460.1| TSAR2	193	6.00E-22	64.71	7.93	78.11	0	47.34	44.76	0	24.38
PGJG402060	Medtr4g066460.1| TSAR2	122	3.00E-11	0	0	0	0	0	0	0	0

Supplementary Table S6. Plant genomes used for comparative analysis.

Suborder	Species	Abbr.	Genome size (Mb)	No. of proteins	References
Outgroup	*Vitis vinifera*	Vvi	486	25,370	39
Asteride I	*Coffea canephora*	Cca	204	25,574	4
Asteride II	*Daucus carota*	Dca	421	32,490	17
	Panax notoginseng	Pno	1,850	34,369	5
	Panax ginseng	Pgi	3,400	24,678	12
	Helianthus annuus L.	Han	3,000	58,277	18
	Platicodon grandiflorus	Pgr	680.1	40,017	This study

Supplementary Table S7. Statistics of CYP450 clans.

No. of CYP450 class		Pgr	Han	Dca	Pno	Pgi	Cca	Vvi
Other CYP450s		59	41	39	37	53	61	6
CYP51	CYP51	3	1	1	2	2	1	2
CYP71	CYP71	31	76	50	29	32	60	19
	CYP73	12	8	7	4	7	8	6
	CYP75	5	6	8	6	9	16	20
	CYP76	36	34	50	13	13	24	28
	CYP77	2	3	5	2	5	2	2
	CYP78	13	8	8	5	6	4	7
	CYP79	12	16	0	3	6	13	13
	CYP81	15	37	10	9	7	51	24
	CYP82	9	33	11	10	9	13	35
	CYP83	3	17	18	4	6	11	6
	CYP84	2	8	3	5	9	6	5
	CYP89	0	11	4	8	9	2	14
	CYP93	2	9	10	1	2	3	4
	CYP98	4	2	2	1	2	4	1
	CYP701	1	5	6	3	1	2	1
	CYP703	1	1	1	1	2	2	1
	CYP705	0	0	0	0	0	3	0
	CYP706	1	26	7	4	9	3	9
	CYP712	8	0	0	2	6	3	2
CYP72	CYP72	33	43	36	12	19	53	20
	CYP709	0	0	0	0	0	0	1
	CYP714	9	2	11	6	3	6	10
	CYP721	2	4	11	2	7	3	4
CYP74	CYP74	8	6	3	4	4	5	7
CYP85	CYP85	2	2	3	3	6	2	2
	CYP87	3	11	6	3	6	11	10
	CYP88	5	1	2	4	7	7	2
	CYP90	6	6	4	6	10	4	4
	CYP707	4	9	7	5	14	7	5
	CYP708	0	0	0	0	1	2	0
	CYP716	35	24	15	5	12	26	15
	CYP718	1	2	1	0	3	2	0
	CYP720	2	1	1	1	5	2	1
	CYP722	0	1	0	0	0	1	2
	CYP724	2	1	4	2	0	2	2
CYP86	CYP86	5	9	7	5	8	4	6
	CYP94	10	18	10	7	19	9	8
	CYP96	3	16	18	7	12	6	6
	CYP704	9	16	10	5	10	5	7
CYP97	CYP97	2	3	3	2	4	3	2
CYP710	CYP710	1	2	1	1	0	1	1
CYP711	CYP711	1	2	1	2	4	1	2

[1] Pink-color shadow indicates P450 expansions in the corresponding genome

[2] Pgr: *Platicodon grandiflorus* ; Hna: *Helianthus annuus* L. Dca: *Daucus carota* ; Pno: *Panax notoginseng* ; Pgi: *Panax ginseng* ; Cca: *Coffea canephora* ; Vvi: *Vitis vinifera*

Supplementary Table S8. CYP450 family expansion and contraction patterns in orthologous groups.

OG	No. of genes in orthologous group (OG)							Sum	% of genes per total gene No. in specific OG	Major CYP450			Expansion							Contraction						
	Pgr	Han	Dca	Pno	Pgi	Cca	Vvi			Family	No.	%	Pgr	Han	Dca	Pno	Pgi	Cca	Vvi	Pgr	Han	Dca	Pno	Pgi	Cca	Vvi
OG00053	0	0	0	0	0	1	0	1	0.6%	CYP72A	1	100.00%														
OG00059	19	38	28	10	11	35	19	160	100.0%	CYP72A	160	100.00%		O	O			O		O			O			O
OG00080	13	11	15	17	14	33	7	110	91.7%	CYP71B	75	68.18%						O		O	O			O		O
OG00100	13	31	9	6	4	16	22	101	100.0%	CYP81	100	99.01%		O					O	O		O		O		
OG00102	7	28	11	7	8	11	28	100	100.0%	CYP82C	96	96.00%		O					O			O	O		O	
OG00114	23	24	14	5	7	7	11	91	100.0%	CYP716A	91	100.00%	O	O	O								O		O	O
OG00129	7	37	18	3	1	14	2	82	100.0%	CYP71A	82	100.00%		O	O			O		O				O		O
OG00137	6	2	20	17	21	8	6	80	100.0%	others	39	48.75%			O		O			O	O		O			O
OG00152	19	20	19	2	0	6	10	76	100.0%	CYP76C	76	100.00%	O	O	O									O	O	O
OG00222	1	15	18	6	9	5	5	59	100.0%	CYP96A	59	100.00%		O	O		O			O			O		O	O
OG00236	3	14	18	4	4	8	6	57	100.0%	CYP83B	56	98.25%		O	O					O						O
OG00271	1	26	7	3	4	3	6	50	98.0%	CYP706A	49	98.00%		O	O					O			O		O	
OG00305	15	6	3	5	10	4	0	43	93.5%	CYP72A	27	62.79%	O									O	O		O	O
OG00331	5	13	8	4	5	3	6	44	100.0%	CYP704A	44	100.00%		O	O					O			O		O	
OG00335	8	13	0	2	4	10	7	44	100.0%	CYP79A	44	100.00%	O	O			O	O				O	O			
OG00342	3	9	7	5	9	4	5	42	97.7%	CYP707	42	100.00%		O	O		O			O			O		O	O
OG00405	6	2	18	2	2	2	6	38	100.0%	CYP76G	33	86.84%	O		O				O		O				O	
OG00458	6	1	9	3	3	5	8	35	100.0%	CYP714A	27	77.14%	O		O				O		O					
OG00471	1	9	3	2	2	8	9	34	100.0%	CYP87A	30	88.24%		O				O	O	O		O				
OG00496	0	10	4	2	4	1	12	33	100.0%	CYP89A	33	100.00%		O			O		O	O			O		O	
OG00530	5	15	4	0	0	0	7	31	100.0%	CYP71B	29	93.55%		O					O						O	
OG00532	3	7	5	3	5	3	5	31	100.0%	CYP78A	31	100.00%		O	O		O		O	O			O		O	
OG00538	0	8	11	5	7	0	0	31	100.0%	CYP76C	25	80.65%		O	O		O			O			O		O	O
OG00619	2	2	4	1	2	14	4	29	100.0%	CYP75B	23	79.31%			O			O		O	O		O			
OG00623	2	3	1	2	3	4	14	29	100.0%	CYP75B	29	100.00%							O	O		O	O			
OG00671	1	8	9	1	2	2	4	27	100.0%	CYP93	27	100.00%		O	O					O			O		O	
OG00752	2	1	2	4	7	6	2	24	96.0%	CYP88A	24	100.00%			O		O				O		O		O	O
OG00819	1	4	6	2	4	3	4	24	100.0%	CYP721A	24	100.00%		O	O		O		O	O			O			
OG00879	5	3	1	2	2	4	6	23	100.0%	CYP74A	23	100.00%														
OG00908	3	4	4	3	2	3	3	22	100.0%	CYP86A	22	100.00%		O	O					O			O	O		
OG00948	1	3	6	4	3	3	2	22	100.0%	CYP94B	16	72.73%			O	O				O						O
OG00982	1	7	3	1	4	2	3	21	100.0%	CYP84A	21	100.00%		O			O			O			O		O	
OG01053	2	2	3	3	6	2	2	20	100.0%	CYP85A	20	100.00%					O			O	O		O		O	O
OG01252	1	0	0	0	0	0	0	1	5.3%	CYP72A	1	100.00%	O								O	O			O	O
OG01264	1	5	6	2	1	2	1	18	100.0%	CYP701A	18	100.00%		O	O					O				O	O	O

OG01275	2	5	3	1	3	1	3	18	100.0%	CYP86B	18	100.00%		O			O			O			O		O	
OG01353	3	3	2	2	3	2	3	18	100.0%	CYP94C	18	100.00%										O	O		O	
OG01590	3	3	2	2	0	3	3	16	100.0%	CYP73A	16	100.00%										O		O		
OG01664	4	6	1	0	1	0	4	16	100.0%	others	13	81.25%	O	O					O			O	O		O	
OG01883	6	1	3	1	1	1	2	15	100.0%	CYP78A	15	100.00%	O		O						O				O	
OG01922	4	3	2	1	1	2	2	15	100.0%	CYP90	15	100.00%														
OG01978	2	3	2	2	2	1	2	14	100.0%	CYP94B	14	100.00%														
OG02133	1	3	3	1	2	2	2	14	100.0%	CYP734A	14	100.00%		O	O					O			O			
OG02197	3	1	1	2	2	1	2	12	85.7%	CYP51G	12	100.00%														
OG02202	0	8	0	0	0	2	4	14	100.0%	others	11	78.57%		O					O	O						
OG02628	1	0	1	1	0	1	0	4	33.3%	CYP87D	3	75.00%														
OG02813	1	2	2	1	4	1	1	12	100.0%	CYP77A	12	100.00%					O			O			O		O	O
OG02886	2	1	4	1	0	2	2	12	100.0%	CYP724A	12	100.00%			O						O			O		
OG03450	2	1	5	1	0	1	1	11	100.0%	CYP715A	11	100.00%			O						O			O	O	O
OG03521	0	3	2	2	1	1	1	10	90.9%	CYP74B	10	100.00%														
OG03634	4	0	0	1	2	3	1	11	100.0%	CYP712A	11	100.00%	O				O	O			O	O				
OG04156	0	0	7	1	1	0	1	10	100.0%	CYP71	9	90.00%			O					O	O				O	
OG04230	1	2	2	1	1	2	1	10	100.0%	CYP98A	10	100.00%														
OG04639	3	1	0	3	0	0	3	10	100.0%	others	8	80.00%	O			O			O			O		O	O	
OG05125	2	4	1	1	0	1	0	9	100.0%	CYP94D	9	100.00%	O	O								O		O		O
OG05126	1	3	0	1	2	1	1	9	100.0%	CYP94D	9	100.00%		O			O									
OG05383	1	1	1	2	2	1	1	9	100.0%	CYP711	9	100.00%														
OG05441	1	1	1	1	3	1	1	9	100.0%	CYP735A	9	100.00%					O									
OG05789	1	1	1	1	3	1	1	9	100.0%	CYP90B	9	100.00%					O									
OG06994	1	1	1	2	1	1	1	8	100.0%	others	8	100.00%				O										
OG07123	1	2	1	1	1	1	1	8	100.0%	CYP90A	8	100.00%														
OG07670	1	1	1	0	2	3	0	8	100.0%	CYP71A	6	75.00%					O	O					O			O
OG08227	1	1	1	1	1	1	1	7	100.0%	CYP77B	7	100.00%														
OG08411	1	1	1	1	1	1	1	7	100.0%	CYP704B	7	100.00%														
OG08576	1	2	1	1	0	1	1	7	100.0%	CYP710A	7	100.00%		O										O		
OG09141	1	1	1	1	1	1	1	7	100.0%	CYP703A	7	100.00%														
OG09380	1	2	1	0	1	2	0	7	100.0%	CYP718A	7	100.00%		O				O					O			O
OG09798	1	1	1	1	0	2	1	7	100.0%	CYP76G	7	100.00%						O						O		
OG09942	1	1	1	0	2	1	1	7	100.0%	CYP97C	7	100.00%					O						O			
OG10260	1	1	1	1	1	1	1	7	100.0%	CYP720A	7	100.00%														
OG10646	0	1	1	1	2	1	0	6	100.0%	CYP97A3	6	100.00%					O			O						O
OG10795	1	1	1	1	1	1	0	6	100.0%	others	6	100.00%														
OG12219	1	1	1	1	0	1	1	6	100.0%	CYP97B3	6	100.00%												O		
OG12872	0	0	0	0	0	2	4	6	100.0%	CYP76C4	6	100.00%														

OG14041	0	0	0	1	1	3	0	5	100.0%	CYP71B34	4	80.00%						O		O	O	O				O
OG14052	4	0	0	0	0	1	0	5	100.0%	others	5	100.00%														
OG14152	1	1	0	0	0	1	1	4	80.0%	CYP722A	3	75.00%	O								O	O		O		O
OG14183	0	4	0	0	0	1	0	5	100.0%	CYP81	5	100.00%		O						O						O
OG14978	0	0	0	0	0	3	1	4	100.0%	CYP705A	3	75.00%				O										
OG16022	0	0	1	1	1	0	0	3	100.0%	CYP712A	2	66.67%														
OG16078	0	0	1	2	0	0	0	3	100.0%	CYP715A	3	100.00%								O	O			O	O	O
OG16537	1	0	0	0	0	0	2	3	100.0%	CYP76C	3	100.00%				O										
OG16770	0	0	0	2	1	0	0	3	100.0%	others	3	100.00%								O	O	O			O	O
OG16803	1	1	0	0	1	0	0	3	100.0%	CYP72A	2	66.67%										O	O		O	O
OG18118	0	0	1	0	1	0	0	2	100.0%	CYP721A	2	100.00%								O	O		O		O	O
OG18501	0	0	0	0	1	1	0	2	100.0%	CYP71A	2	100.00%								O	O	O	O			O
OG18553	0	1	0	0	0	1	0	2	100.0%	CYP93E	2	100.00%														
OG18699	0	0	0	0	0	1	1	2	100.0%	CYP716C	2	100.00%														
OG18737	0	0	0	0	0	1	1	2	100.0%	CYP71B	2	100.00%														
OG18741	0	0	0	0	0	1	1	2	100.0%	CYP72A	2	100.00%														
OG19236	0	0	0	1	0	0	1	2	100.0%	CYP79A	2	100.00%								O	O	O		O	O	
OG19277	0	0	0	0	1	0	1	2	100.0%	CYP87A	2	100.00%								O	O	O	O		O	
OG19932	0	0	0	1	1	0	0	2	100.0%	CYP706A	2	100.00%								O	O	O			O	O
OG20120	0	0	0	1	1	0	0	2	100.0%	CYP90A	2	100.00%								O	O	O			O	O
OG20121	0	0	0	1	1	0	0	2	100.0%	CYP90A	2	100.00%								O	O	O			O	O
OG20379	0	0	0	1	1	0	0	2	100.0%	CYP71A	1	50.00%								O	O	O			O	O

[1] Blue-color shadow indicates expansion or contraction events at the common ancestry

[2] 'O' indicates species-specific gene expansion and contraction

[3] Pink shadow indicates P450 expansions in current genome

[4] Pgr: *Platicodon grandiflorus* ; Hna: *Helianthus annuus* L. Dca: *Daucus carota* ; Pno: *Panax notoginseng* ; Pgi: *Panax ginseng* ; Cca: *Coffea canephora* ; Vvi:

Supplementary Table S9. CYP450s modifying triterpene scaffold.

	Pgr	Han	Dca	Pno	Pgi	Cca	Vvi	Reaction	Substrate
**CYP716A110**	3	0	1	0	0	0	0	C28 oxidation	β-amyrin
CYP716A12	1	0	0	0	0	0	0	C28 oxidation	β-amyrin/α-amyrin/lupeol
**CYP716A140**	4	0	0	0	0	0	0	C28 oxidation	β-amyrin/α-amyrin
**CYP716A141**	4	5	0	1	0	0	0	C28 oxidation/C16β hydroxylation	β-amyrin/olenolic acid
CYP716A15	2	0	0	0	0	0	3	C28 oxidation	β-amyrin
CYP716A154	0	1	0	0	0	2	0	C28 oxidation	β-amyrin/α-amyrin/lupeol
CYP716A17	0	0	0	0	0	0	1	C28 oxidation	β-amyrin
**CYP716A253**	4	5	0	0	1	2	1	C28 oxidation	β-amyrin/α-amyrin
CYP716A46	0	0	0	0	1	0	0	C28 oxidation	β-amyrin/α-amyrin
CYP716A52v2	0	0	1	1	2	0	0	C28 oxidation	β-amyrin
**CYP716A75**	1	0	0	0	0	0	0	C28 oxidation	β-amyrin
CYP716A83	0	0	0	0	1	0	0	C28 oxidation	β-amyrin/α-amyrin
CYP716A86	0	0	1	1	3	0	0	C28 oxidation	β-amyrin
CYP716C11	0	0	0	0	0	2	1	C2α hydroxylation	oleanolic acid/6β-hydroxy-oleanolic acid/ursolic acid
**CYP716E41**	2	1	11	1	2	9	0	C16β hydroxylation	oleanolic acid/ursolic acid/maslinic acid
**CYP716S5**	4	0	0	0	0	0	0	C12-C13α epoxidation	β-amyrin/oleanolic acid
**CYP716U1**	2	0	0	0	0	5	0	C12 hydroxylation	dammarenediol-II
CYP72A154	5	0	2	1	1	6	1	C30 oxidation	β-amyrin/11-oxo-β-amyrin
CYP72A61	0	0	0	0	0	1	1	C22 hydroxylation	24-hydroxy-βamyrin
CYP72A63	0	1	0	0	0	0	0	C30 oxidation	24-hydroxy-β-amyrin
CYP72A67	1	1	1	1	4	7	6	C2β hydroxylation	oleanolic acid hedragenin
CYP87D16	1	0	1	1	0	2	3	C16α hydroxylation	β-amyrin
CYP93E2	0	0	0	0	0	1	0	C24 hydroxylation	β-amyrin
CYP93E3	1	3	5	1	1	0	3	C24 hydroxylation	β-amyrin
CYP93E8	0	1	1	0	0	0	0	C24 hydroxylation	β-amyrin

* Pgr: *Platicodon grandiflorus* ; Hna: *Helianthus annuus* L. Dca: *Daucus carota* ; Pno: *Panax notoginseng* ; Pgi: *Panax ginseng* ; Cca: *Coffea*

Supplementary Table S10. Expression of CYP450 genes in *P. grandiflorus* .

Gene/CYP subfamily		FPKM in tissues								log_2 (FPKM) in tissues								FPKM in methyl jasmonate (MeJA or MJ) treatment				Log_2 (FPKM) in MeJA treatment			
		Root	Leaf	Stem	Seed	Pistil	Sepal	Stamen	Petal	Root	Leaf	Stem	Seed	Pistil	Sepal	Stamen	Petal	(Average) Control (CT) 12h	(Average) MJ 12h	(Average) MJ 24h	(Average) MJ 48h	(Average) CT 12h	(Average) MJ 12h	(Average) MJ 24h	(Average) MJ 48h
PGJG090860	CYP716A110	0.00	0.00	0.00	0.00	0.00	0.00	0.00	0.00	0.00	0.00	0.00	0.00	0.00	0.00	0.00	0.00	0.00	0.00	0.00	0.00	0.00	0.00	0.00	0.00
PGJG182820	CYP716A110	10.11	0.00	1.54	0.00	0.11	0.31	0.43	0.32	3.34	0.00	0.62	0.00	-3.23	-1.68	-1.22	-1.64	0.32	2.13	2.50	2.34	-1.63	1.09	1.32	1.23
PGJG236440	CYP716A110	0.07	0.00	0.00	0.00	0.00	0.00	0.00	0.00	-3.80	0.00	0.00	0.00	0.00	0.00	0.00	0.00	0.00	0.00	0.00	0.00	0.00	0.00	0.00	0.00
PGJG054290	CYP716A12	0.00	0.00	0.00	0.00	0.00	0.10	0.00	29.41	0.00	0.00	0.00	0.00	0.00	-3.28	0.00	4.88	0.18	0.28	0.11	0.02	-2.50	-1.82	-3.14	-5.42
PGJG086700	CYP716A140	149.55	31.95	201.19	121.42	5.76	86.97	59.32	0.25	7.22	5.00	7.65	6.92	2.53	6.44	5.89	-2.02	29.41	61.99	55.43	108.67	4.88	5.95	5.79	6.76
PGJG086710	CYP716A140	3.41	0.07	1.78	3.71	0.07	1.09	1.51	0.00	1.77	-3.74	0.83	1.89	-3.85	0.13	0.60	0.00	0.26	0.17	0.17	0.30	-1.94	-2.58	-2.58	-1.72
PGJG233930	CYP716A140	36.35	4.34	30.53	16.30	0.64	25.24	24.44	0.14	5.18	2.12	4.93	4.03	-0.65	4.66	4.61	-2.87	5.47	8.79	5.42	5.13	2.45	3.14	2.44	2.36
PGJG266060	CYP716A140	0.77	0.12	0.10	54.39	0.06	0.14	0.17	0.20	-0.37	-3.01	-3.33	5.77	-4.12	-2.83	-2.54	-2.30	0.14	0.23	0.16	0.23	-2.84	-2.10	-2.61	-2.14
PGJG051500	CYP716A141	0.00	0.00	0.00	0.00	0.00	0.00	0.00	0.00	0.00	0.00	0.00	0.00	0.00	0.00	0.00	0.00	0.00	0.00	0.00	0.00	0.00	0.00	0.00	0.00
PGJG224290	CYP716A141	0.68	0.18	0.59	0.00	0.86	1.85	1.64	0.00	-0.56	-2.44	-0.75	0.00	-0.21	0.89	0.72	0.00	0.33	1.21	0.73	1.04	-1.61	0.28	-0.45	0.05
PGJG224310	CYP716A141	1.95	1.54	4.08	0.00	4.07	7.63	3.45	0.05	0.97	0.62	2.03	0.00	2.02	2.93	1.79	-4.31	1.46	3.20	2.03	5.34	0.55	1.68	1.02	2.42
PGJG310130	CYP716A141	143.38	23.72	171.56	157.03	23.01	80.99	47.03	0.16	7.16	4.57	7.42	7.29	4.52	6.34	5.56	-2.62	40.34	68.43	59.75	92.26	5.33	6.10	5.90	6.53
PGJG016220	CYP716A15	0.00	0.00	0.00	0.00	2.58	6.75	9.24	0.00	0.00	0.00	0.00	0.00	1.36	2.76	3.21	0.00	0.75	0.61	0.46	0.24	-0.42	-0.72	-1.12	-2.04
PGJG055350	CYP716A15	0.00	0.00	0.00	0.00	0.00	0.00	0.00	0.00	0.00	0.00	0.00	0.00	0.00	0.00	0.00	0.00	0.00	0.00	0.00	0.00	0.00	0.00	0.00	0.00
PGJG105300	CYP716A253	0.00	0.00	0.00	0.00	0.00	0.11	0.00	0.04	0.00	0.00	0.00	0.00	0.00	-3.222	0.00	-4.803	0.11	0.16	0.00	0.00	-3.18	-2.64	0.00	0.00
PGJG176980	CYP716A253	0.18	0.29	33.70	0.04	2.57	0.00	5.07	18.25	-2.48	-1.80	5.07	-4.56	1.36	0.00	2.34	4.19	0.48	0.37	0.07	0.11	-1.07	-1.43	-3.77	-3.23
PGJG229250	CYP716A253	0.93	0.12	0.04	0.00	0.00	0.11	0.00	4.29	-0.11	-3.05	-4.59	0.00	0.00	-3.25	0.00	2.10	1.08	0.42	0.27	0.12	0.12	-1.24	-1.91	-3.02
PGJG358600	CYP716A253	0.00	0.00	0.00	0.00	0.00	0.08	0.00	0.00	0.00	0.00	0.00	0.00	0.00	-3.73	0.00	0.00	0.14	0.02	0.00	0.00	-2.80	-5.42	0.00	0.00
PGJG229240	CYP716A75	0.24	0.00	0.00	0.00	0.00	0.05	0.06	0.00	-2.04	0.00	0.00	0.00	0.00	-4.38	-4.10	0.00	0.11	0.00	0.11	0.03	-3.14	0.00	-3.23	-5.23
PGJG030760	CYP716E41	0.10	0.00	0.00	0.00	0.00	0.00	0.00	0.00	-3.28	0.00	0.00	0.00	0.00	0.00	0.00	0.00	0.00	0.00	0.00	0.00	0.00	0.00	0.00	0.00
PGJG393930	CYP716E41	0.00	0.00	0.00	0.00	0.00	0.00	0.00	0.00	0.00	0.00	0.00	0.00	0.00	0.00	0.00	0.00	0.00	0.00	0.00	0.00	0.00	0.00	0.00	0.00
PGJG127160	CYP716S5	0.00	0.00	0.00	1.35	0.00	0.00	0.15	0.00	0.00	0.00	0.00	0.43	0.00	0.00	-2.72	0.00	1.63	1.81	1.90	5.94	0.70	0.86	0.93	2.57
PGJG153470	CYP716S5	0.07	31.55	0.21	0.00	38.18	45.85	71.05	0.34	-3.77	4.98	-2.22	0.00	5.25	5.52	6.15	-1.57	130.07	101.78	111.36	108.19	7.02	6.67	6.80	6.76
PGJG182920	CYP716S5	0.14	0.00	0.04	0.00	0.03	0.27	0.04	0.00	-2.85	0.00	-4.65	0.00	-4.85	-1.88	-4.59	0.00	0.00	0.00	0.00	0.00	0.00	0.00	0.00	0.00
PGJG363570	CYP716S5	0.07	0.00	0.00	0.00	0.00	0.25	0.13	0.00	-3.77	0.00	0.00	0.00	0.00	-1.99	-2.92	0.00	0.00	0.00	0.00	0.00	0.00	0.00	0.00	0.00
PGJG339270	CYP716U1	1.00	0.00	0.52	0.00	0.15	0.00	0.72	0.15	0.01	0.00	-0.96	0.00	-2.75	0.00	-0.47	-2.72	0.18	0.48	1.29	2.79	-2.50	-1.05	0.36	1.48
PGJG339280	CYP716U1	0.21	0.00	0.08	0.00	0.04	0.17	0.17	0.51	-2.24	0.00	-3.62	0.00	-4.83	-2.53	-2.57	-0.98	0.28	0.45	0.19	0.07	-1.85	-1.16	-2.40	-3.77
PGJG066000	CYP72A154	0.00	0.00	0.00	0.00	0.00	0.00	0.00	0.00	0.00	0.00	0.00	0.00	0.00	0.00	0.00	0.00	0.05	0.00	0.00	0.00	-4.23	0.00	0.00	0.00
PGJG074210	CYP72A154	0.00	0.00	0.00	0.00	0.00	0.00	0.00	0.00	0.00	0.00	0.00	0.00	0.00	0.00	0.00	0.00	0.02	0.00	0.00	0.02	-5.64	0.00	0.00	-5.64
PGJG174660	CYP72A154	9.72	5.65	12.37	1.15	5.16	15.01	9.43	0.08	3.282	2.500	3.629	0.196	2.366	3.908	3.237	-3.632	7.43	8.02	7.79	10.73	2.89	3.00	2.96	3.42
PGJG275090	CYP72A154	0.32	0.09	0.40	0.00	0.00	0.06	0.03	0.03	-1.654	-3.404	-1.309	0.00	0.00	-4.145	-4.864	-5.050	2.04	2.44	0.98	2.61	1.03	1.29	-0.02	1.38
PGJG362880	CYP72A154	0.02	0.00	0.00	0.00	0.00	0.00	0.00	0.00	-5.626	0.00	0.00	0.00	0.00	0.00	0.00	0.00	0.04	0.01	0.01	0.01	-4.53	-6.64	-6.64	-6.64
PGJG104700	CYP72A67	0.09	0.00	0.00	0.00	0.00	0.00	0.00	0.00	-3.427	0.00	0.00	0.00	0.00	0.00	0.00	0.00	0.21	0.14	0.05	0.11	-2.25	-2.80	-4.32	-3.23
PGJG009420	CYP87D16	0.68	0.44	0.52	8.98	0.30	0.19	0.44	0.04	-0.553	-1.197	-0.948	3.167	-1.722	-2.410	-1.190	-4.548	0.46	0.47	0.30	0.31	-1.12	-1.08	-1.75	-1.69
PGJG080240	CYP93E3	2.17	0.00	0.00	0.00	0.00	0.00	0.00	0.00	1.114	0.00	0.00	0.00	0.00	0.00	0.00	0.00	0.15	0.01	0.00	0.00	-2.74	-6.23	0.00	0.00

Supplementary Table S11. Reference genes involved in triterpenoid biosynthesis.

Pathways	Enzyme	Acronym	Accession
MVA	Acetoacetyl-CoA thiolase [a]	AACT	At5g47720
			At5g48230
	3-hydroxy-3-methylglutaryl CoA synthase [b]	HMGS	PG24351
			AKP55622.1
			AIK21781
			ADI80347.1
	3-hydroxy-3-methylglutaryl CoA reductase [b]	HMGR	PG07131
			PG03840
	Mevalonate kinase [b]	MVK	PG39448
	5-phosphomevalonate kinase [b]	PMK	PG29020
			PG03574
	5-diphosphomevalonate decarboxylase	MVD	GU565096
			ADI80345.1
			AIK21783.1
MEP	1-deoxy-D-xylulose 5-phosphate synthase [a]	DXS	At4g15560
	1-deoxy-D-xylulose 5-phosphate reductoisomerase [a]	DXR	At5g62790
	2-C-methyl-D-erythritol 4-phosphate cytidyltransferase [a]	MCT	At2g02500
	4-(cytidine 5′-diphospho)-2-C-methyl-D-erythritol kinase [a]	CMK	At2g26930
	2-C-methyl-D-erythritol 2,4-cyclodiphosphate synthase [a]	MDS	At1g63970
	1-hydroxy-2-methyl-2-butenyl 4-diphosphate synthase [a]	HDS	At5g60600
	1-hydroxy-2-methyl-2-butenyl 4-diphosphate reductase [a]	HDR	At4g34350
IPP isomerase	Isopentenyl diphosphate isomerase [a]	IDI	At5g16440
			At3g02780
OSC groups *	β-amyrin synthase [b]	bAS	PG00849
			PG15263
			PG02865
	Oleanolic acid synthase [b]	OAS	PG13959
			PG19915
	Dammarenediol synthase [b, d]	DDS	PG36431
			AB122080
	Protopanaxadiol synthase [d]	PPDS	N/A
	Protopanaxatriol synthase [b]	PPTS	PG26599
	Lanosterol synthase [b]	LAS	PG28400
	Cycloartenol synthase [b]	CAS	PG39173
			PG03815

* Oxidosqualen cyclase (OSC)

[a] Pulido P, Perello C, Rodriguez-Concepcion M: **New insights into plant isoprenoid metabolism.** Mol Plant 2012, 5:964-967.

[b] Jiang X, Yang C, Baosheng L, Shuiming X, Qinggang Y, Rui B, He S, Linlin D, Xiwen L, Jun Q, et al: **Panax ginseng genome examination for ginsenoside biosynthesis.** *Gigascience* 2017.

[c] Luo H, Sun C, Sun Y, Wu Q, Li Y, Song J, Niu Y, Cheng X, Xu H, Li C, et al: **Analysis of the transcriptome of Panax notoginseng root uncovers putative triterpene saponin-biosynthetic genes and genetic markers.** *BMC Genomics* 2011, **12**

[d] Han JY, Kwon YS, Yang DC, Jung YR, Choi YE: **Expression and RNA Interference-Induced Silencing of the Dammarenediol Synthase Gene in Panax ginseng.** *Plant and Cell Physiology* 2006, **47:**1653-1662.

Supplementary Table S12. Functional domains of reference genes involved in triterpenoid biosynthesis.

Acronym	Genes	Pfam ID	Pfam Domains
AACT	At5g47720	PF02803	Thiolase, C-terminal domain
		PF00108	Thiolase, N-terminal domain
HMGS	ADI80347.1	PF01154	Hydroxymethylglutaryl-coenzyme A synthase N terminal
		PF08540	Hydroxymethylglutaryl-coenzyme A synthase C terminal
HMGR	ACV65036.1	PF00368	Hydroxymethylglutaryl-coenzyme A reductase
MVK[a]	PG39448	PF00288	GHMP kinases N terminal domain
PMK[a]	AIK21784.1	PF00288	GHMP kinases N terminal domain
		PF08544	GHMP kinases C terminal
MVD[a]	ADI80345.1	PF00288	GHMP kinases N terminal domain
DXS	NP_193291.1	PF13292	1-deoxy-D-xylulose-5-phosphate synthase
		PF02780	Transketolase, C-terminal domain
		PF02779	Transketolase, pyrimidine binding domain
DXR	NP_201085.1	PF13288	DXP reductoisomerase C-terminal domain
		PF08436	1-deoxy-D-xylulose 5-phosphate reductoisomerase C-terminal
		PF02670	1-deoxy-D-xylulose 5-phosphate reductoisomerase
MCT	P69834.1	PF01128	2-C-methyl-D-erythritol 4-phosphate cytidylyltransferase
CMK[a]	O81014.1	PF08544	GHMP kinases C terminal
		PF00288	GHMP kinases N terminal domain
MDS	Q9CAK8.1	PF02542	YgbB family
HDS	F4K0E8.1	PF04551	GcpE protein
HDR	Q94B35.1	PF02401	LytB protein
IPI	NP_001325698.1	PF00293	NUDIX domain
FPS	DQ059550	PF00348	Polyprenyl synthetase
bAS[b]	PG00849	PF00432	Prenyltransferase and squalene oxidase repeat
		PF13243	Prenyltransferase-like
OAS	PG19915	PF00067	Cytochrome P450
DDS[b]	GU183405	PF13243	Prenyltransferase-like
		PF00432	Prenyltransferase and squalene oxidase repeat
PPDS	JX569336.1	PF00067	Cytochrome P450
PPTS	PG26599	PF00067	Cytochrome P450
LAS[b]	PG22917	PF00432	Prenyltransferase and squalene oxidase repeat
CAS		No Pfam	PANTHER:PTHR11764
			Gene3D:G3DSA:1.50.10.20
			SUPERFAMILY:SSF48239
SS	AB115496	PF00494	Squalene/phytoene synthase
GGPS	KM486564	PF00348	Polyprenyl synthetase
SE[c]	AGK62446.1	PF01266	FAD dependent oxidoreductase
		PF08491	Squalene epoxidase
SE[c]	PG16025	PF13450	NAD(P)-binding Rossmann-like domain
		PF08491	Squalene epoxidase
DDS[b]	PG36431	PF00432	Prenyltransferase and squalene oxidase repeat
DDS[b]	AB122080	PF13243	Prenyltransferase-like
		PF00432	Prenyltransferase and squalene oxidase repeat
UGT	JX018210	PF00201	UDP-glucoronosyl and UDP-glucosyl transferase

[a] Genes with the same domain of “GHMP kinases N terminal domain (PF00288)”, [b] Genes with the same domains of “Prenyltransferase-like (PF13243)” and “Prenyltransferase and squalene oxidase repeat (PF00432)”, [c] SE was annotated based on the presence of “Squalene epoxidase”domain.

Supplementary Table S13. Statistics of triterpenoid biosynthesis genes.

Pathways	Acronym	Pgr	Han	Dca	Pno	Pgi	Cca	Vvi
MVA	AACT	4	4	5	3	5	4	4
	HMGS	1	1	1	2	2	1	2
	HMGR	4	10	3	8	10	2	3
	MVK	3	3	6	5	4	4	4
	PMK	1	5	4	3	4	3	3
	MVD	2	1	1	0	0	1	1
MEP	DXS	4	5	4	3	2	4	5
	DXR	1	3	1	2	1	2	1
	MCT	1	3	2	1	1	1	1
	CMK	5	3	2	2	6	2	1
	MDS	1	1	1	1	2	1	1
	HDS	3	6	2	2	2	2	1
	HDR	1	4	4	3	4	1	1
IPP isomerase	IDI	36	42	32	20	41	25	26
IPP-related downstream	GGPS	31	5	8	6	10	3	3
	FPS	6	12	5	2	7	4	4
	SS	4	6	6	7	13	5	5
	SE	6	6	5	8	12	5	4
OSC group *	bAS	24	11	7	12	10	20	17
	CAS	2	1	4	4	2	1	8
	DDS	5	4	5	3	2	8	0
	LAS	1	1	0	1	3	1	0
Others	CYP450s	362	521	394	231	349	454	322
	UGTs	192	276	216	128	167	265	221

* Pgr: *Platicodon grandiflorus* ; Hna: *Helianthus annuus* L. Dca: *Daucus carota* ; Pno: *Panax notoginsen*

Supplementary Table S14. Expression profile of triterpenoid saponin biosynthesis (TSB)-related genes classified by phylogenetic analysis.

Pathway	Class	Gene ID	FPKM in various tissues								FPKM in methyl jasmonate (MJ) treatment			
			Root	Leaf	Stem	Seed	Pistil	Sepal	Stamen	Petal	(Average) CT 12h	(Average) MJ 12h	(Average) MJ 24h	(Average) MJ 48h
MVA	AACT	PGJG121320	224.16	73.10	129.09	289.14	636.56	590.72	305.30	83.30	159.11	321.58	297.30	229.61
		PGJG136700	62.74	38.07	76.04	37.34	54.15	60.35	70.92	6.87	49.23	39.20	46.12	49.92
		PGJG222140	139.19	183.83	249.18	247.55	310.14	297.43	321.57	175.26	220.33	544.05	320.62	296.89
		PGJG260460	24.05	34.50	51.95	43.03	62.36	80.98	104.08	20.77	34.82	150.91	166.50	162.22
	HMGS	PGJG093370	135.61	50.10	113.08	190.86	340.47	384.33	161.77	317.35	74.18	168.86	168.03	149.34
	HMGR	PGJG045800	10.06	1.30	8.30	7.08	1.32	3.97	6.27	118.37	6.83	4.57	4.23	4.03
		PGJG111050	18.16	13.13	20.51	3.07	248.59	14.68	18.56	1108.92	13.87	27.41	24.69	31.04
		PGJG112050	13.40	1.44	2.48	3.20	30.45	12.93	7.24	202.76	7.56	28.65	19.31	17.88
	MVK	PGJG355140	63.87	12.64	35.78	13.15	108.59	56.93	20.87	125.49	34.50	74.39	81.79	64.53
		PGJG048490	10.73	4.73	47.16	44.86	21.85	26.49	8.40	31.09	24.58	26.50	31.35	30.01
		PGJG167220	51.12	56.45	84.08	41.27	83.18	73.00	8.46	74.01	80.23	90.02	87.70	91.72
	PMK	PGJG089710	17.34	5.62	8.38	6.04	9.30	10.00	3.04	9.15	9.97	12.53	12.38	12.44
	MVD	PGJG160890	45.00	7.42	18.47	25.48	30.34	19.13	39.75	22.70	18.55	39.15	29.92	26.28
		PGJG247740	0.00	0.00	0.00	0.00	0.00	0.00	0.06	0.00	0.00	0.03	0.03	0.00
MEP	DXS	PGJG080430	5.83	0.26	0.27	0.15	0.09	1.15	0.46	0.18	0.26	17.99	39.76	20.20
		PGJG113170	3.29	12.00	18.81	2.24	23.57	9.56	25.04	0.18	34.47	28.72	31.58	47.54
		PGJG317380	21.99	11.61	61.31	7.63	51.16	82.36	109.92	10.50	12.73	33.97	31.65	26.86
		PGJG376280	8.35	5.87	7.70	5.35	6.73	6.60	6.11	0.43	7.92	6.35	6.06	6.83
	DXR	PGJG132480	47.93	150.81	120.59	27.94	105.11	209.69	267.79	6.19	141.64	106.88	101.91	111.17
	MCT	PGJG210470	21.79	57.91	38.76	25.19	37.28	36.45	74.74	11.78	97.34	63.97	62.26	70.55
	CMK	PGJG378770	19.21	28.17	28.12	9.97	34.05	40.77	1.47	51.94	34.62	33.73	26.18	28.62
		PGJG320990	0.00	0.00	0.00	0.00	0.00	0.00	0.00	0.00	0.00	0.00	0.00	0.00
		PGJG278260	7.22	0.57	1.32	1.23	3.54	4.36	3.62	3.17	3.35	6.21	3.12	3.72
		PGJG140850	5.84	1.47	4.20	1.87	2.68	2.38	2.37	1.92	2.51	4.68	4.47	1.57
		PGJG372470	0.00	0.00	0.00	0.00	0.00	0.00	0.00	0.00	0.00	0.00	0.00	0.00
	MDS	PGJG055240	60.67	258.44	189.54	43.42	139.96	119.90	204.60	17.82	343.11	256.79	318.81	328.69
	HDS	PGJG195000	1.41	5.44	4.07	0.42	3.26	2.05	3.51	0.14	3.96	3.46	3.54	4.44
		PGJG195030	1.33	6.23	3.97	0.88	3.03	2.01	3.52	0.06	3.89	2.76	3.06	3.80
		PGJG320710	0.00	0.00	0.00	0.00	0.00	0.00	0.00	0.32	0.00	0.00	0.00	0.00
	HDR	PGJG036730	67.26	667.77	386.99	56.68	308.60	207.41	324.84	10.46	560.46	352.86	395.73	452.97
		PGJG000490	5.83	7.15	6.24	5.84	5.91	6.62	7.20	1.37	153.14	89.53	75.53	79.48
		PGJG025470	0.00	0.00	0.00	0.00	0.00	0.00	0.00	0.00	0.00	0.00	0.00	0.00
		PGJG035260	4.94	1.41	3.75	2.26	2.73	4.50	0.16	3.35	1.79	3.12	1.58	1.89
		PGJG050910	0.00	0.00	0.00	0.00	0.00	0.00	0.00	0.00	0.00	0.00	0.00	0.00
		PGJG090910	0.00	0.00	0.00	0.00	0.00	0.00	0.00	0.00	0.00	0.00	0.00	0.00
		PGJG115150	0.00	0.00	0.00	0.00	0.61	0.00	2.42	0.92	0.00	0.00	0.00	0.00
		PGJG129700	0.00	0.00	0.00	0.00	0.00	0.00	0.00	0.00	0.00	0.00	0.00	0.00
		PGJG130870	10.30	12.74	14.87	5.73	10.57	14.29	1.06	7.59	10.33	9.00	6.30	7.79
		PGJG167180	21.18	81.29	75.72	27.96	87.87	132.85	22.28	66.07	86.81	104.16	119.86	99.12
		PGJG173620	0.00	0.00	0.00	0.00	0.00	0.00	0.00	0.00	0.00	0.00	0.00	0.00
		PGJG186720	0.00	0.00	0.00	0.00	0.00	0.00	0.00	0.00	0.00	0.00	0.00	0.00
		PGJG186750	0.00	0.00	0.00	0.00	0.00	0.00	0.00	0.00	0.00	0.00	0.00	0.00
		PGJG192510	0.00	0.00	1.59	0.00	0.00	0.00	0.00	0.00	0.00	0.00	0.31	0.72
		PGJG276020	0.29	0.00	0.00	0.95	0.00	0.00	1.55	0.00	0.41	0.32	0.94	0.37
		PGJG290940	0.00	0.00	0.00	0.00	0.00	0.00	0.00	0.00	0.00	0.00	0.00	0.00

IPP-related downstream	GGPS	PGJG302550	0.00	0.00	0.00	0.00	0.00	0.00	0.00	0.00	0.13	0.00	0.00	0.00
		PGJG304840	0.88	0.00	1.64	0.20	10.22	5.78	6.42	3.17	0.45	4.41	4.32	4.04
		PGJG304990	0.00	0.00	0.00	0.00	0.00	0.00	0.00	0.00	0.00	0.00	0.00	0.00
		PGJG305170	8.10	0.09	0.62	26.42	1.81	0.15	0.00	0.09	1.07	1.99	1.47	1.41
		PGJG305190	0.00	0.00	0.00	0.00	0.00	0.00	0.00	0.00	0.00	0.00	0.00	0.00
		PGJG326010	0.00	0.00	0.00	0.00	0.00	0.00	0.00	0.00	0.00	0.00	0.00	0.00
		PGJG326950	0.00	0.00	0.00	0.00	0.00	0.00	0.00	0.00	0.00	0.00	0.00	0.00
		PGJG326960	0.00	0.00	0.00	0.00	0.00	0.00	0.00	0.00	0.00	0.00	0.00	0.00
		PGJG339290	41.13	68.62	48.99	23.32	83.36	217.39	5.81	38.62	47.66	423.26	451.74	218.45
		PGJG358780	0.00	0.00	0.00	0.00	0.00	0.00	0.00	0.00	0.00	0.00	0.00	0.00
		PGJG358790	0.00	0.00	0.00	0.00	0.00	0.00	0.00	0.00	0.00	0.00	0.00	0.00
		PGJG371430	0.00	0.00	0.00	0.00	0.00	0.00	0.00	0.00	0.00	0.00	0.00	0.00
		PGJG384970	25.41	23.60	25.91	23.96	26.70	41.52	8.85	16.67	23.67	21.92	18.02	16.23
		PGJG395840	0.00	0.00	0.00	0.00	0.00	0.00	0.00	0.00	0.00	0.00	0.00	0.00
		PGJG396680	0.00	0.00	0.00	0.00	0.00	0.00	0.00	0.00	0.00	0.00	0.00	0.00
		PGJG402880	0.00	0.00	0.00	0.00	0.00	0.00	0.00	0.00	0.00	0.00	0.00	0.00
	FPS	PGJG358070	66.47	27.12	28.30	25.06	44.83	40.54	38.38	49.60	33.21	39.48	28.03	27.17
		PGJG011570	0.00	0.00	0.00	0.00	0.00	0.00	0.00	0.00	0.00	0.00	0.00	0.00
		PGJG266130	13.29	16.07	15.21	8.69	20.77	18.09	18.42	34.76	18.88	27.98	20.10	19.89
		PGJG024210	3.63	72.25	24.04	1.95	10.51	17.76	0.72	39.50	68.57	49.88	42.24	55.22
		PGJG193730	0.24	0.26	0.00	0.13	0.12	0.14	0.00	0.12	0.14	0.33	0.20	0.32
		PGJG304980	6.22	9.03	5.13	2.69	3.20	5.91	0.07	4.61	6.06	4.80	3.93	4.77
	SS	PGJG018830	0.52	0.00	0.00	0.09	0.00	0.00	0.00	0.00	0.02	0.08	0.02	0.02
		PGJG076620	4.12	1.86	3.21	2.11	2.63	3.35	3.99	1.32	2.59	3.20	2.39	1.87
		PGJG164800	77.88	83.58	86.87	82.72	109.36	85.16	94.39	252.97	55.38	98.05	150.71	197.10
		PGJG394240	22.39	224.09	141.91	4.24	5.56	86.65	276.63	0.67	244.54	144.58	185.00	242.38
	SE	PGJG006900	86.82	0.65	1.97	6.59	24.59	40.26	10.53	357.01	8.89	5.79	3.02	2.71
		PGJG040060	52.88	44.51	18.48	97.00	61.96	106.09	41.68	1870.31	78.68	35.88	27.86	24.83
		PGJG238500	45.65	47.82	72.42	43.42	636.67	48.52	33.51	36.08	81.45	81.06	67.98	115.68
		PGJG322340	0.40	1.13	0.46	1.29	1.11	0.78	1.34	0.10	2.45	2.42	1.91	1.96
		PGJG322360	0.15	0.00	0.00	0.00	0.11	0.04	0.04	0.00	0.04	0.02	0.08	0.08
		PGJG379250	0.00	0.00	0.00	0.00	0.09	0.00	0.00	0.00	0.10	0.00	0.10	0.05
OSC group *	bAS	PGJG010030	1.11	1.84	1.48	50.83	1.10	1.23	0.39	1.61	1.68	1.11	0.96	1.49
		PGJG010040	1.00	1.18	0.88	88.78	0.72	0.51	0.75	1.06	2.30	0.95	0.50	1.06
		PGJG011230	38.92	22.33	34.61	24.08	48.77	58.92	2.80	31.95	27.67	25.40	24.31	25.63
		PGJG046170	84.86	40.27	61.11	71.56	78.25	84.88	5.90	50.11	32.04	30.14	27.35	28.41
		PGJG074440	0.02	0.35	1.22	0.00	0.10	0.24	0.00	0.06	0.01	0.01	0.02	0.00
		PGJG099090	45.72	20.99	38.87	22.01	40.63	53.32	2.70	28.45	29.54	29.96	27.64	29.74
		PGJG099910	0.12	0.00	0.14	0.26	0.00	0.00	0.00	0.12	0.21	0.34	0.13	0.65
		PGJG103300	0.05	65.62	52.13	9.04	9.25	9.95	3.30	39.42	33.58	27.74	41.87	65.31
		PGJG137900	0.04	0.31	6.05	0.00	0.07	0.57	0.00	0.06	0.02	0.02	0.07	0.04
		PGJG137910	0.05	0.22	0.22	1.23	2.99	7.27	0.76	51.85	1.74	0.99	1.20	1.22
		PGJG137980	12.05	3.90	5.38	12.94	13.26	8.36	3.97	6.88	4.47	5.39	7.52	5.08
		PGJG149360	0.06	0.00	0.00	0.44	0.00	0.00	0.00	0.02	0.00	0.00	0.01	0.00
		PGJG149370	0.00	0.00	0.00	0.02	0.00	0.00	0.00	0.00	0.00	0.00	0.00	0.01
		PGJG197040	0.17	0.00	0.00	0.35	0.00	0.00	0.00	0.00	0.00	0.00	0.00	0.00
		PGJG204380	58.93	0.00	0.10	0.18	0.00	0.00	0.02	0.00	0.41	0.33	0.35	0.45
		PGJG251010	21.32	7.96	15.39	11.44	23.77	18.37	8.76	16.21	11.30	19.47	18.18	18.53
		PGJG255920	0.00	6.86	7.73	0.00	1.55	9.74	0.38	48.56	5.35	3.95	8.78	12.92
		PGJG274860	22.78	8.34	8.72	13.14	7.81	5.73	1.57	3.61	0.00	1.97	5.72	0.68
		PGJG290560	2.13	1.54	1.23	0.21	0.43	0.45	2.37	0.19	2.39	1.85	1.56	3.86
		PGJG297250	0.00	0.00	0.00	0.00	0.00	0.00	0.00	0.00	0.00	0.00	0.00	0.00
		PGJG325090	0.71	0.00	0.00	0.00	0.00	0.00	0.00	0.00	0.03	0.01	0.00	0.01
		PGJG364670	24.65	6.89	33.21	14.21	209.00	54.34	9.02	82.97	12.33	15.23	14.14	36.92

		PGJG380380	0.00	0.00	0.00	0.00	0.00	0.00	0.00	0.00	0.00	0.00	0.00	0.00
		PGJG395670	0.00	7.70	8.54	0.00	1.14	3.33	0.07	76.91	9.14	12.36	11.48	19.10
	CAS	PGJG178930	0.09	0.03	0.11	0.00	0.19	0.00	0.24	0.03	0.06	0.12	0.00	0.10
		PGJG197020	41.25	13.06	27.76	29.08	39.60	30.52	12.77	27.29	21.70	32.06	34.94	29.68
	DDS	PGJG207790	0.00	0.49	0.00	0.05	120.25	32.66	1.87	333.69	21.63	21.08	26.26	36.22
		PGJG053400	0.00	0.02	0.00	0.00	1.43	0.13	0.00	0.43	5.98	7.84	5.03	6.31
		PGJG080030	0.02	0.00	0.00	0.00	0.00	0.00	0.00	0.04	0.00	0.07	0.01	0.00
		PGJG103290	0.13	117.47	78.45	18.68	15.94	16.47	5.85	59.39	76.74	40.11	76.57	104.28
		PGJG395650	0.00	16.75	17.91	0.00	1.46	6.91	0.00	160.97	22.29	28.97	26.66	44.59
	LAS	PGJG197050	6.61	3.94	6.40	1.22	1.98	3.81	0.06	1.16	3.04	1.51	1.46	2.00

Supplementary Table S15. qRT-PCR validation for *GGPS* pralogs in *P. grandiflorus* .

Class	Gene ID	qRT-PCR validation					
		Log_2(Ct value)				Primer	
		Root	Leaf	Stem	Flower	Forward	Reverse
GGPS	PGJG000490	3.11	7.68	2.34	4.47	AATCGGGGAATTAGCCAAA	AGTATCGCTCCCAACACCAC
	PGJG025470	0.274131675	3.178130368	0.553015987	0.285281729	GACAACGACGACCTCCGT	CCGATGTCCCTTTAGTTTCCA
	PGJG035260	6.864088334	8.629039649	4.166199942	4.983853985	TCTACACATCCCGACATCCA	ACCAGTGGTTACCGAGTGC
	PGJG050910	0.222765949	1.763468181	0.343999242	0.083470054	TTCGAGGAAGACATCGTCG	AGAACATATATCAACCACTTGACCTAC
	PGJG090910	0	0	0	0	CTTGCACCGTCGAGATGATC	AGCGCGTCTCTTACCAAAAC
	PGJG115150	0.800560153	3.824244923	1.048479654	0.214186892	GGTGGTCACCAATCCACTGT	CGACGTCCTCGTCGTAGACT
	PGJG129700	0.668381206	4.201769426	1.022402032	0.148053442	ATACCCAAACCCAGATCATCTTCT	GTGCCGCTGACAAGTTTACA
	PGJG130870	3.197521002	4.897573829	5.102491061	3.536048603	TGAAAGACAGCACGACCTTG	TAGCTCCATGTGGTGTTCCA
	PGJG167180	1.891959514	7.147485287	1.522849761	3.337410098	CGTTGGGGTTTTGTATCAGG	TTTCTTCGCCTTGGCTCTTA
	PGJG173620	4.504011431	20.2304041	3.508379444	8.362355126	TCGTGGATGACATTCTGGAC	TGCTCCTTAGCCTCTCGATT
	PGJG186720	0	0	0	0	GCAAACCCACAAACCACAAG	GTTGCGTTCAGTCGGTCTTT
	PGJG186750	0	0	0	0	TATTTGGACAACGACGACCA	GCTGCCAAGCAAGATGAACT
	PGJG192510	25.04	8.25	33.17	22.61	ATTCTCTACATCGCCGCCT	CGTCGTTGTCCATACACAGG
	PGJG276020	0.00	0.00	0.00	0.00	GCAATCTTGCTTGCATCACT	ATCCATAACCAAATCCTTCCCTA
	PGJG290940	0	0	0	0	AGCAAACCCACAAACCACAA	TTCCCCAATGACCCTCACAA
	PGJG302550	0	0	0	0	GTTCAACGTGATCGATTCCA	TCACCATAGCACTGGCAAAG
	PGJG304840	8.917688465	3.595430117	0.951934469	3.571949353	TGGCTTTGAAAGTGCTTCTG	TCATCCTCCCATCCATTTGT
	PGJG304990	0.438198128	5.200692962	1.303236891	0.14030164	CTAGCCGCTTGCGTCGTT	TGGCAACGTCTTCCCTAAAC
	PGJG305170	2.964926433	7.330485753	2.541170282	0.733889398	GACTGCAGGAAAGGACTTGG	TGCTAAATGATACAATGGTGCTG
	PGJG305190	0.611009955	5.945767367	1.462284151	0.139063709	GTGAGGATGTTGCCGCAC	TCGAGGGCTGAAAGAGAAAA
	PGJG326010	0.725951075	2.507154126	1.252570892	0.203199862	GTTGGCAGTCACCAATCCAC	GAGTCACCGGCGAGTACG
	PGJG326950	0	0	0	0	CAGCAACTAAAGGGACGTCG	GCTCAACGGCAACATCAGTA
	PGJG326960	0	0	0	0	ACTGATGTTGCCGTTAAGCA	ATATGGGTTCTGCCGATGAG
	PGJG339290	15.64343582	68.47726012	49.24192474	9.431548722	GCTAGGTGCATTGGGTTGTT	TTCAGCAAACTCCCTCGACT
	PGJG358780	1.769110938	5.990503645	1.575553642	1.010971403	ACCATCGCCATGTCAGCTA	GAACATTGTACGAAAAATTGTTGG
	PGJG358790	0	0	0	0	GCACCGTCGAGATGATTCAC	AAAATTGTTGGTCGCGACGT
	PGJG371430	0.657252424	4.449353182	1.056947942	0.149324098	GTGAAATGCGTGGCGAGAT	ACAAGTTTTCCAAGCCGTTG
	PGJG384970	12.91123061	14.19551857	23.30619933	8.214636815	AAGAAGGGGAGCGACTCCTA	GGTGCCCTTGGATTTGTAGA
	PGJG395840	0.063457188	1.932397885	0.197113477	0.073891647	ATCACCATGCCAGCCAAT	GCGACATCTTCCTCGAACA
	PGJG396680	0	0	0	0	CCCAGACCATCGTCATGCTA	GGTTGAAGGTGGGAAGATGT
	PGJG402880	1.535090018	4.882615371	0.649240732	0.283872242	GGTCCGAGATGGATCGTTT	CAGGATAGAGAATATCGCGGTAAA

Supplementary Table S16. Statistics of whole-genome bisulfite sequencing (WGBS) data after quality control.

Sample	Replicate	No. of total reads	No. of aligned reads	No. of aligned reads after deduplication	Mapping rate (%)	Total Cs	Fold coverage	%Methylation (CG)	%Methylation (CHG)	%Methylation (CHH)	Methylated CGs	Unmethylated CGs	Methylated CHGs	Unmethylated CHGs	Methylated CHHs	Unmethylated CHHs
Control 12h-1	Triplicates	80,798,817	33,085,005	26,250,767	32.49	830,876,711	7.49	89.6	73.5	19.8	111,642,242	12,970,631	101,758,421	36,652,527	112,693,153	455,159,737
Control 12h-2		92,769,277	36,949,928	28,845,803	31.09	906,699,389	8.22	88.4	70.5	16.4	119,187,434	15,696,855	106,328,488	44,481,505	101,841,843	519,163,264
Control 12h-3		76,442,644	30,712,080	24,906,248	32.58	783,666,313	7.08	89.0	72.5	17.5	103,626,986	12,810,506	94,588,104	35,809,394	93,874,115	442,957,208
MJ 12h-1	Triplicates	77,365,339	31,451,390	25,619,279	33.11	806,089,983	7.35	89.7	74.0	21.5	107,645,297	12,315,417	98,870,774	34,657,541	118,852,753	433,748,201
MJ 12h-2		83,922,727	32,716,871	25,861,281	30.82	805,503,652	7.36	88.5	71.1	16.6	106,315,334	13,827,477	95,750,185	38,935,318	91,620,855	459,054,483
MJ 12h-3		76,923,540	30,715,433	24,745,017	32.17	772,032,379	7.02	88.9	71.8	16.6	103,288,932	12,861,298	92,965,529	36,546,848	87,519,979	438,849,793
MJ 24h-1	Triplicates	74,826,924	30,305,518	24,599,813	32.88	774,123,984	7.04	89.7	73.1	23.5	103,190,282	11,824,872	93,433,327	34,315,677	124,679,843	406,679,983
MJ 24h-2		107,251,672	42,439,173	32,187,919	30.01	1,007,526,434	9.13	88.5	71.2	16.3	133,068,881	17,304,557	119,836,794	48,527,714	112,556,054	576,232,434
MJ 24h-3		76,059,456	30,575,571	24,930,591	32.78	781,313,630	7.08	88.9	72.5	17.8	103,574,211	12,927,932	94,468,677	35,860,881	95,096,469	439,385,460
MJ 48h-1	Triplicates	76,769,577	31,020,298	24,766,348	32.26	786,353,955	7.10	89.7	75.0	24.9	104,959,285	12,038,230	97,074,379	32,357,598	134,617,855	405,306,608
MJ 48h-2		105,358,948	42,274,724	33,257,250	31.57	1,046,782,687	9.44	88.6	70.7	17.5	136,843,525	17,566,007	122,468,272	50,833,695	125,608,145	593,463,043
MJ 48h-3		78,770,854	31,054,511	25,112,294	31.88	777,626,562	7.13	89.1	72.3	17.8	103,832,772	12,706,872	94,103,017	36,088,879	94,439,078	436,455,944

[1] Mapping rate (%) = No. of aligned reads after deduplication x 100 / No. of total reads

[2] The methylation level was calculated as mC/(mC + umC) where mC is the number of methylated reads and umC is the number of unmethylated reads, was calculated for three contexts CG, CHG and CHH, respectively.

('H' represents non-G bases according to the sequence context.)

-

Supplementary Table S17. The information of BUSCOs analyzed from the draft genome assembly of *P. grandiflorus* .

BUSCO ID	Status	Sequence	BUSCO Gene Start	BUSCO Gene End	BUSCO Score	Length (bp)	Description
2at33090	Complete single-copy	Scaffold3301	228255	257829	2720.1	2551	Midasin
5at33090	Complete single-copy	Scaffold1387	372710	396849	3585.5	2426	sacsin
24at33090	Complete single-copy	Scaffold1321	431829	455218	5623	3589	auxin transport protein BIG
255at33090	Complete single-copy	Scaffold2524	216698	272818	3421.7	2159	protein ILITYHIA
383at33090	Fragmented	Scaffold728	64596	81184	970.3	825	WD40-repeat-containing domain
1363at33090	Complete single-copy	Scaffold72	42511	67716	1581.3	1138	UDP-glucose
1419at33090	Complete single-copy	Scaffold1731	107055	123166	1076.2	875	E3 ubiquitin-protein ligase listerin
1557at33090	Complete single-copy	Scaffold4550	78833	124831	1386.5	1019	2-succinyl-5-enolpyruvyl-6-hydroxy-3-cyclohexene-1-carboxylic-acid synthase
1855at33090	Complete single-copy	Scaffold1007	364110	378292	2177.6	1687	thyroid adenoma-associated protein homolog
4004at33090	Complete single-copy	Scaffold4610	13596	59949	1592.5	1111	splicing factor 3B subunit 3
4034at33090	Missing						
4115at33090	Complete single-copy	Scaffold4706	7010	60901	1435.7	911	Glycoside hydrolase, family 2
5305at33090	Complete single-copy	Scaffold4392	212031	244747	1005.1	796	F-box protein
5927at33090	Complete single-copy	Scaffold4201	579020	602789	1286.1	975	Armadillo-type fold
6512at33090	Complete single-copy	Scaffold814	103774	143236	839.6	729	protein timeless homolog isoform X1
6843at33090	Complete single-copy	Scaffold2512	173260	202821	879.5	600	Thioredoxin-like fold
7366at33090	Complete single-copy	Scaffold3417	55972	74292	1372.2	832	Zinc finger, CCHC-type
8072at33090	Complete single-copy	Scaffold2241	137954	167333	1266.1	814	transportin MOS14
9044at33090	Complete single-copy	Scaffold694	41433	52558	1403.3	869	GHMP kinase, C-terminal domain
9058at33090	Complete single-copy	Scaffold4385	247080	261703	1076.3	992	XPG/Rad2 endonuclease
9849at33090	Complete single-copy	Scaffold4623	145383	174844	863.4	806	MMS19 nucleotide excision repair protein homolog isoform X1
10924at33090	Complete single-copy	Scaffold2879	125287	153771	1151.7	728	Alpha-1,6-glucosidases, pullulanase-type
11664at33090	Complete single-copy	Scaffold133	68627	98850	1084.9	701	DNA topoisomerase, type IIA, subunit A/C-terminal
11800at33090	Complete single-copy	Scaffold1815	59034	64012	1975.7	1062	5-oxoprolinase
12439at33090	Complete single-copy	Scaffold4870	69761	88320	1261.1	773	WD40-repeat-containing domain
13039at33090	Complete single-copy	Scaffold480	97968	122998	1283.5	703	Aminoacyl-tRNA synthetase, class Ia
13643at33090	Complete single-copy	Scaffold969	107572	116544	1099.1	710	conserved oligomeric Golgi complex subunit 7
15682at33090	Complete single-copy	Scaffold2766	10474	37176	1070	691	nuclear cap-binding protein subunit 1
16726at33090	Complete duplicated	Scaffold1929	1	2164	530.2	478	ATPase, AAA-type, conserved site
16726at33090	Complete duplicated	Scaffold34	11018	13681	531.5	564	ATPase, AAA-type, conserved site
16726at33090	Complete duplicated	Scaffold4773	139264	142155	542.4	518	ATPase, AAA-type, conserved site
18455at33090	Complete single-copy	Scaffold659	26406	53098	807.3	519	WD40-repeat-containing domain
18801at33090	Complete single-copy	Scaffold4466	231006	254437	967	684	condensin complex subunit 3
21163at33090	Complete single-copy	Scaffold2321	69968	90952	924.4	645	ribonuclease E/G-like protein, chloroplastic isoform X1
21751at33090	Complete single-copy	Scaffold608	498961	537781	1399.8	819	glycine--tRNA ligase, chloroplastic/mitochondrial 2 isoform X1
21857at33090	Complete single-copy	Scaffold4326	247584	266234	722.7	536	anaphase-promoting complex subunit 4
22109at33090	Complete single-copy	Scaffold4485	190927	206680	712.9	431	quinolinate synthase, chloroplastic

22618at33090	Complete single-copy	Scaffold3153	1	7071	909.7	646	BTB/POZ domain-containing protein At2g30600
22958at33090	Complete single-copy	Scaffold1436	59018	96425	1059	695	SAP domain
23444at33090	Complete single-copy	Scaffold4782	88259	97083	577	456	pumilio homolog 23
23853at33090	Complete single-copy	Scaffold287	70084	88471	861	627	probable transmembrane GTPase FZO-like, chloroplastic
24838at33090	Complete single-copy	Scaffold4744	376072	389138	603.5	646	G patch domain-containing protein, N-terminal
25435at33090	Complete single-copy	Scaffold792	70720	106771	1038	715	chromosome transmission fidelity protein 18 homolog
26868at33090	Complete single-copy	Scaffold594	84740	91264	792	507	transcription factor bHLH140
27237at33090	Complete single-copy	Scaffold644	4346	16419	917	626	ribonuclease II, chloroplastic/mitochondrial
27607at33090	Complete single-copy	Scaffold1498	74692	87426	808.7	540	FG-GAP repeat-containing protein
28920at33090	Complete single-copy	Scaffold4049	108249	128921	883.4	546	probable DNA helicase MCM8
31155at33090	Complete single-copy	Scaffold4665	453640	467318	1052.6	749	NatC N(Alpha)-terminal acetyltransferase, Mak10 subunit
31855at33090	Complete single-copy	Scaffold4375	314589	325453	958.7	526	ABC transporter G family member 7
32730at33090	Complete duplicated	Scaffold1473	58293	72971	705.7	434	THO complex subunit 1
32730at33090	Complete duplicated	Scaffold1823	44773	59293	705.7	434	THO complex subunit 1
32923at33090	Complete duplicated	Scaffold1262	79246	90627	1284	752	ubiquitin-like modifier-activating enzyme atg7
32923at33090	Complete duplicated	Scaffold2430	373342	383160	1208.5	699	ubiquitin-like modifier-activating enzyme atg7
33264at33090	Complete single-copy	Scaffold312	131492	139006	721.3	462	Tyrosyl-DNA phosphodiesterase 1
34517at33090	Complete single-copy	Scaffold1309	68285	83927	1015.8	663	conserved oligomeric Golgi complex subunit 5
35499at33090	Complete single-copy	Scaffold4719	40078	48745	904.5	559	FAD/NAD(P)-binding domain
35887at33090	Complete single-copy	Scaffold2227	25199	42153	744.9	539	Rad4 beta-hairpin domain 1
36284at33090	Complete duplicated	Scaffold4577	70711	83047	1099.7	627	dynamin-like protein ARC5
36284at33090	Complete duplicated	Scaffold70	70710	83301	1103.9	629	dynamin-like protein ARC5
37663at33090	Complete single-copy	Scaffold4055	109731	154483	839.4	532	MoeA, C-terminal, domain IV
37699at33090	Complete single-copy	Scaffold298	154988	173290	935.3	1184	DEAD-box ATP-dependent RNA helicase 13
39913at33090	Complete duplicated	Scaffold1963	37078	45199	1060	544	4-hydroxy-3-methylbut-2-en-1-yl diphosphate synthase
39913at33090	Complete duplicated	Scaffold1964	6256	14493	1060	544	4-hydroxy-3-methylbut-2-en-1-yl diphosphate synthase
40630at33090	Missing						
41146at33090	Complete single-copy	Scaffold4879	906	52940	778.1	434	Electron transfer flavoprotein-ubiquinone oxidoreductase, mitochondrial
41244at33090	Complete single-copy	Scaffold519	320762	328993	744.2	437	Imidazole glycerol phosphate synthase hisHF
42407at33090	Complete single-copy	Scaffold4879	92450	111124	926.7	640	pre-mRNA-processing protein 40C
43683at33090	Complete single-copy	Scaffold2310	723779	746945	805.9	505	PPM-type phosphatase domain
43957at33090	Complete single-copy	Scaffold4676	261095	269330	1117.5	694	WD40 repeat
44063at33090	Complete single-copy	Scaffold4235	243642	263197	740.1	429	4-alpha-glucanotransferase
44526at33090	Fragmented	Scaffold2442	358034	362281	483.2	358	GPI transamidase component PIG-T
46307at33090	Complete single-copy	Scaffold4122	52890	63356	841.9	442	methylcrotonoyl-CoA carboxylase beta chain, mitochondrial
48027at33090	Complete single-copy	Scaffold1016	29430	45844	804.8	427	Aminoacyl-tRNA synthetase, class I, anticodon-binding
48290at33090	Complete single-copy	Scaffold4664	176281	184995	635.1	417	probable Ufm1-specific protease
48645at33090	Complete single-copy	Scaffold710	458096	483628	626	449	Cwf19-like, C-terminal domain-1
49092at33090	Complete duplicated	Scaffold2284	377751	389491	844.2	452	Aldehyde dehydrogenase

49092at33090	Complete duplicated	Scaffold2201	1	15181	960.2	509	Aldehyde dehydrogenase
50512at33090	Complete single-copy	Scaffold1365	306261	314609	392.9	351	Predicted protein
50528at33090	Fragmented	Scaffold2774	224488	238380	317.9	253	Light-mediated development protein DET1
51214at33090	Complete single-copy	Scaffold768	96732	104348	645.4	421	RNA helicase
51262at33090	Complete single-copy	Scaffold178	114137	121571	472.8	406	glyoxysomal processing protease, glyoxysomal
52348at33090	Complete duplicated	Scaffold2813	10131	29601	600.1	392	JmjC domain
52348at33090	Complete duplicated	Scaffold1097	36883	58075	600.1	392	JmjC domain
52435at33090	Complete single-copy	Scaffold1400	34330	40154	918.5	455	Phytoene desaturase
52795at33090	Complete duplicated	Scaffold4351	36737	43886	337.8	345	Lysine--tRNA ligase
52795at33090	Complete duplicated	Scaffold970	33204	41287	337.8	345	Lysine--tRNA ligase
52795at33090	Complete duplicated	Scaffold1781	63512	71323	341.7	343	Lysine--tRNA ligase
53416at33090	Complete single-copy	Scaffold4605	29185	36711	525.9	359	Alpha-1,3-glucosyltransferase
53527at33090	Complete single-copy	Scaffold4512	105250	134042	1224.7	716	4-alpha-glucanotransferase
55448at33090	Complete single-copy	Scaffold1284	351870	363820	812.2	420	Prolyl-tRNA synthetase
57004at33090	Complete single-copy	Scaffold722	69088	74167	726.9	444	FAD/NAD(P)-binding domain superfamily
58672at33090	Fragmented	Scaffold4465	632023	641054	302.5	169	Violaxanthin de-epoxidase
60262at33090	Complete single-copy	Scaffold1191	373919	380536	616.7	375	WD40/YVTN repeat-like-containing domain superfamily
62262at33090	Complete single-copy	Scaffold933	44236	56432	429.7	337	putative tRNA pseudouridine synthase Pus10
63411at33090	Complete single-copy	Scaffold2700	313717	322062	533.3	337	fe-S cluster assembly factor HCF101, chloroplastic
63853at33090	Complete single-copy	Scaffold4354	60237	63297	647.7	345	UDP-sulfoquinovose synthase, chloroplastic
64272at33090	Complete single-copy	Scaffold2138	3620	16997	789.3	678	nucleolar protein 14
65263at33090	Complete single-copy	Scaffold1640	45706	58105	474.3	344	cell cycle checkpoint protein RAD17 isoform X1
67132at33090	Complete single-copy	Scaffold503	142664	152539	836.5	496	DNA topoisomerase 6 subunit B
67389at33090	Complete duplicated	Scaffold1092	101509	114053	523.3	407	methyltransferase-like protein 17, mitochondrial
67389at33090	Complete duplicated	Scaffold1766	286716	297101	524.9	403	methyltransferase-like protein 17, mitochondrial
68169at33090	Complete single-copy	Scaffold4420	359692	373912	813.1	635	nuclear pore complex protein NUP88
68537at33090	Complete single-copy	Scaffold1567	346649	355507	528.5	332	Chitobiosyldiphosphodolichol beta-mannosyltransferase
68957at33090	Complete single-copy	Scaffold593	93558	101290	671.8	443	Mannosyltransferase
69073at33090	Complete single-copy	Scaffold3725	156402	167424	464.6	294	Protein arginine methyltransferase NDUFAF7
69137at33090	Complete single-copy	Scaffold4520	502482	514566	641.9	511	CDK5RAP3-like protein
69505at33090	Complete single-copy	Scaffold2497	24754	32721	546.4	389	tRNA A64-2'-O-ribosylphosphate transferase
69614at33090	Complete duplicated	Scaffold1882	75254	82303	561.4	352	WD40-repeat-containing domain
69614at33090	Complete duplicated	Scaffold2771	17367	24511	561.4	352	WD40-repeat-containing domain
70613at33090	Complete single-copy	Scaffold4640	124853	129485	714.9	374	homogentisate 1,2-dioxygenase
71620at33090	Complete single-copy	Scaffold4811	77896	89125	510.3	328	DNA primase large subunit
72992at33090	Complete duplicated	Scaffold4473	134699	155322	627	433	RED-like, N-terminal
72992at33090	Complete duplicated	Scaffold4537	81095	108268	627	433	RED-like, N-terminal
74230at33090	Complete single-copy	Scaffold336	8186	24235	692.9	366	Acyl-CoA N-acyltransferase
74580at33090	Complete single-copy	Scaffold4602	172314	174838	591.2	400	protein DGCR14

74742at33090	Complete duplicated	Scaffold1699	37561	42840	350.7	299	histone acetyltransferase type B catalytic subunit
74742at33090	Complete duplicated	Scaffold821	89479	94758	350.7	299	histone acetyltransferase type B catalytic subunit
75785at33090	Complete single-copy	Scaffold2284	342772	360807	412.8	310	P-loop containing nucleoside triphosphate hydrolase
76026at33090	Complete single-copy	Scaffold2217	192631	210808	485.4	332	histone-lysine N-methyltransferase ATXR2
76849at33090	Complete duplicated	Scaffold4566	228368	234863	723.3	457	mini-chromosome maintenance complex-binding protein
76849at33090	Complete duplicated	Scaffold942	108745	115240	723.3	457	mini-chromosome maintenance complex-binding protein
79950at33090	Complete single-copy	Scaffold891	111065	131549	393.8	271	Rhodanese-like domain
83749at33090	Complete single-copy	Scaffold3911	250899	259628	603.2	342	3-oxoacyl-
84114at33090	Complete single-copy	Scaffold4764	76732	93523	511.5	336	Lysine-tRNA ligase
84264at33090	Complete single-copy	Scaffold4632	167422	183211	509.3	604	WW domain
87347at33090	Complete single-copy	Scaffold36	34958	45884	1212.3	744	WD40 repeat
87734at33090	Complete single-copy	Scaffold3018	7246	14895	284.4	232	DEAD-box ATP-dependent RNA helicase 58, chloroplastic isoform X1
88295at33090	Complete single-copy	Scaffold4214	302972	308096	470.1	329	Rab3-GAP regulatory subunit
88687at33090	Complete duplicated	Scaffold1772	11406	22530	477.8	407	cell cycle checkpoint control protein RAD9A
88687at33090	Complete duplicated	Scaffold957	65058	72097	493.2	389	cell cycle checkpoint control protein RAD9A
88771at33090	Complete single-copy	Scaffold2065	48810	58278	536	390	Tetratricopeptide-like helical domain superfamily
89796at33090	Fragmented	Scaffold4714	57538	61690	579.5	405	conserved oligomeric Golgi complex subunit 6
90240at33090	Complete duplicated	Scaffold3713	199975	208806	515.4	331	PUA domain
90240at33090	Complete duplicated	Scaffold4897	4879	13701	515.6	331	PUA domain
90301at33090	Complete single-copy	Scaffold4541	230637	242801	683.6	371	Pyridoxal phosphate-dependent transferase
90872at33090	Complete single-copy	Scaffold438	78690	89989	632.2	338	Cation efflux protein
92418at33090	Complete single-copy	Scaffold4259	22810	27472	578.8	292	uncharacterized protein ycf39
92579at33090	Complete duplicated	Scaffold1374	12556	30023	494.3	351	rhomboid-like protein 15
92579at33090	Complete duplicated	Scaffold414	190963	208394	488.9	356	rhomboid-like protein 15
95807at33090	Fragmented	Scaffold4642	104239	121374	318.1	265	ApaG domain
96953at33090	Complete single-copy	Scaffold2257	25190	31901	326.3	284	WD40-repeat-containing domain
97593at33090	Complete duplicated	Scaffold4363	156082	161678	441	299	Origin of replication complex subunit 4
97593at33090	Complete duplicated	Scaffold1674	27382	33072	441	299	Origin of replication complex subunit 4
101241at33090	Complete single-copy	Scaffold464	84136	94070	565.4	364	actin-related protein 6
101701at33090	Complete single-copy	Scaffold226	280809	304944	783.2	501	UTP--glucose-1-phosphate uridylyltransferase 3, chloroplastic
102307at33090	Complete single-copy	Scaffold4634	172126	176885	591.3	293	WD40-repeat-containing domain
103267at33090	Complete single-copy	Scaffold4349	171223	178563	425	326	recQ-mediated genome instability protein 1
103382at33090	Complete single-copy	Scaffold2115	22616	29057	318.2	227	Prolyl 3-hydroxylase 1
104218at33090	Complete single-copy	Scaffold4372	210665	223997	514.7	298	cysteine synthase 2
104557at33090	Complete single-copy	Scaffold2506	7507	39761	464.2	326	Coenzyme F420 hydrogenase/dehydrogenase beta subunit, C-terminal
104821at33090	Complete single-copy	Scaffold708	106483	109293	493.6	349	RNA 2-O ribose methyltransferase, substrate binding
104867at33090	Complete single-copy	Scaffold4328	275023	290242	940.4	603	aminodeoxychorismate synthase, chloroplastic
105044at33090	Complete single-copy	Scaffold3054	126091	141302	926	589	putative DEAD-box ATP-dependent RNA helicase 29
105782at33090	Complete single-copy	Scaffold3776	70054	75074	433.8	297	Cobalamin (Vitamin B12) biosynthesis CobW-like,C-terminal

105867at33090	Complete single-copy	Scaffold4457	40162	47976	493.2	301	alcohol dehydrogenase
106769at33090	Complete single-copy	Scaffold4743	205639	215381	624.9	325	4-diphosphocytidyl-2-C-methyl-D-erythritol kinase, chloroplastic
106886at33090	Complete duplicated	Scaffold736	89112	91946	346.7	263	protein CDC73 homolog
106886at33090	Complete duplicated	Scaffold4476	89088	91922	346.7	263	protein CDC73 homolog
107107at33090	Complete single-copy	Scaffold3911	184954	193691	477.4	366	Ubiquitin-like domain superfamily
107413at33090	Complete single-copy	Scaffold4530	178762	192216	475.6	356	GPI mannosyltransferase 1
108705at33090	Complete single-copy	Scaffold526	125390	155878	450.2	375	uncharacterized protein At4g17910 isoform X1
109586at33090	Complete single-copy	Scaffold92	154112	166468	773.3	445	Methionyl-tRNA synthetase
109823at33090	Complete single-copy	Scaffold4519	9126	20624	454.1	263	protein LOW PSII ACCUMULATION 3, chloroplastic
110222at33090	Complete single-copy	Scaffold66	16241	28426	495.7	409	DnaJ domain
110225at33090	Complete single-copy	Scaffold2845	37436	49978	805	493	riboflavin biosynthesis protein PYRR, chloroplastic
111210at33090	Complete single-copy	Scaffold494	39844	50087	464.8	271	HAD superfamily
111213at33090	Complete single-copy	Scaffold3330	44741	56490	405.7	244	epimerase family protein SDR39U1 homolog, chloroplastic
111249at33090	Complete duplicated	Scaffold2556	1	11631	380.6	240	predicted protein
111249at33090	Complete duplicated	Scaffold1273	21	20989	380.7	240	predicted protein
111924at33090	Complete single-copy	Scaffold183	30232	40191	433.1	276	protein arginine N-methyltransferase 2
112238at33090	Complete duplicated	Scaffold19	208780	215014	775	532	Origin recognition complex subunit 1
112238at33090	Complete duplicated	Scaffold182	11137	17611	774	532	Origin recognition complex subunit 1
112325at33090	Complete single-copy	Scaffold4842	46781	52893	375.3	295	sister chromatid cohesion protein DCC1
112461at33090	Complete single-copy	Scaffold2626	339505	360479	680.2	388	FAD-binding, type 2
112542at33090	Complete duplicated	Scaffold2954	209763	214363	457.7	246	cytochrome c-type biogenesis ccda-like chloroplastic protein
112542at33090	Complete duplicated	Scaffold1462	49426	56896	457.7	246	cytochrome c-type biogenesis ccda-like chloroplastic protein
113024at33090	Complete single-copy	Scaffold4643	134396	139880	340.6	244	Queuosine salvage protein
114996at33090	Complete single-copy	Scaffold4244	164390	178774	1283.9	938	Elongator complex protein 1
115240at33090	Complete single-copy	Scaffold528	104764	118756	419.5	292	Ubiquitin domain
115992at33090	Complete duplicated	Scaffold227	1	1010	409.1	254	WD40-repeat-containing domain
115992at33090	Complete duplicated	Scaffold1110	431187	432339	409.1	254	WD40-repeat-containing domain
116411at33090	Complete duplicated	Scaffold68	178814	184276	177.6	221	WD40/YVTN repeat-like-containing domain superfamily
116411at33090	Complete duplicated	Scaffold1399	338870	343349	164.1	219	WD40/YVTN repeat-like-containing domain superfamily
116516at33090	Complete single-copy	Scaffold4276	167853	179307	602.2	354	preprotein translocase subunit SCY1, chloroplastic
116585at33090	Fragmented	Scaffold2674	394059	399221	258.2	180	peroxisome biogenesis factor 10
116623at33090	Complete single-copy	Scaffold2786	112161	123122	381.7	279	zinc finger HIT domain-containing protein 2
117191at33090	Complete single-copy	Scaffold1002	28175	49424	416	280	NADH dehydrogenase
117247at33090	Complete single-copy	Scaffold62	45118	77337	474.8	272	Protein phosphatase methylesterase 1
119439at33090	Complete single-copy	Scaffold2043	38138	53178	392.7	298	Arginine biosynthesis bifunctional protein ArgJ, chloroplastic
120031at33090	Complete single-copy	Scaffold2495	5481	8751	371.7	331	FAD dependent oxidoreductase
120338at33090	Complete single-copy	Scaffold3566	1051485	1066481	638.3	368	Methylthiotransferase, N-terminal
120455at33090	Complete single-copy	Scaffold2873	5359	23191	313.5	185	Uracil-DNA glycosylase
120589at33090	Complete duplicated	Scaffold4840	200343	215466	639	423	beta-catenin-like protein 1

120589at33090	Complete duplicated	Scaffold1719	685484	695709	662	429	beta-catenin-like protein 1
121281at33090	Complete single-copy	Scaffold4801	615945	626615	415.3	276	Formyl transferase, C-terminal
121303at33090	Complete single-copy	Scaffold4444	258531	268173	574.4	361	Alpha/Beta hydrolase fold
122367at33090	Complete single-copy	Scaffold4442	190136	191776	490.1	335	Amine oxidase
122422at33090	Complete single-copy	Scaffold541	190497	197348	395.5	246	dehydrogenase/reductase SDR family member 12
122831at33090	Complete single-copy	Scaffold4706	115506	121381	458.7	325	DEAD-box ATP-dependent RNA helicase 22
122958at33090	Complete single-copy	Scaffold1251	67116	96969	471.5	294	Leucine carboxyl methyltransferase 1 homolog
123838at33090	Complete single-copy	Scaffold61	18239	22603	356.7	229	alpha-ketoglutarate-dependent dioxygenase alkB
125550at33090	Complete single-copy	Scaffold4362	88687	93059	170.4	135	Regulatory protein recX
125747at33090	Complete single-copy	Scaffold3275	102993	112297	264.8	205	predicted protein
126138at33090	Complete single-copy	Scaffold4629	98524	111320	626.2	439	DnaJ domain
126427at33090	Complete duplicated	Scaffold1789	65279	68345	401.7	264	Methionine adenosyltransferase 2 subunit beta
126427at33090	Complete duplicated	Scaffold191	380581	383519	401.7	264	Methionine adenosyltransferase 2 subunit beta
126427at33090	Complete duplicated	Scaffold4518	232169	235123	401.7	264	Methionine adenosyltransferase 2 subunit beta
127059at33090	Complete single-copy	Scaffold999	86901	106788	601.8	320	Tryptophan-tRNA ligase
128774at33090	Complete single-copy	Scaffold3389	11246	20117	298.5	222	Proteasome assembly chaperone 2
130268at33090	Complete duplicated	Scaffold4055	180983	208889	407.2	258	putative methyltransferase At1g22800
130268at33090	Complete duplicated	Scaffold3448	4173	5773	407.9	258	putative methyltransferase At1g22800
130279at33090	Complete single-copy	Scaffold1550	34188	54799	383.3	253	Putative plastid-lipid-associated protein 12, chloroplastic
130637at33090	Complete single-copy	Scaffold1411	648785	655071	236.5	246	cyclin-dependent kinase E-1
131556at33090	Complete duplicated	Scaffold754	37842	45494	504.1	303	SUF system FeS cluster assembly, SufBD
131556at33090	Complete duplicated	Scaffold753	43266	50961	504.1	303	SUF system FeS cluster assembly, SufBD
131874at33090	Complete duplicated	Scaffold2722	19136	25901	681	937	ABC transporter B family member 28
131874at33090	Complete duplicated	Scaffold3672	227057	235815	638.1	890	ABC transporter B family member 28
131874at33090	Complete duplicated	Scaffold2722	11638	16571	673.4	936	ABC transporter B family member 28
131914at33090	Complete single-copy	Scaffold4423	62128	71297	295.2	218	Aspartate/glutamate/uridylate kinase
133112at33090	Complete single-copy	Scaffold4559	20100	28018	337.2	252	predicted protein
133622at33090	Complete single-copy	Scaffold4421	156966	171227	369	293	OTU domain-containing protein 3 isoform X1
134078at33090	Complete single-copy	Scaffold4498	470206	481076	464.2	271	Pseudouridine-5'-phosphate glycosidase
135419at33090	Complete single-copy	Scaffold4648	231974	233457	498.8	283	NAD(P)-binding domain
135892at33090	Complete single-copy	Scaffold4361	23258	34394	719.9	421	sphingosine-1-phosphate lyase
136118at33090	Complete single-copy	Scaffold2229	81993	86975	796.3	455	WD40-repeat-containing domain
136267at33090	Complete single-copy	Scaffold519	68152	79325	313.1	249	Leucine-rich repeat, cysteine-containing subtype
136579at33090	Complete single-copy	Scaffold2653	10009	17869	243.6	267	HI0933-like insert domain superfamily
137018at33090	Complete single-copy	Scaffold3049	14567	24059	547.1	286	photosystem II stability/assembly factor HCF136, chloroplastic
137771at33090	Complete single-copy	Scaffold563	494519	511075	925	572	kelch domain-containing protein 4
137787at33090	Complete single-copy	Scaffold2786	258236	270371	583.6	475	U3 small nucleolar RNA-associated protein 6
138499at33090	Complete single-copy	Scaffold4456	297286	311631	247	156	Tetratricopeptide repeat
138782at33090	Complete single-copy	Scaffold259	537246	542282	436.1	251	predicted protein

139062at33090	Complete single-copy	Scaffold983	47386	59382	365.2	260	SWR1 complex subunit 2
139450at33090	Complete single-copy	Scaffold4854	110825	114138	87.6	234	Leucine-rich repeat
139497at33090	Complete duplicated	Scaffold4518	14121	47220	1073.3	882	structural maintenance of chromosomes protein 5
139497at33090	Complete duplicated	Scaffold4310	78707	107800	1056.1	821	structural maintenance of chromosomes protein 5
140683at33090	Complete duplicated	Scaffold380	211129	214318	205.5	183	Glycerol kinase
140683at33090	Complete duplicated	Scaffold1195	916	4539	205.8	183	Glycerol kinase
140876at33090	Complete single-copy	Scaffold2233	49639	60093	363.2	223	psbP domain-containing protein 5, chloroplastic
141370at33090	Complete single-copy	Scaffold3225	213098	221512	363.8	211	Integral membrane protein TerC
141861at33090	Complete single-copy	Scaffold25	197697	201201	214.4	146	Alpha-ketoglutarate-dependent dioxygenase AlkB-like
142077at33090	Complete duplicated	Scaffold4762	13911	21497	209.3	203	Chlorophyll a-b binding protein, chloroplastic
142077at33090	Complete duplicated	Scaffold4632	193239	201846	211.6	208	Chlorophyll a-b binding protein, chloroplastic
142077at33090	Complete duplicated	Scaffold4632	218804	219856	215.7	212	Chlorophyll a-b binding protein, chloroplastic
142077at33090	Complete duplicated	Scaffold4632	184583	185526	215.1	204	Chlorophyll a-b binding protein, chloroplastic
142077at33090	Complete duplicated	Scaffold4632	220281	221426	215.6	208	Chlorophyll a-b binding protein, chloroplastic
142077at33090	Complete duplicated	Scaffold1378	79259	80231	214.4	204	Chlorophyll a-b binding protein, chloroplastic
142382at33090	Complete single-copy	Scaffold1742	54365	60457	244.1	168	PDZ domain
142433at33090	Complete single-copy	Scaffold4431	459737	468056	287.4	218	tRNA/rRNA methyltransferase, SpoU
143102at33090	Complete single-copy	Scaffold607	123756	131280	512	289	WAT1-related protein At3g02690, chloroplastic
143347at33090	Complete single-copy	Scaffold1010	43892	49772	365.4	227	Tetrapyrrole methylase
143963at33090	Complete single-copy	Scaffold1148	77365	88560	257.1	187	predicted protein
143978at33090	Complete single-copy	Scaffold4441	230665	278830	1140	903	protein CTR9 homolog
144144at33090	Missing						
144680at33090	Complete single-copy	Scaffold4721	87645	89000	313.6	252	Ankyrin repeat-containing domain
144716at33090	Complete single-copy	Scaffold4414	104775	111612	345.1	214	CAAX amino terminal protease
144765at33090	Complete single-copy	Scaffold4252	355533	361393	410.4	254	N-glycosylase/DNA lyase OGG1
144990at33090	Complete duplicated	Scaffold2791	19739	23477	400.7	252	NADH dehydrogenase (Ubiquinone) complex I, assembly factor 6
144990at33090	Complete duplicated	Scaffold566	102437	107125	400.7	252	NADH dehydrogenase (Ubiquinone) complex I, assembly factor 6
145061at33090	Complete single-copy	Scaffold1860	103388	107256	788.5	474	protein high chlorophyll fluorescent 107
145734at33090	Complete duplicated	Scaffold4143	1936	4130	421.8	259	alkylated DNA repair protein alkB homolog 8
145734at33090	Complete duplicated	Scaffold3536	69947	75232	464.9	285	alkylated DNA repair protein alkB homolog 8
146354at33090	Complete single-copy	Scaffold4879	481790	482994	283.4	190	thioredoxin-like protein CDSP32, chloroplastic
146469at33090	Complete single-copy	Scaffold4891	18576	24095	340.2	211	delta(3,5)-Delta(2,4)-dienoyl-CoA isomerase, peroxisomal
146772at33090	Complete single-copy	Scaffold3316	50532	53674	393.3	246	Pantoate-beta-alanine ligase
146921at33090	Complete single-copy	Scaffold21	127099	129384	362.4	210	magnesium protoporphyrin IX methyltransferase, chloroplastic
147083at33090	Complete single-copy	Scaffold709	92737	100869	303.7	242	NAD(P)-binding domain superfamily
147262at33090	Complete duplicated	Scaffold4633	16966	20150	391.7	242	protein ABCI12, chloroplastic
147262at33090	Complete duplicated	Scaffold1209	99558	102748	391.7	242	protein ABCI12, chloroplastic
148236at33090	Complete single-copy	Scaffold203	448346	479205	236.1	187	Uncharacterised protein family UPF0454
148539at33090	Complete single-copy	Scaffold4405	60240	72450	568.2	373	tRNA modification GTPase MnmE

148839at33090	Complete single-copy	Scaffold304	264485	274502	478.3	324	enhanced ethylene response protein 5
149782at33090	Complete single-copy	Scaffold1914	44854	48329	235.5	183	Ribosome recycling factor
150470at33090	Complete single-copy	Scaffold4571	222246	239468	333.9	251	protein HGH1 homolog
150559at33090	Complete single-copy	Scaffold4844	196072	199899	266.8	170	ribosomal RNA-processing protein 8
152317at33090	Complete single-copy	Scaffold4467	211642	222957	368.5	237	predicted protein
153820at33090	Complete single-copy	Scaffold1280	90536	117324	525.5	318	uncharacterized protein YNL011C
154870at33090	Fragmented	Scaffold3002	1	2436	321.3	257	nicastrin
155102at33090	Complete duplicated	Scaffold3431	508379	517531	322.8	163	cytochrome c oxidase assembly protein COX11, mitochondrial
155102at33090	Complete duplicated	Scaffold4446	260883	271213	322.9	163	cytochrome c oxidase assembly protein COX11, mitochondrial
156843at33090	Complete single-copy	Scaffold3933	201677	207152	293	183	Ribosomal protein L3
156887at33090	Complete single-copy	Scaffold909	73971	85485	316.9	186	ABC transporter, conserved site
158492at33090	Complete duplicated	Scaffold3437	4866	18901	337	200	Ribosome biogenesis protein
158492at33090	Complete duplicated	Scaffold4779	109009	125539	337	200	Ribosome biogenesis protein
158957at33090	Complete single-copy	Scaffold4080	199468	208037	254.7	159	HAD superfamily
160064at33090	Complete single-copy	Scaffold566	18266	23677	343.6	208	Electron transfer flavoprotein, beta subunit
160610at33090	Complete duplicated	Scaffold755	229	3701	426.8	243	RNA-binding S4 domain
160610at33090	Complete duplicated	Scaffold4631	374284	379550	426.2	243	RNA-binding S4 domain
160680at33090	Complete single-copy	Scaffold4176	116210	131024	820	552	cleavage stimulation factor subunit 77
161054at33090	Complete duplicated	Scaffold337	132235	134459	350.2	253	Release factor glutamine methyltransferase
161054at33090	Complete duplicated	Scaffold4771	222296	224520	350.2	253	Release factor glutamine methyltransferase
161066at33090	Complete single-copy	Scaffold4750	142737	144990	394.4	289	putative oxidoreductase TDA3
161269at33090	Complete single-copy	Scaffold121	78421	84044	332.8	160	deoxycytidylate deaminase
161309at33090	Complete single-copy	Scaffold1465	71135	83061	319.3	245	Calycin
161559at33090	Complete single-copy	Scaffold4382	247545	265901	251.3	200	protein farnesyltransferase/geranylgeranyltransferase type-1 subunit alpha
161966at33090	Complete single-copy	Scaffold4465	541281	547653	343	205	RNA-binding S4 domain
162794at33090	Complete single-copy	Scaffold536	76523	86064	495.3	265	Putative tRNA (cytidine(32)/guanosine(34)-2'-O)-methyltransferase
163097at33090	Complete single-copy	Scaffold1031	67782	71024	524.2	305	peptidyl-prolyl cis-trans isomerase CYP38, chloroplastic
163701at33090	Complete single-copy	Scaffold348	152875	159751	193.7	149	putative uridine kinase C227.14
163833at33090	Complete single-copy	Scaffold18	50262	64363	659.3	360	tRNA threonylcarbamoyladenosine dehydratase
164019at33090	Complete single-copy	Scaffold4905	141178	148128	271.2	236	Class IV aminotransferase
164280at33090	Complete single-copy	Scaffold4366	87587	97756	271.8	158	pre-mRNA-splicing factor 38
164556at33090	Complete single-copy	Scaffold4201	658507	661018	195	148	Putative rRNA methylase
165103at33090	Complete single-copy	Scaffold2879	80252	84629	397.1	235	Carbon-nitrogen hydrolase
166645at33090	Complete single-copy	Scaffold1656	19638	41164	1277.2	973	Sister chromatid cohesion protein
166660at33090	Complete single-copy	Scaffold3275	254525	262911	247.5	143	ATP-dependent helicase/deoxyribonuclease subunit B
167076at33090	Complete single-copy	Scaffold149	130644	134046	226.5	158	oxidoreductase activity
167217at33090	Complete single-copy	Scaffold4630	326456	330470	426	216	tRNA (guanine-N(7)-)-methyltransferase
167325at33090	Complete single-copy	Scaffold4168	95202	109367	184	176	chromosome-associated kinesin
167809at33090	Complete duplicated	Scaffold2798	400705	401456	214.5	159	Peptidyl-prolyl cis-trans isomerase

167809at33090	Complete duplicated	Scaffold4772	246029	250087	209.6	159	Peptidyl-prolyl cis-trans isomerase
167809at33090	Complete duplicated	Scaffold2798	404235	404978	214	159	Peptidyl-prolyl cis-trans isomerase
167809at33090	Complete duplicated	Scaffold4559	28607	30960	204.4	159	Peptidyl-prolyl cis-trans isomerase
167850at33090	Complete single-copy	Scaffold4465	535162	540319	191.2	160	ubiquitin-like-conjugating enzyme ATG10
167932at33090	Complete single-copy	Scaffold4032	248024	250485	365.4	191	psbP domain-containing protein 4, chloroplastic
168269at33090	Complete single-copy	Scaffold4563	171476	177261	384.8	226	p-loop containing nucleoside triphosphate hydrolase protein
168270at33090	Complete single-copy	Scaffold4527	24	9733	379.4	235	protein TAB2 homolog, chloroplastic
168563at33090	Complete single-copy	Scaffold1083	45251	54442	221.6	172	Tetratricopeptide repeat
169007at33090	Complete single-copy	Scaffold905	624441	629523	259.9	144	tRNA (guanine-N1-)-methyltransferase, N-terminal
170939at33090	Complete single-copy	Scaffold979	34138	40485	619.3	342	ATPase, AAA-type, conserved site
170945at33090	Complete single-copy	Scaffold4569	74097	78000	516	342	DEAD-box ATP-dependent RNA helicase 47, mitochondrial
171195at33090	Complete single-copy	Scaffold149	160838	164079	310.7	193	urease accessory protein F
171627at33090	Complete duplicated	Scaffold3431	486062	488087	186.2	137	protein OPI10 homolog
171627at33090	Complete duplicated	Scaffold4446	249362	251372	187.2	137	protein OPI10 homolog
171708at33090	Complete single-copy	Scaffold4635	239971	248095	286.6	241	protein SEH1
171767at33090	Complete single-copy	Scaffold4291	218347	228697	305	179	psbP domain-containing protein 6, chloroplastic
172000at33090	Complete duplicated	Scaffold4341	170047	180887	465.9	310	ribosomal RNA small subunit methyltransferase H
172000at33090	Complete duplicated	Scaffold3959	497488	506190	461.8	288	ribosomal RNA small subunit methyltransferase H
172000at33090	Complete duplicated	Scaffold3959	517128	525330	452.4	290	ribosomal RNA small subunit methyltransferase H
172535at33090	Complete single-copy	Scaffold1643	26797	39051	656.5	410	Ubiquinone biosynthesis monooxygenase COQ6, mitochondrial
172666at33090	Complete single-copy	Scaffold952	281390	294466	318.2	201	Peptide deformylase
173119at33090	Complete single-copy	Scaffold3568	365839	366966	249.7	188	DNA repair protein XRCC3 homolog
173143at33090	Complete single-copy	Scaffold1578	269589	279487	240.6	155	Nucleoporin, NSP1-like, C-terminal
173477at33090	Complete single-copy	Scaffold449	144315	154327	191.8	155	PITH domain
173581at33090	Complete single-copy	Scaffold136	138170	153543	425.1	259	putative deoxyribonuclease TATDN1
174202at33090	Complete duplicated	Scaffold753	51002	52375	182.8	138	putative RNA methyltransferase At5g10620
174202at33090	Complete duplicated	Scaffold754	36429	37801	182.8	138	putative RNA methyltransferase At5g10620
174213at33090	Complete single-copy	Scaffold43	112122	161311	235.5	172	protein FRA10AC1
174767at33090	Complete single-copy	Scaffold4548	159638	162658	452.1	236	Histidine phosphatase superfamily
175583at33090	Complete single-copy	Scaffold4357	210043	212769	184.5	131	probable plastid-lipid-associated protein 11
175792at33090	Complete single-copy	Scaffold1148	74520	77158	268.3	184	homologous-pairing protein 2 homolog
175963at33090	Complete duplicated	Scaffold1555	77780	84868	366.5	206	Putative plastid-lipid-associated protein 6, chloroplastic
175963at33090	Complete duplicated	Scaffold339	76138	83020	366.5	206	Putative plastid-lipid-associated protein 6, chloroplastic
176016at33090	Complete single-copy	Scaffold4549	79461	86890	303.2	157	N-terminal acetyltransferase B complex catalytic subunit NAA20
176328at33090	Complete single-copy	Scaffold1248	152021	172301	749.3	489	eukaryotic translation initiation factor 2D
177296at33090	Complete single-copy	Scaffold4561	146477	149172	278.4	163	methyltransferase-like protein 5
177720at33090	Complete single-copy	Scaffold1364	10226	20806	445.7	375	peptide-N(4)-(N-acetyl-beta- glucosaminyl)asparagine amidase
177964at33090	Complete single-copy	Scaffold2527	4986	15653	369.7	195	peptidyl-prolyl cis-trans isomerase FKBP19, chloroplastic
178386at33090	Complete single-copy	Scaffold4619	202194	205964	366.5	229	Glucose-6-phosphate 1-epimerase

179975at33090	Complete single-copy	Scaffold4130	223520	236556	322.5	167	Thylakoid lumenal protein
180408at33090	Complete single-copy	Scaffold2405	314563	320245	219.3	157	protein COFACTOR ASSEMBLY OF COMPLEX C SUBUNIT B CCB1, chloroplastic
180466at33090	Complete single-copy	Scaffold2003	393746	401818	334.4	194	protein SCO1 homolog 1, mitochondrial
180674at33090	Complete single-copy	Scaffold4519	265061	268332	424.6	235	predicted protein
181108at33090	Complete single-copy	Scaffold4652	172176	180275	279	169	probable plastid-lipid-associated protein 8, chloroplastic
182493at33090	Complete duplicated	Scaffold3444	138741	140143	219.8	170	Peptidyl-prolyl cis-trans isomerase
182493at33090	Complete duplicated	Scaffold76	46162	47564	219.8	170	Peptidyl-prolyl cis-trans isomerase
182513at33090	Complete duplicated	Scaffold1645	57645	69058	467.9	283	Protoheme IX farnesyltransferase
182513at33090	Complete duplicated	Scaffold504	1	15601	467.7	302	Protoheme IX farnesyltransferase
182936at33090	Complete single-copy	Scaffold3959	131580	143306	310.2	231	protein EI24 homolog
182952at33090	Complete single-copy	Scaffold4907	532	18211	273.2	198	Transcriptional regulator TACO1-like
183313at33090	Complete single-copy	Scaffold4073	136931	141674	365	226	Protein-ribulosamine 3-kinase, chloroplastic
183396at33090	Complete single-copy	Scaffold501	1906	10492	186.1	110	uncharacterized protein At5g50100, mitochondrial
183719at33090	Fragmented	Scaffold411	36179	48815	325.3	243	Tocopherol cyclase
183931at33090	Complete single-copy	Scaffold385	147213	153537	395.2	267	THO complex subunit 3
184066at33090	Complete single-copy	Scaffold4635	233299	236303	167.7	95	1-acyl-sn-glycerol-3-phosphate acyltransferase
184405at33090	Complete single-copy	Scaffold379	99418	108338	455	324	adenine DNA glycosylase
184776at33090	Complete single-copy	Scaffold795	40642	45763	257.5	158	nifU-like protein 1, chloroplastic
185172at33090	Complete duplicated	Scaffold1198	83558	96232	175.3	111	Molybdopterin synthase catalytic subunit
185172at33090	Complete duplicated	Scaffold4708	80436	94316	175.3	111	Molybdopterin synthase catalytic subunit
185214at33090	Complete single-copy	Scaffold8	155326	170440	213.3	177	probable plastid-lipid-associated protein 10, chloroplastic
185696at33090	Complete single-copy	Scaffold186	158101	180584	671.3	464	transcription factor 25
185941at33090	Complete single-copy	Scaffold765	71499	74193	212.4	146	psbP domain-containing protein 3, chloroplastic
186340at33090	Complete single-copy	Scaffold2087	27612	31215	336.9	214	psbP domain-containing protein 1, chloroplastic
187462at33090	Complete single-copy	Scaffold4424	133006	146990	519.4	380	Cyclophilin-type peptidyl-prolyl cis-trans isomerase
187866at33090	Complete duplicated	Scaffold421	165152	167355	203.5	158	DTW domain-containing protein 2
187866at33090	Complete duplicated	Scaffold1397	57188	64173	203.5	158	DTW domain-containing protein 2
187914at33090	Complete single-copy	Scaffold2246	33759	46038	480.5	356	Folylpolyglutamate synthetase
188180at33090	Complete single-copy	Scaffold4775	145196	161902	281.5	144	Lactoylglutathione lyase
188436at33090	Complete single-copy	Scaffold2346	142482	159104	295	190	protein Mpv17
188583at33090	Complete single-copy	Scaffold1370	31948	47507	403.1	270	DNA excision repair protein ERCC-1
188938at33090	Complete single-copy	Scaffold4532	49568	63773	337.7	182	Protein of unknown function DUF2854
189891at33090	Complete single-copy	Scaffold829	4257	14441	362.5	209	Tetrapyrrole methylase
190390at33090	Complete duplicated	Scaffold845	97053	111259	278	194	Peroxidase
190390at33090	Complete duplicated	Scaffold1074	79047	92222	277.8	194	Peroxidase
191125at33090	Complete single-copy	Scaffold790	639663	649507	440.4	283	Pseudouridine synthase
191396at33090	Complete single-copy	Scaffold677	86604	106260	417.2	265	tRNA (cytosine(38)-C(5))-methyltransferase isoform X1
191581at33090	Complete duplicated	Scaffold102	252495	255723	399.5	224	UPF0603 protein At1g54780, chloroplastic
191581at33090	Complete duplicated	Scaffold4815	190089	193303	401.1	224	UPF0603 protein At1g54780, chloroplastic

192090at33090	Complete single-copy	Scaffold4375	53445	76945	673.5	458	MIF4G-like domain superfamily
192376at33090	Complete single-copy	Scaffold3725	346514	350180	173.3	109	putative peptidyl-tRNA hydrolase PTRHD1
192456at33090	Complete single-copy	Scaffold4403	166425	174135	225.6	161	OTU domain
192750at33090	Complete single-copy	Scaffold996	453203	467283	281.1	195	ATP-dependent Clp protease proteolytic subunit
193351at33090	Complete single-copy	Scaffold597	281605	283874	244.2	133	DCC family protein At1g52590, chloroplastic
193437at33090	Complete single-copy	Scaffold4404	123222	128731	211.3	131	Molybdopterin cofactor biosynthesis C (MoaC) domain
193683at33090	Complete single-copy	Scaffold4745	209665	217186	195.7	150	multiple myeloma tumor-associated protein 2 homolog
193777at33090	Complete single-copy	Scaffold4866	79440	87411	443.3	250	Peptidase M41-like
193982at33090	Complete single-copy	Scaffold753	99346	110879	232.3	154	Ubiquitin domain
194002at33090	Complete single-copy	Scaffold4734	49007	53019	247.7	143	Impact, N-terminal
194293at33090	Complete single-copy	Scaffold4073	155869	161431	289.7	225	rRNA adenine N(6)-methyltransferase
194372at33090	Complete single-copy	Scaffold233	90214	93561	189	101	uncharacterized protein ycf20
194467at33090	Complete single-copy	Scaffold4471	89993	93504	325.4	180	Protein of unknown function DUF4079
195185at33090	Complete single-copy	Scaffold1141	76426	79310	364.1	208	50S ribosomal protein L4, chloroplastic
195354at33090	Complete single-copy	Scaffold1956	514818	519191	226.7	157	DNA binding protein
195527at33090	Complete single-copy	Scaffold2220	1600	15411	463.8	321	tRNA pseudouridine synthase
195843at33090	Complete duplicated	Scaffold457	15040	23971	280.7	177	Meiotic nuclear division protein 1 homolog
195843at33090	Complete duplicated	Scaffold1758	72433	78058	281.5	177	Meiotic nuclear division protein 1 homolog
196198at33090	Complete single-copy	Scaffold4300	99425	101687	50.6	77	thioredoxin-like protein CITRX, chloroplastic
196248at33090	Complete single-copy	Scaffold1830	64989	65822	181.5	122	Transcription termination factor, mitochondrial/chloroplastic
196324at33090	Complete single-copy	Scaffold4513	226863	231159	302.8	208	Alpha/Beta hydrolase fold
196325at33090	Complete single-copy	Scaffold4849	74769	79292	286.4	240	WD40-repeat-containing domain
196410at33090	Complete single-copy	Scaffold4404	305665	330203	300.4	214	alpha N-terminal protein methyltransferase 1
196443at33090	Complete single-copy	Scaffold4564	299969	307026	125.7	112	thylakoid lumenal 15 kDa protein 1, chloroplastic
196490at33090	Complete single-copy	Scaffold4276	89279	103516	299.7	213	YaeB-like, N-terminal domain
196512at33090	Complete single-copy	Scaffold4418	59605	65165	590.7	355	Ribosome biogenesis GTPase RsgA
196850at33090	Complete single-copy	Scaffold1696	51795	57246	219.8	130	Ribosome-binding factor A
196873at33090	Complete single-copy	Scaffold2405	290483	295517	295.2	149	N-alpha-acetyltransferase MAK3
196991at33090	Complete single-copy	Scaffold4520	542281	546294	213.2	186	G patch domain-containing protein 11
197780at33090	Complete single-copy	Scaffold4735	195747	200017	339.2	182	tRNA (Guanosine(18)-2'-O)-methyltransferase
197838at33090	Complete duplicated	Scaffold1847	22314	24160	232.4	134	Tonoplast intrinsic protein, alpha (Alpha-TIP)
197838at33090	Complete duplicated	Scaffold1324	31855	34153	232.4	134	Tonoplast intrinsic protein, alpha (Alpha-TIP)
198012at33090	Complete duplicated	Scaffold4526	288671	293127	181.6	121	D-aminoacyl-tRNA deacylase
198012at33090	Complete duplicated	Scaffold3771	1435	5881	181.6	121	D-aminoacyl-tRNA deacylase
198570at33090	Complete single-copy	Scaffold3615	107905	122601	455.5	274	F-box protein 7
199163at33090	Complete single-copy	Scaffold330	168515	177205	230.6	196	Nucleic acid-binding, OB-fold
199618at33090	Complete single-copy	Scaffold236	33867	38121	474.9	325	tRNA (guanine(26)-N(2))-dimethyltransferase
199795at33090	Complete single-copy	Scaffold1446	156460	159783	252.1	133	thylakoid lumenal 15.0 kDa protein 2, chloroplastic
200316at33090	Complete single-copy	Scaffold4409	330452	341439	415	294	Cyclophilin-type peptidyl-prolyl cis-trans isomerase

200488at33090	Complete single-copy	Scaffold4430	81741	125003	319.3	208	DNA polymerase I
200499at33090	Complete single-copy	Scaffold4595	264252	270405	523.8	323	PPM-type phosphatase domain
200687at33090	Complete single-copy	Scaffold110	36333	38139	340.6	181	thylakoid lumenal 17.4 kDa protein, chloroplastic
201861at33090	Complete single-copy	Scaffold1191	428927	438248	383	262	Ubiquinol-cytochrome c chaperone, CBP3
202025at33090	Complete single-copy	Scaffold4751	312312	316484	228.8	155	predicted protein
203023at33090	Complete duplicated	Scaffold4589	126636	129632	234.3	146	Rubredoxin
203023at33090	Complete duplicated	Scaffold1121	146927	149923	234.3	146	Rubredoxin
203383at33090	Complete single-copy	Scaffold3567	178985	183991	360.2	246	protein RTF2 homolog
203818at33090	Complete duplicated	Scaffold210	85261	86571	194.9	124	nucleolar protein 16
203818at33090	Complete duplicated	Scaffold211	82931	85074	169.1	118	nucleolar protein 16
203818at33090	Complete duplicated	Scaffold210	83001	85140	169.1	118	nucleolar protein 16
204117at33090	Complete single-copy	Scaffold905	361109	364904	234	169	predicted protein
204880at33090	Complete single-copy	Scaffold4502	150952	160742	369.5	211	Protein of unknown function DUF2301
205308at33090	Complete single-copy	Scaffold4640	209695	216225	368.4	230	RNA exonuclease 4
206164at33090	Complete single-copy	Scaffold4645	96538	107422	261.7	142	PUA domain
206170at33090	Complete single-copy	Scaffold1446	425738	432115	281.1	155	uncharacterized protein LOC103413686
207179at33090	Complete single-copy	Scaffold4541	73743	83057	491.6	306	Peroxisome biogenesis protein 12
207898at33090	Complete single-copy	Scaffold4360	18340	25647	233.6	158	Ribosomal protein L9
208706at33090	Complete single-copy	Scaffold4549	164662	171177	182.8	137	ribosome-recycling factor, chloroplastic
208707at33090	Complete duplicated	Scaffold4107	224832	229044	64.8	70	predicted protein
208707at33090	Complete duplicated	Scaffold1245	49790	54024	61.2	69	predicted protein
208707at33090	Complete duplicated	Scaffold1245	54140	55832	66.5	53	predicted protein
208849at33090	Complete duplicated	Scaffold1074	3154	5401	113.3	70	LSM domain, eukaryotic/archaea-type
208849at33090	Complete duplicated	Scaffold3161	20528	22791	113.3	70	LSM domain, eukaryotic/archaea-type
209015at33090	Complete single-copy	Scaffold4810	24143	35282	324.1	239	Nuclear cap-binding protein subunit 2
209301at33090	Complete single-copy	Scaffold1571	4835	11571	329.3	258	tetratricopeptide repeat protein 4 homolog
209302at33090	Complete single-copy	Scaffold1578	169596	176805	324.3	186	probable mitochondrial import inner membrane translocase subunit TIM21
209516at33090	Complete single-copy	Scaffold374	84893	86917	316.8	179	peptidyl-prolyl cis-trans isomerase FKBP20-2, chloroplastic
210493at33090	Complete duplicated	Scaffold556	51104	70147	181.5	136	protein canopy-1
210493at33090	Complete duplicated	Scaffold67	117333	136373	181.5	136	protein canopy-1
210551at33090	Complete duplicated	Scaffold132	152858	154762	318.9	183	thioredoxin-like protein HCF164, chloroplastic
210551at33090	Complete duplicated	Scaffold4122	324728	329094	318.8	183	thioredoxin-like protein HCF164, chloroplastic
210557at33090	Complete single-copy	Scaffold4420	665798	671846	164.4	127	Protein of unknown function DUF3054
210851at33090	Complete single-copy	Scaffold2673	737443	740511	257.4	128	Glyoxalase/Bleomycin resistance protein/Dihydroxybiphenyl dioxygenase
211645at33090	Complete single-copy	Scaffold2612_2	113347	119985	360.5	218	Ribosomal RNA small subunit methyltransferase G
212331at33090	Complete single-copy	Scaffold4498	301928	310922	465.9	366	nuclear pore complex protein NUP54
212845at33090	Complete single-copy	Scaffold206	60277	62791	249.5	169	psbP domain-containing protein 7, chloroplastic
213827at33090	Complete single-copy	Scaffold4597	252045	277413	238.1	164	Peptidylprolyl isomerase
213828at33090	Complete duplicated	Scaffold1992	68631	72781	217.2	124	NAD(P)-linked oxidoreductase superfamily protein

213828at33090	Complete duplicated	Scaffold4660	82953	87103	217.2	124	NAD(P)-linked oxidoreductase superfamily protein
214313at33090	Complete single-copy	Scaffold7	56026	61624	107.6	120	UPF0690 protein C1orf52 homolog
215062at33090	Complete duplicated	Scaffold1197	10747	15281	219.1	142	N-acetyltransferase 9-like protein
215062at33090	Complete duplicated	Scaffold1833	10747	15281	219.1	142	N-acetyltransferase 9-like protein
215487at33090	Complete single-copy	Scaffold4161	144860	149870	103.1	91	recQ-mediated genome instability protein 2
215817at33090	Complete single-copy	Scaffold1379	77006	88339	411	309	RNA recognition motif domain
216228at33090	Complete single-copy	Scaffold2879	392157	399570	257.2	161	Protein of unknown function DUF1997
216601at33090	Complete single-copy	Scaffold2058	36715	38314	204.4	145	Pyridoxamine 5'-phosphate oxidase, Alr4036 family, FMN-binding domain
217014at33090	Complete single-copy	Scaffold4475	63626	80066	325.7	210	protein COFACTOR ASSEMBLY OF COMPLEX C SUBUNIT B CCB4, chloroplastic
217521at33090	Complete single-copy	Scaffold863	43172	50607	169.4	131	cardiolipin synthase (CMP-forming), mitochondrial
218947at33090	Complete single-copy	Scaffold4784	140531	145215	193	108	predicted protein
219128at33090	Complete single-copy	Scaffold862	103361	108189	124.3	72	predicted protein
219839at33090	Fragmented	Scaffold2232	26772	29191	95.8	56	Ribosomal protein L18
220165at33090	Complete single-copy	Scaffold1945	410071	414711	267.6	180	Dimethylallyl, adenosine tRNA methylthiotransferase
220316at33090	Complete single-copy	Scaffold759	90770	105117	176.3	131	Co-chaperone Hsc20
220587at33090	Complete single-copy	Scaffold1060	44206	47966	299.6	204	protein OS-9 homolog
220589at33090	Complete single-copy	Scaffold2390	6666	12380	464	355	Rubisco LSMT, substrate-binding domain
220981at33090	Complete single-copy	Scaffold4895	129353	138302	263.2	215	26S proteasome non-ATPase regulatory subunit 9
223902at33090	Complete single-copy	Scaffold261	148046	157137	195.5	121	protein disulfide-isomerase 5-1
225336at33090	Complete single-copy	Scaffold1157	130084	139017	200.7	140	predicted protein
227848at33090	Complete single-copy	Scaffold458	33330	41427	270.4	189	RWD domain-containing protein 1
228201at33090	Complete duplicated	Scaffold2104	30091	31347	349.8	249	predicted protein
228201at33090	Complete duplicated	Scaffold2105	30083	31339	349.8	249	predicted protein
229764at33090	Complete duplicated	Scaffold4654	27037	37356	460.5	290	probable inactive shikimate kinase like 2, chloroplastic
229764at33090	Complete duplicated	Scaffold1423	2126	12451	460.5	290	probable inactive shikimate kinase like 2, chloroplastic
229778at33090	Complete single-copy	Scaffold994	140321	141546	148.6	105	predicted protein
229782at33090	Complete single-copy	Scaffold4872	163613	166279	123.2	58	UPF0161 protein At3g09310
230103at33090	Complete single-copy	Scaffold3171	554277	568583	474.2	309	28S ribosomal protein S29, mitochondrial
231151at33090	Complete single-copy	Scaffold4486	88870	94057	219.2	190	NAD(P)-binding domain
231707at33090	Complete single-copy	Scaffold4338	61714	70118	350.3	213	spindle and kinetochore-associated protein 1 homolog
233744at33090	Complete single-copy	Scaffold2758	812	3471	163.5	111	protein Iojap-related, mitochondrial
234046at33090	Complete single-copy	Scaffold1161	102047	106994	131.3	94	prefoldin subunit 1
238204at33090	Complete single-copy	Scaffold4366	273882	275223	129.5	74	predicted protein
238385at33090	Complete duplicated	Scaffold2613	41900	42799	117.6	70	photosystem I reaction center subunit N
238385at33090	Complete duplicated	Scaffold4048	722902	725236	117.6	70	photosystem I reaction center subunit N
238385at33090	Complete duplicated	Scaffold2613	40670	41788	117.6	70	photosystem I reaction center subunit N
238385at33090	Complete duplicated	Scaffold12	197636	201760	101.8	69	photosystem I reaction center subunit N

Supplementary Table S18. Sequence similarity between duplicated gene fractions in scaffolds

BUSCO ID	Scaffold containing duplicated BUSCO fraction: Scaffold A	Category (A)	Score	Length (aa)	Scaffold B	Category (A)	Score	Length (aa)	Similarity between duplicated BUSCO fractions (all-by-all comparison[1]): Gene fraction in Scaffold A: Start position (bp)	End position (bp)	Gene fraction in Scaffold B: Start position (bp)	End position (bp)	Comparison between A and B: Coverage (B/A) (%)	Identity (%) in aligned region	*E*-value	Search for other redundant genes (no duplicated BUSCOs) (all-by-all comparison): Scaffold A: Gene ID	Scaffold B: Gene ID	Comparison between A and B: Identity (%)	*E*-value	Gene coverage (%) in Scaffold A	Gene coverage (%) in Scaffold B	Redundancy (Yes or No)
106886at33090	Scaffold4476	Duplicated	346.7	395	Scaffold736	Duplicated	346.7	395	1	395	1	395	100	100	0	PGJG327700	PGJG094950	99.7	0	100	100	No
111249at33090	Scaffold1273	Duplicated	380.7	414	Scaffold2556	Duplicated	380.6	420	52	414	58	420	100	100	0	PGJG147150	PGJG229300	82.5	0	94	100	No
112238at33090	Scaffold182	Duplicated	774	849	Scaffold19	Duplicated	775	731	148	849	30	731	100	99.9	0	PGJG027520	PGJG003920	100	0	96.4	100	No
112542at33090	Scaffold1462	Duplicated	457.7	293	Scaffold2954	Duplicated	457.7	293	1	293	1	293	100	100	0	PGJG161170	PGJG246570	99.1	0	100	100	No
115992at33090	Scaffold1110	Duplicated	409.1	301	Scaffold227	Duplicated	409.1	298	4	301	1	298	100	100	0	PGJG132220	PGJG033770	82.4	1E-132	100	100	No
116411at33090	Scaffold1399	Duplicated	164.1	490	Scaffold68	Duplicated	177.6	442	84	477	46	396	112.3	55.1	3E-135	PGJG156840	PGJG011080	66.1	5E-170	80	87.8	No
120589at33090	Scaffold1719	Duplicated	662	544	Scaffold4840	Duplicated	639	555	1	535	1	535	100	97.4	0	PGJG179240	PGJG394340	100	0	67.5	98.9	No
126427at33090	Scaffold1789	Duplicated	401.7	320	Scaffold191	Duplicated	401.7	320	1	320	1	320	100	100	0	NA						No
	Scaffold1789	Duplicated	401.7	320	Scaffold4518	Duplicated	401.7	320	1	320	1	320	100	100	0	PGJG183710	PGJG336390	99.1	0	100	100	No
	Scaffold191	Duplicated	401.7	320	Scaffold4518	Duplicated	401.7	320	1	320	1	320	100	100	0	NA						No
130268at33090	Scaffold3448	Duplicated	407.9	341	Scaffold4055	Duplicated	407.2	341	1	341	1	341	100	98.5	0	PGJG265070	PGJG283690	97.5	8E-178	71.1	78.9	No
131556at33090	Scaffold753	Duplicated	504.1	501	Scaffold754	Duplicated	504.1	760	1	312	1	312	100	99.7	0	PGJG096650	PGJG096770	87.9	0	100	100	No
131874at33090	Scaffold2722	Duplicated	681	1256	Scaffold3672	Duplicated	638.1	1373	8	868	12	873	99.9	40.8	0	PGJG236970	PGJG272360	45.9	0	98.4	98.6	No
139497at33090	Scaffold4310	Duplicated	1056.1	1059	Scaffold4518	Duplicated	1073.3	1051	1	1059	1	1051	100.8	85.8	0	PGJG296040	PGJG336300	82	0	86.4	72.5	No
140683at33090	Scaffold1195	Duplicated	205.8	441	Scaffold380	Duplicated	205.5	452	1	441	12	452	100	100	0	PGJG140790	PGJG052980	100	0	90.1	96	No
142077at33090	Scaffold4632	Duplicated	211.6<br>215.7<br>215.1<br>215.6	270<br>270<br>270<br>975	Scaffold4762	Duplicated	209.3	2187	1	270	1	270	100	100	0	NA						No
	Scaffold1378	Duplicated	214.4	273	Scaffold4762	Duplicated	209.3	2187	12	273	12	270	101.2	92.8	3E-175	NA						No
	Scaffold1378	Duplicated	214.4	273	Scaffold4632	Duplicated	211.6<br>215.7<br>215.1<br>215.6	270<br>270<br>270<br>975	1	273	1	270	101.1	92.4	0	PGJG155250	PGJG358150	92.4	0	100	100	No
144990at33090	Scaffold2791	Duplicated	400.7	303	Scaffold566	Duplicated	400.7	303	1	303	1	303	100	100	0	PGJG239660	PGJG076600	98.8	0	100	100	No
145734at33090	Scaffold3536	Duplicated	464.9	350	Scaffold4143	Duplicated	421.8	322	1	322	1	322	100	100	0	PGJG267250	PGJG287350	100	1E-55	72.9	23.2	No
147262at33090	Scaffold1209	Duplicated	391.7	384	Scaffold4633	Duplicated	391.7	384	1	384	1	384	100	100	0	NA						No
155102at33090	Scaffold3431	Duplicated	322.8	282	Scaffold4446	Duplicated	322.9	282	1	282	1	282	100	98.9	0	PGJG264210	PGJG320610	99.5	1E-139	100	100	No
158492at33090	Scaffold3437	Duplicated	337	270	Scaffold4779	Duplicated	337	270	1	270	1	270	100	100	0	PGJG264730	PGJG384540	90.5	2E-164	86	76	No
160610at33090	Scaffold4631	Duplicated	426.2	391	Scaffold755	Duplicated	426.8	334	103	391	37	334	97	93	0	PGJG357760	PGJG096790	100	0	99.4	37.2	No
161054at33090	Scaffold337	Duplicated	350.2	365	Scaffold4771	Duplicated	350.2	365	1	365	1	365	100	100	0	PGJG047240	PGJG383270	99.8	0	100	100	No
16726at33090	Scaffold34	Duplicated	531.5	808	Scaffold4773	Duplicated	542.4	805	1	808	1	805	100.4	86.5	0	PGJG005960	PGJG383600	86.5	0	100	100	No
	Scaffold1929	Duplicated	530.2	673	Scaffold4773	Duplicated	542.4	805	1	673	136	805	100.4	87.7	0	PGJG192080	PGJG383600	87.8	0	100	82.3	No
	Scaffold1929	Duplicated	530.2	673	Scaffold34	Duplicated	531.5	808	1	673	136	808	100	100	0	PGJG192080	PGJG005960	100	0	100	82.3	No
167809at33090	Scaffold4559	Duplicated	204.4	174	Scaffold4772	Duplicated	209.6	172	1	171	1	171	100	77.2	1E-100	PGJG344090	PGJG383420	77.2	1E-99	97.7	98.8	No
	Scaffold2798	Duplicated	214.5	171	Scaffold4772	Duplicated	209.6	172	1	171	1	171	100	87.7	2E-114	PGJG240510	PGJG383420	87.1	8E-112	99.4	98.8	No
	Scaffold2798	Duplicated	214.5	171	Scaffold4559	Duplicated	204.4	174	1	171	1	171	100	77.8	6E-102	PGJG240510	PGJG344090	76	2E-99	99.4	97.7	No
171627at33090	Scaffold3431	Duplicated	186.2	187	Scaffold4446	Duplicated	187.2	187	1	187	1	187	100	99.5	7E-141	PGJG264210	PGJG320610	99.5	1E-139	100	100	No
172000at33090	Scaffold3959	Duplicated	461.8<br>452.4	474<br>424	Scaffold4341	Duplicated	465.9	475	62	474	39	475	94.5	92	0	PGJG279630	PGJG298340	80.7	3E-43	95.4	94.3	No
174202at33090	Scaffold753	Duplicated	182.8	188	Scaffold754	Duplicated	182.8	188	1	188	1	188	100	100	7E-145	PGJG096650	PGJG096770	87.9	0	100	100	No
175963at33090	Scaffold1555	Duplicated	366.5	287	Scaffold339	Duplicated	366.5	287	1	287	1	287	100	99.7	0	PGJG167900	PGJG047430	99	1E-139	100	67.7	No
182493at33090	Scaffold3444	Duplicated	219.8	392	Scaffold76	Duplicated	219.8	392	1	392	1	392	100	100	0	PGJG264940	PGJG012480	82.6	8E-154	97.8	74.7	No
182513at33090	Scaffold1645	Duplicated	467.9	451	Scaffold504	Duplicated	467.7	495	1	439	1	462	95	83.5	0	PGJG174010	PGJG068080	100	2E-175	53.3	67.5	No
185172at33090	Scaffold1198	Duplicated	175.3	197	Scaffold4708	Duplicated	175.3	197	1	197	1	197	100	100	2E-151	NA						No
187866at33090	Scaffold1397	Duplicated	203.5	295	Scaffold421	Duplicated	203.5	295	1	295	1	295	100	100	0	PGJG156550	PGJG057960	84.4	0	100	100	No
190390at33090	Scaffold1074	Duplicated	277.8	328	Scaffold845	Duplicated	278	328	1	328	1	328	100	98.8	0	PGJG129350	PGJG106720	78.1	3E-113	70.3	68.7	No
191581at33090	Scaffold102	Duplicated	399.5	298	Scaffold4815	Duplicated	401.1	298	1	298	1	298	100	99.7	0	PGJG015550	PGJG391010	99.7	0	100	100	No
195843at33090	Scaffold1758	Duplicated	281.5	299	Scaffold457	Duplicated	280.7	365	1	298	1	280	106.5	93	0	PGJG181990	PGJG062580	98.7	0	100	100	No
197838at33090	Scaffold1324	Duplicated	232.4	185	Scaffold1847	Duplicated	232.4	185	1	185	1	185	100	100	2E-141	NA						No

198012at33090	Scaffold3771	Duplicated	181.6	195	Scaffold4526	Duplicated	181.6	195	1	195	1	195	100	100	3E-149	NA						No
203023at33090	Scaffold1121	Duplicated	234.3	210	Scaffold4589	Duplicated	234.3	210	1	210	1	210	100	100	5E-158	NA						No
203818at33090	Scaffold210	Duplicated	194.9	190	Scaffold211	Duplicated	169.1	190	1	190	1	190	100	100	7E-143	PGJG030920	PGJG031050	98.4	1.00E-178	98.8	80.1	No
208707at33090	Scaffold1245	Duplicated	61.2<br>66.5	204<br>160	Scaffold4107	Duplicated	64.8	161	114	204	71	161	100	82.4	6E-54	PGJG144970	PGJG285330	98.7	1.00E-54	23.2	98.7	No
208849at33090	Scaffold1074	Duplicated	113.3	119	Scaffold3161	Duplicated	113.3	119	1	119	1	119	100	100	9E-90	NA						No
210493at33090	Scaffold556	Duplicated	181.5	260	Scaffold67	Duplicated	181.5	260	1	260	1	260	100	100	0	PGJG074520	PGJG010840	100	0	98.3	100	No
210551at33090	Scaffold132	Duplicated	318.9	216	Scaffold4122	Duplicated	318.8	401	15	216	200	401	100	99.5	2E-150	PGJG020020	PGJG285920	95.8	0	99.5	97.4	No
213828at33090	Scaffold1992	Duplicated	217.2	194	Scaffold4660	Duplicated	217.2	194	1	194	1	194	100	100	2E-145	PGJG197510	PGJG362800	99.2	4E-86	71.1	41.7	No
215062at33090	Scaffold1197	Duplicated	219.1	195	Scaffold1833	Duplicated	219.1	195	1	195	1	195	100	100	3E-149	PGJG140990	PGJG186410	59.2	0	100	100	No
22618at33090	Scaffold3153	Duplicated	909.7	848	Scaffold3154	Duplicated	909.7	848	1	848	1	848	100	100	0	PGJG253800	PGJG253830	100	0	100	100	Yes
228201at33090	Scaffold2104	Duplicated	349.8	316	Scaffold2105	Duplicated	349.8	316	1	316	1	316	100	100	0	PGJG203460	PGJG203510	99.2	0	100	100	No
229764at33090	Scaffold1423	Duplicated	460.5	357	Scaffold4654	Duplicated	460.5	357	1	357	1	357	100	100	0	PGJG158630	PGJG361910	99.5	2E-152	68.8	71.4	No
238385at33090	Scaffold2613	Duplicated	117.6	120	Scaffold4048	Duplicated	117.6	120	1	120	1	120	100	100	9E-87	PGJG232560	PGJG283210	94	3E-161	100	100	No
	Scaffold12	Duplicated	101.8	197	Scaffold4048	Duplicated	117.6	120	132	197	55	120	100	90.9	2E-41	NA						No
	Scaffold12	Duplicated	101.8	197	Scaffold2613	Duplicated	117.6	120	132	197	55	120	100	90.9	2E-41	NA						No
32730at33090	Scaffold1473	Duplicated	705.7	610	Scaffold1823	Duplicated	705.7	610	1	610	1	610	100	100	0	PGJG161850	PGJG185890	88.5	0	99.6	99.8	No
32923at33090	Scaffold1262	Duplicated	1284	785	Scaffold2430	Duplicated	1208.5	710	1	695	1	693	100.3	95.5	0	PGJG146590	PGJG223150	93.8	0	100	100	No
36284at33090	Scaffold4577	Duplicated	1099.7	778	Scaffold70	Duplicated	1103.9	793	1	778	1	793	98.1	98.1	0	PGJG347600	PGJG011550	99.8	0	100	100	No
39913at33090	Scaffold1963	Duplicated	1060	740	Scaffold1964	Duplicated	1060	740	1	740	1	740	100	100	0	PGJG195000	PGJG195030	94.8	0	100	100	No
49092at33090	Scaffold2201	Duplicated	960.2	834	Scaffold2284	Duplicated	844.2	544	1	530	1	530	100	99.8	0	PGJG209110	PGJG214000	99.8	0	98.4	100	No
52348at33090	Scaffold1097	Duplicated	600.1	464	Scaffold2813	Duplicated	600.1	464	1	464	1	464	100	100	0	PGJG131150	PGJG240990	80.1	7E-174	62.6	94.3	No
52795at33090	Scaffold4351	Duplicated	337.8	667	Scaffold970	Duplicated	337.8	667	1	667	1	667	100	100	0	PGJG299590	PGJG120020	99.5	0	100	100	No
67389at33090	Scaffold1092	Duplicated	523.3	509	Scaffold1766	Duplicated	524.9	510	1	509	1	510	99.8	97.5	0	PGJG130870	PGJG182260	93.4	0	85.9	75.3	No
69614at33090	Scaffold1882	Duplicated	561.4	461	Scaffold2771	Duplicated	561.4	461	1	461	1	461	100	100	0	NA						No
72992at33090	Scaffold4473	Duplicated	627	649	Scaffold4537	Duplicated	627	649	1	649	1	649	100	100	0	PGJG327050	PGJG339890	100	4E-63	98	8.1	No
74742at33090	Scaffold1699	Duplicated	350.7	452	Scaffold821	Duplicated	350.7	452	1	452	1	452	100	100	0	PGJG177680	PGJG104570	99.9	0	100	100	No
76849at33090	Scaffold4566	Duplicated	723.3	593	Scaffold942	Duplicated	723.3	593	1	593	1	593	100	100	0	PGJG345380	PGJG117570	100	0	98.5	94.1	No
88687at33090	Scaffold1772	Duplicated	477.8	531	Scaffold957	Duplicated	493.2	489	1	531	1	489	108.6	91.9	0	PGJG182660	PGJG118860	99.1	0	99.5	77.8	No
90240at33090	Scaffold3713	Duplicated	515.4	456	Scaffold4897	Duplicated	515.6	448	26	438	35	447	100	100	0	PGJG273900	PGJG402660	100	1E-147	56.2	99.5	No
92579at33090	Scaffold1374	Duplicated	494.3	411	Scaffold414	Duplicated	488.9	431	1	411	1	431	95.3	95.4	0	NA						No
97593at33090	Scaffold1674	Duplicated	441	417	Scaffold4363	Duplicated	441	417	1	417	1	417	100	100	0	PGJG175970	PGJG303210	66.7	9E-124	73.4	92.2	No

[1]For pairwise-comparison of two sequences, BLASTP (version 2.3.0+) were employed with the cut-off *E* -value of 1*E* -4

Supplementary Table S19. The information of BUSCOs analyzed from geneset of *P. grandiflorus*.

BUSCO ID	Status	PG Gene ID	BUSCO Score	Length (bp)	Description
2at33090	Complete single-copy	PGJG260030	5013.9	3977	Midasin
5at33090	Complete single-copy	PGJG156030	3907	2527	sacsin
24at33090	Complete single-copy	PGJG151140	5660	3588	auxin transport protein BIG
255at33090	Complete single-copy	PGJG227860	3519.6	2209	protein ILITYHIA
383at33090	Complete single-copy	PGJG093860	1302.5	879	WD40-repeat-containing domain
1363at33090	Complete single-copy	PGJG011720	1620.1	1138	UDP-glucose
1419at33090	Complete single-copy	PGJG180050	1956.5	1494	E3 ubiquitin-protein ligase listerin
1557at33090	Complete single-copy	PGJG342570	1347.2	994	2-succinyl-5-enolpyruvyl-6-hydroxy-3-cyclohexene-1-carboxylic-acid synthase
1855at33090	Complete single-copy	PGJG123450	2277.5	1681	thyroid adenoma-associated protein homolog
4004at33090	Complete single-copy	PGJG353930	1653.4	1104	splicing factor 3B subunit 3
4034at33090	Fragmented	PGJG128790	822.2	640	transcription initiation factor TFIID subunit 2
4115at33090	Complete single-copy	PGJG371880	1435.7	911	Glycoside hydrolase, family 2
5305at33090	Complete single-copy	PGJG308870	1232.2	825	F-box protein
5927at33090	Complete single-copy	PGJG289690	1286.7	981	Armadillo-type fold
6512at33090	Complete single-copy	PGJG103740	890.1	766	protein timeless homolog isoform X1
6843at33090	Complete single-copy	PGJG227420	952	593	Thioredoxin-like fold
7366at33090	Complete single-copy	PGJG263470	1319.4	809	Zinc finger, CCHC-type
8072at33090	Complete single-copy	PGJG211100	1266.1	814	transportin MOS14
9044at33090	Complete single-copy	PGJG089710	1402.6	869	GHMP kinase, C-terminal domain
9058at33090	Complete single-copy	PGJG307800	1076.3	992	XPG/Rad2 endonuclease
9849at33090	Complete single-copy	PGJG356130	860.5	798	MMS19 nucleotide excision repair protein homolog isoform X1
10924at33090	Complete single-copy	PGJG243420	1179.2	728	Alpha-1,6-glucosidases, pullulanase-type
11664at33090	Complete single-copy	PGJG020110	1084.9	701	DNA topoisomerase, type IIA, subunit A/C-terminal
11800at33090	Complete single-copy	PGJG185620	1936.4	1042	5-oxoprolinase
12439at33090	Complete single-copy	PGJG398630	1239.2	739	WD40-repeat-containing domain
13039at33090	Complete single-copy	PGJG065070	1283.5	703	Aminoacyl-tRNA synthetase, class Ia
13643at33090	Complete single-copy	PGJG120000	1099	710	conserved oligomeric Golgi complex subunit 7
15682at33090	Complete single-copy	PGJG238650	1070	691	nuclear cap-binding protein subunit 1
16726at33090	Complete single-copy	PGJG353410	1047.2	721	ATPase, AAA-type, conserved site
18455at33090	Complete single-copy	PGJG085970	807.4	518	WD40-repeat-containing domain
18801at33090	Complete single-copy	PGJG325530	1120.4	851	condensin complex subunit 3
21163at33090	Complete single-copy	PGJG217080	924.2	548	ribonuclease E/G-like protein, chloroplastic isoform X1
21751at33090	Fragmented	PGJG046650	766	553	glycine--tRNA ligase, chloroplastic/mitochondrial 2 isoform X1
21857at33090	Complete single-copy	PGJG296730	722.7	536	anaphase-promoting complex subunit 4
22109at33090	Complete single-copy	PGJG329930	712.5	431	quinolinate synthase, chloroplastic
22618at33090	Complete single-copy	PGJG253790	935.2	645	BTB/POZ domain-containing protein At2g30600
22958at33090	Complete single-copy	PGJG159530	1058.9	695	SAP domain
23444at33090	Complete single-copy	PGJG385240	578.6	456	pumilio homolog 23
23853at33090	Fragmented	PGJG041080	658.1	476	probable transmembrane GTPase FZO-like, chloroplastic
24838at33090	Complete single-copy	PGJG379110	672.3	634	G patch domain-containing protein, N-terminal
25435at33090	Complete single-copy	PGJG101130	1060.2	769	chromosome transmission fidelity protein 18 homolog
26868at33090	Complete single-copy	PGJG079500	765.3	507	transcription factor bHLH140
27237at33090	Complete single-copy	PGJG084660	826	592	ribonuclease II, chloroplastic/mitochondrial
27607at33090	Complete single-copy	PGJG164140	804.9	543	FG-GAP repeat-containing protein
28920at33090	Complete single-copy	PGJG283350	874.1	544	probable DNA helicase MCM8
31155at33090	Complete single-copy	PGJG363830	1101.2	727	NatC N(Alpha)-terminal acetyltransferase, Mak10 subunit
31855at33090	Complete single-copy	PGJG306120	958.7	526	ABC transporter G family member 7
32730at33090	Complete single-copy	PGJG185890	705.7	434	THO complex subunit 1
32923at33090	Complete duplicated	PGJG223150	1207.8	699	ubiquitin-like modifier-activating enzyme atg7
32923at33090	Complete duplicated	PGJG146590	1219.5	699	ubiquitin-like modifier-activating enzyme atg7
33264at33090	Complete single-copy	PGJG044520	719.9	461	Tyrosyl-DNA phosphodiesterase 1
34517at33090	Complete single-copy	PGJG150110	1015.8	663	conserved oligomeric Golgi complex subunit 5
35499at33090	Fragmented	PGJG374210	648.6	373	FAD/NAD(P)-binding domain
35887at33090	Complete single-copy	PGJG210340	776.6	543	Rad4 beta-hairpin domain 1
36284at33090	Complete duplicated	PGJG347470	1115.4	611	dynamin-like protein ARC5

36284at33090	Complete duplicated	PGJG011520	1089.4	614	dynamin-like protein ARC5
37663at33090	Complete single-copy	PGJG283660	839.4	532	MoeA, C-terminal, domain IV
37699at33090	Complete single-copy	PGJG042500	1118.5	737	DEAD-box ATP-dependent RNA helicase 13
39913at33090	Complete single-copy	PGJG195000	1060	544	4-hydroxy-3-methylbut-2-en-1-yl diphosphate synthase
40630at33090	Complete single-copy	PGJG045060	973.8	645	DEAD-box ATP-dependent RNA helicase 28
41146at33090	Fragmented	PGJG014740	512.4	291	Electron transfer flavoprotein-ubiquinone oxidoreductase, mitochondrial
41244at33090	Complete single-copy	PGJG070010	761.9	435	Imidazole glycerol phosphate synthase hisHF
42407at33090	Complete single-copy	PGJG399900	917.9	641	pre-mRNA-processing protein 40C
43683at33090	Complete single-copy	PGJG216610	805.9	505	PPM-type phosphatase domain
43957at33090	Complete single-copy	PGJG365820	1079	660	WD40 repeat
44063at33090	Complete single-copy	PGJG291530	740.1	429	4-alpha-glucanotransferase
44526at33090	Complete single-copy	PGJG223810	575.3	411	GPI transamidase component PIG-T
46307at33090	Complete single-copy	PGJG285770	841.7	442	methylcrotonoyl-CoA carboxylase beta chain, mitochondrial
48027at33090	Complete single-copy	PGJG123920	804.8	427	Aminoacyl-tRNA synthetase, class I, anticodon-binding
48290at33090	Complete single-copy	PGJG363500	718.1	481	probable Ufm1-specific protease
48645at33090	Complete single-copy	PGJG092070	686.2	487	Cwf19-like, C-terminal domain-1
49092at33090	Complete duplicated	PGJG209110	901.8	460	Aldehyde dehydrogenase
49092at33090	Complete duplicated	PGJG214000	902.1	460	Aldehyde dehydrogenase
50512at33090	Complete single-copy	PGJG154290	360.1	302	Predicted protein
50528at33090	Fragmented	PGJG239020	341	259	Light-mediated development protein DET1
51214at33090	Complete single-copy	PGJG098440	645.3	421	RNA helicase
51262at33090	Complete single-copy	PGJG026590	472.8	406	glyoxysomal processing protease, glyoxysomal
52348at33090	Complete duplicated	PGJG241000	600.1	392	JmjC domain
52348at33090	Complete duplicated	PGJG131180	512.1	343	JmjC domain
52435at33090	Complete single-copy	PGJG156980	935.4	455	Phytoene desaturase
52795at33090	Complete single-copy	PGJG018890	747.4	426	Lysine--tRNA ligase
53416at33090	Complete single-copy	PGJG352850	525.9	359	Alpha-1,3-glucosyltransferase
53527at33090	Complete single-copy	PGJG335280	1253.8	787	4-alpha-glucanotransferase
55448at33090	Complete single-copy	PGJG148500	812.2	420	Prolyl-tRNA synthetase
57004at33090	Complete single-copy	PGJG093070	744.7	444	FAD/NAD(P)-binding domain superfamily
58672at33090	Complete single-copy	PGJG325380	457.7	274	Violaxanthin de-epoxidase
60262at33090	Complete single-copy	PGJG140490	616.7	375	WD40/YVTN repeat-like-containing domain superfamily
62262at33090	Complete single-copy	PGJG116460	407.9	296	putative tRNA pseudouridine synthase Pus10
63411at33090	Complete single-copy	PGJG236490	670	370	fe-S cluster assembly factor HCF101, chloroplastic
63853at33090	Complete single-copy	PGJG300840	644.8	343	UDP-sulfoquinovose synthase, chloroplastic
64272at33090	Complete single-copy	PGJG205780	798.3	659	nucleolar protein 14
65263at33090	Complete single-copy	PGJG173750	475.6	344	cell cycle checkpoint protein RAD17 isoform X1
67132at33090	Complete single-copy	PGJG068070	760.5	416	DNA topoisomerase 6 subunit B
67389at33090	Complete duplicated	PGJG130870	539.2	451	methyltransferase-like protein 17, mitochondrial
67389at33090	Complete duplicated	PGJG182260	535	403	methyltransferase-like protein 17, mitochondrial
68169at33090	Complete single-copy	PGJG315230	813.1	635	nuclear pore complex protein NUP88
68537at33090	Complete single-copy	PGJG168810	528.8	332	Chitobiosyldiphosphodolichol beta-mannosyltransferase
68957at33090	Complete single-copy	PGJG079370	671.8	443	Mannosyltransferase
69073at33090	Complete single-copy	PGJG274160	465.4	294	Protein arginine methyltransferase NDUFAF7
69137at33090	Complete single-copy	PGJG336910	641.2	510	CDK5RAP3-like protein
69505at33090	Complete single-copy	PGJG226820	546.4	389	tRNA A64-2'-O-ribosylphosphate transferase
69614at33090	Complete single-copy	PGJG238770	561.4	352	WD40-repeat-containing domain
70613at33090	Complete single-copy	PGJG359180	714.5	374	homogentisate 1,2-dioxygenase
71620at33090	Complete single-copy	PGJG390170	550.1	355	DNA primase large subunit
72992at33090	Complete single-copy	PGJG339900	625.4	427	RED-like, N-terminal
74230at33090	Complete single-copy	PGJG047180	701.4	375	Acyl-CoA N-acyltransferase
74580at33090	Complete single-copy	PGJG352370	590.7	400	protein DGCR14
74742at33090	Complete single-copy	PGJG104550	350.7	299	histone acetyltransferase type B catalytic subunit
75785at33090	Complete single-copy	PGJG213960	414.2	295	P-loop containing nucleoside triphosphate hydrolase
76026at33090	Complete single-copy	PGJG210050	484.9	332	histone-lysine N-methyltransferase ATXR2
76849at33090	Complete duplicated	PGJG117570	723	457	mini-chromosome maintenance complex-binding protein
76849at33090	Complete duplicated	PGJG345380	723.2	457	mini-chromosome maintenance complex-binding protein
79950at33090	Complete single-copy	PGJG111200	344.3	230	Rhodanese-like domain

83749at33090	Complete single-copy	PGJG278160	608.9	342	3-oxoacyl-
84114at33090	Complete single-copy	PGJG381990	631.1	345	Lysine-tRNA ligase
84264at33090	Complete single-copy	PGJG358120	596.4	617	WW domain
87347at33090	Complete single-copy	PGJG006170	1212.3	744	WD40 repeat
87734at33090	Complete single-copy	PGJG248950	283.8	241	DEAD-box ATP-dependent RNA helicase 58, chloroplastic isoform X1
88295at33090	Complete single-copy	PGJG290590	457.3	317	Rab3-GAP regulatory subunit
88687at33090	Complete duplicated	PGJG118860	490.1	367	cell cycle checkpoint control protein RAD9A
88687at33090	Complete duplicated	PGJG182660	445.5	327	cell cycle checkpoint control protein RAD9A
88771at33090	Complete single-copy	PGJG201340	564.7	390	Tetratricopeptide-like helical domain superfamily
89796at33090	Complete single-copy	PGJG373530	858.4	570	conserved oligomeric Golgi complex subunit 6
90240at33090	Complete single-copy	PGJG273880	521.3	331	PUA domain
90301at33090	Complete single-copy	PGJG340740	683.7	371	Pyridoxal phosphate-dependent transferase
90872at33090	Complete single-copy	PGJG059870	648	338	Cation efflux protein
92418at33090	Complete single-copy	PGJG293200	509.8	268	uncharacterized protein ycf39
92579at33090	Complete single-copy	PGJG056890	494.2	351	rhomboid-like protein 15
95807at33090	Fragmented	PGJG359670	309.1	255	ApaG domain
96953at33090	Complete single-copy	PGJG212260	347.2	284	WD40-repeat-containing domain
97593at33090	Complete single-copy	PGJG175970	441.7	299	Origin of replication complex subunit 4
101241at33090	Complete duplicated	PGJG154430	173	295	actin-related protein 6
101241at33090	Complete duplicated	PGJG164580	175.8	295	actin-related protein 6
101701at33090	Complete single-copy	PGJG033600	795.7	518	UTP--glucose-1-phosphate uridylyltransferase 3, chloroplastic
102307at33090	Complete single-copy	PGJG358480	591.3	293	WD40-repeat-containing domain
103267at33090	Complete single-copy	PGJG299300	339.5	250	recQ-mediated genome instability protein 1
103382at33090	Complete single-copy	PGJG204310	318.1	227	Prolyl 3-hydroxylase 1
104218at33090	Complete single-copy	PGJG305420	544.4	299	cysteine synthase 2
104557at33090	Fragmented	PGJG338650	326	178	Coenzyme F420 hydrogenase/dehydrogenase beta subunit, C-terminal
104821at33090	Complete single-copy	PGJG091530	493.5	388	RNA 2-O ribose methyltransferase, substrate binding
104867at33090	Complete single-copy	PGJG297020	940.5	603	aminodeoxychorismate synthase, chloroplastic
105044at33090	Complete single-copy	PGJG250630	926.2	589	putative DEAD-box ATP-dependent RNA helicase 29
105782at33090	Complete single-copy	PGJG275220	433.8	297	Cobalamin (Vitamin B12) biosynthesis CobW-like,C-terminal
105867at33090	Complete single-copy	PGJG323030	493.1	301	alcohol dehydrogenase
106769at33090	Complete single-copy	PGJG378770	624.9	325	4-diphosphocytidyl-2-C-methyl-D-erythritol kinase, chloroplastic
106886at33090	Complete duplicated	PGJG095090	346.9	263	protein CDC73 homolog
106886at33090	Complete duplicated	PGJG327520	344.2	263	protein CDC73 homolog
107107at33090	Complete single-copy	PGJG278120	477.2	366	Ubiquitin-like domain superfamily
107413at33090	Fragmented	PGJG338640	447.5	281	GPI mannosyltransferase 1
108705at33090	Fragmented	PGJG070740	256.4	197	uncharacterized protein At4g17910 isoform X1
109586at33090	Complete single-copy	PGJG014580	773.3	445	Methionyl-tRNA synthetase
109823at33090	Complete single-copy	PGJG336440	454.1	263	protein LOW PSII ACCUMULATION 3, chloroplastic
110222at33090	Complete duplicated	PGJG010550	495.7	409	DnaJ domain
110222at33090	Complete duplicated	PGJG011770	495.7	409	DnaJ domain
110225at33090	Complete single-copy	PGJG241890	788.1	469	riboflavin biosynthesis protein PYRR, chloroplastic
111210at33090	Complete single-copy	PGJG066710	464.8	271	HAD superfamily
111213at33090	Fragmented	PGJG260810	190	110	epimerase family protein SDR39U1 homolog, chloroplastic
111249at33090	Complete single-copy	PGJG147150	381.6	240	predicted protein
111924at33090	Complete single-copy	PGJG027710	499.6	327	protein arginine N-methyltransferase 2
112238at33090	Complete duplicated	PGJG027520	774	532	Origin recognition complex subunit 1
112238at33090	Complete duplicated	PGJG003920	774.2	532	Origin recognition complex subunit 1
112325at33090	Complete single-copy	PGJG394510	375.3	295	sister chromatid cohesion protein DCC1
112461at33090	Complete single-copy	PGJG232880	645.4	368	FAD-binding, type 2
112542at33090	Complete duplicated	PGJG161180	443.7	246	cytochrome c-type biogenesis ccda-like chloroplastic protein
112542at33090	Complete duplicated	PGJG246580	456.2	280	cytochrome c-type biogenesis ccda-like chloroplastic protein
113024at33090	Complete single-copy	PGJG359810	370.4	245	Queuosine salvage protein
114996at33090	Complete single-copy	PGJG291920	1283.7	938	Elongator complex protein 1
115240at33090	Complete single-copy	PGJG070960	470.4	315	Ubiquitin domain
115992at33090	Complete duplicated	PGJG295790	435.1	254	WD40-repeat-containing domain
115992at33090	Complete duplicated	PGJG033760	409.1	254	WD40-repeat-containing domain

115992at33090	Complete duplicated	PGJG132200	409.1	254	WD40-repeat-containing domain
116411at33090	Complete single-copy	PGJG381610	375.8	222	WD40/YVTN repeat-like-containing domain superfamily
116516at33090	Complete single-copy	PGJG294130	602.3	354	preprotein translocase subunit SCY1, chloroplastic
116585at33090	Complete single-copy	PGJG235700	460.4	294	peroxisome biogenesis factor 10
116623at33090	Complete single-copy	PGJG239360	389.9	276	zinc finger HIT domain-containing protein 2
117191at33090	Complete single-copy	PGJG123020	415.3	280	NADH dehydrogenase
117247at33090	Complete single-copy	PGJG010020	471.6	271	Protein phosphatase methylesterase 1
119439at33090	Complete duplicated	PGJG183680	591	396	Arginine biosynthesis bifunctional protein ArgJ, chloroplastic
119439at33090	Complete duplicated	PGJG336410	516	337	Arginine biosynthesis bifunctional protein ArgJ, chloroplastic
120031at33090	Complete single-copy	PGJG226710	377.9	297	FAD dependent oxidoreductase
120338at33090	Complete single-copy	PGJG268750	658.8	363	Methylthiotransferase, N-terminal
120455at33090	Fragmented	PGJG243140	308	154	Uracil-DNA glycosylase
120589at33090	Complete single-copy	PGJG179240	670.7	427	beta-catenin-like protein 1
121281at33090	Complete single-copy	PGJG388620	414.8	276	Formyl transferase, C-terminal
121303at33090	Complete single-copy	PGJG320100	585.5	361	Alpha/Beta hydrolase fold
122367at33090	Complete single-copy	PGJG319430	490	335	Amine oxidase
122422at33090	Complete single-copy	PGJG072810	395.8	246	dehydrogenase/reductase SDR family member 12
122831at33090	Complete single-copy	PGJG371910	458.7	325	DEAD-box ATP-dependent RNA helicase 22
122958at33090	Complete single-copy	PGJG145830	423.1	261	Leucine carboxyl methyltransferase 1 homolog
123838at33090	Complete single-copy	PGJG009920	356.6	229	alpha-ketoglutarate-dependent dioxygenase alkB
125550at33090	Complete single-copy	PGJG302830	170.1	135	Regulatory protein recX
125747at33090	Complete single-copy	PGJG258670	260.4	205	predicted protein
126138at33090	Complete single-copy	PGJG357240	626.1	439	DnaJ domain
126427at33090	Complete duplicated	PGJG336390	386.9	264	Methionine adenosyltransferase 2 subunit beta
126427at33090	Complete duplicated	PGJG183710	401.7	264	Methionine adenosyltransferase 2 subunit beta
127059at33090	Complete single-copy	PGJG122840	601.8	320	Tryptophan-tRNA ligase
128774at33090	Complete single-copy	PGJG262780	298.5	222	Proteasome assembly chaperone 2
130268at33090	Complete duplicated	PGJG265070	407.9	258	putative methyltransferase At1g22800
130268at33090	Complete duplicated	PGJG283690	362.2	215	putative methyltransferase At1g22800
130279at33090	Complete single-copy	PGJG167400	382.5	254	Putative plastid-lipid-associated protein 12, chloroplastic
130637at33090	Complete single-copy	PGJG397430	471.5	256	cyclin-dependent kinase E-1
131556at33090	Complete single-copy	PGJG096670	504.1	303	SUF system FeS cluster assembly, SufBD
131874at33090	Complete single-copy	PGJG277810	884.7	522	ABC transporter B family member 28
131914at33090	Complete single-copy	PGJG315590	295.2	218	Aspartate/glutamate/uridylate kinase
133112at33090	Complete single-copy	PGJG344080	334.4	252	predicted protein
133622at33090	Fragmented	PGJG315400	294.5	154	OTU domain-containing protein 3 isoform X1
134078at33090	Complete single-copy	PGJG332790	472.2	270	Pseudouridine-5'-phosphate glycosidase
135419at33090	Complete single-copy	PGJG360820	498.7	283	NAD(P)-binding domain
135892at33090	Complete single-copy	PGJG302490	695.5	368	sphingosine-1-phosphate lyase
136118at33090	Complete single-copy	PGJG210440	799.6	480	WD40-repeat-containing domain
136267at33090	Complete single-copy	PGJG069720	313.9	249	Leucine-rich repeat, cysteine-containing subtype
136579at33090	Complete single-copy	PGJG233960	394.4	317	HI0933-like insert domain superfamily
137018at33090	Complete single-copy	PGJG250450	547.3	286	photosystem II stability/assembly factor HCF136, chloroplastic
137771at33090	Complete single-copy	PGJG075630	924.9	572	kelch domain-containing protein 4
137787at33090	Complete single-copy	PGJG239410	583.5	475	U3 small nucleolar RNA-associated protein 6
138499at33090	Complete single-copy	PGJG323000	228.2	159	Tetratricopeptide repeat
138782at33090	Fragmented	PGJG037770	237	143	predicted protein
139062at33090	Complete single-copy	PGJG121430	368.9	260	SWR1 complex subunit 2
139450at33090	Complete single-copy	PGJG075010	330.9	236	Leucine-rich repeat
139497at33090	Complete single-copy	PGJG336300	1073.3	882	structural maintenance of chromosomes protein 5
140683at33090	Complete duplicated	PGJG140790	207.3	184	Glycerol kinase
140683at33090	Complete duplicated	PGJG052980	207.1	184	Glycerol kinase
140876at33090	Complete single-copy	PGJG210880	363.1	223	psbP domain-containing protein 5, chloroplastic
141370at33090	Complete single-copy	PGJG256960	363.8	211	Integral membrane protein TerC
141861at33090	Complete single-copy	PGJG004730	214.4	146	Alpha-ketoglutarate-dependent dioxygenase AlkB-like
142077at33090	Complete duplicated	PGJG155250	214.4	204	Chlorophyll a-b binding protein, chloroplastic
142077at33090	Complete duplicated	PGJG358170	215.7	212	Chlorophyll a-b binding protein, chloroplastic
142077at33090	Complete duplicated	PGJG358150	215.7	210	Chlorophyll a-b binding protein, chloroplastic

142382at33090	Complete single-copy	PGJG180650	241.1	158	PDZ domain
142433at33090	Complete single-copy	PGJG317300	286.7	224	tRNA/rRNA methyltransferase, SpoU
143102at33090	Complete single-copy	PGJG081090	511.9	289	WAT1-related protein At3g02690, chloroplastic
143347at33090	Complete single-copy	PGJG123680	365.4	227	Tetrapyrrole methylase
143963at33090	Missing				
143978at33090	Complete single-copy	PGJG319260	1203.8	852	protein CTR9 homolog
144144at33090	Fragmented	PGJG009320	143.6	84	CAP Gly-rich domain
144680at33090	Complete single-copy	PGJG374680	313.6	252	Ankyrin repeat-containing domain
144716at33090	Complete single-copy	PGJG313680	347.3	217	CAAX amino terminal protease
144765at33090	Complete single-copy	PGJG292680	409.6	254	N-glycosylase/DNA lyase OGG1
144990at33090	Complete single-copy	PGJG076620	400.7	252	NADH dehydrogenase (Ubiquinone) complex I, assembly factor 6
145061at33090	Complete single-copy	PGJG188330	789.3	468	protein high chlorophyll fluorescent 107
145734at33090	Complete single-copy	PGJG287350	422	260	alkylated DNA repair protein alkB homolog 8
146354at33090	Complete single-copy	PGJG400090	283.4	190	thioredoxin-like protein CDSP32, chloroplastic
146469at33090	Complete single-copy	PGJG401850	340.3	211	delta(3,5)-Delta(2,4)-dienoyl-CoA isomerase, peroxisomal
146772at33090	Complete single-copy	PGJG260300	393.3	246	Pantoate-beta-alanine ligase
146921at33090	Complete single-copy	PGJG004100	362.4	210	magnesium protoporphyrin IX methyltransferase, chloroplastic
147083at33090	Complete single-copy	PGJG091680	303.7	242	NAD(P)-binding domain superfamily
147262at33090	Complete single-copy	PGJG142220	391.7	242	protein ABCI12, chloroplastic
148236at33090	Complete single-copy	PGJG030190	236.6	187	Uncharacterised protein family UPF0454
148539at33090	Complete single-copy	PGJG311820	568.7	373	tRNA modification GTPase MnmE
148839at33090	Complete single-copy	PGJG043250	478.4	324	enhanced ethylene response protein 5
149782at33090	Complete single-copy	PGJG191380	240.1	173	Ribosome recycling factor
150470at33090	Complete single-copy	PGJG346250	333.9	251	protein HGH1 homolog
150559at33090	Missing				
152317at33090	Complete single-copy	PGJG325740	369	237	predicted protein
153820at33090	Complete single-copy	PGJG147810	506.5	301	uncharacterized protein YNL011C
154870at33090	Fragmented	PGJG243690	374.2	265	nicastrin
155102at33090	Complete duplicated	PGJG320620	302.8	164	cytochrome c oxidase assembly protein COX11, mitochondrial
155102at33090	Complete duplicated	PGJG264220	302.8	166	cytochrome c oxidase assembly protein COX11, mitochondrial
156843at33090	Complete single-copy	PGJG278640	294.6	183	Ribosomal protein L3
156887at33090	Fragmented	PGJG114150	206.1	118	ABC transporter, conserved site
158492at33090	Complete single-copy	PGJG264730	328.9	199	Ribosome biogenesis protein
158957at33090	Complete single-copy	PGJG284520	196.4	131	HAD superfamily
160064at33090	Complete single-copy	PGJG076540	343.5	208	Electron transfer flavoprotein, beta subunit
160610at33090	Complete single-copy	PGJG357720	426.9	243	RNA-binding S4 domain
160680at33090	Complete single-copy	PGJG288610	821.7	552	cleavage stimulation factor subunit 77
161054at33090	Complete single-copy	PGJG047230	350.2	253	Release factor glutamine methyltransferase
161066at33090	Complete single-copy	PGJG380210	393.9	289	putative oxidoreductase TDA3
161269at33090	Complete single-copy	PGJG018150	332.7	160	deoxycytidylate deaminase
161309at33090	Complete single-copy	PGJG161310	335.7	265	Calycin
161559at33090	Complete single-copy	PGJG307320	323.5	229	protein farnesyltransferase/geranylgeranyltransferase type-1 subunit alpha
161966at33090	Complete single-copy	PGJG325340	282.2	206	RNA-binding S4 domain
162794at33090	Complete single-copy	PGJG072220	480.9	246	Putative tRNA (cytidine(32)/guanosine(34)-2'-O)-methyltransferase
163097at33090	Complete single-copy	PGJG125380	542.5	305	peptidyl-prolyl cis-trans isomerase CYP38, chloroplastic
163701at33090	Complete single-copy	PGJG049010	200.1	149	putative uridine kinase C227.14
163833at33090	Complete single-copy	PGJG003550	627.4	360	tRNA threonylcarbamoyladenosine dehydratase
164019at33090	Fragmented	PGJG403870	176.3	109	Class IV aminotransferase
164280at33090	Complete single-copy	PGJG304080	272.4	144	pre-mRNA-splicing factor 38
164556at33090	Complete single-copy	PGJG289780	195	148	Putative rRNA methylase
165103at33090	Complete single-copy	PGJG243390	396.6	235	Carbon-nitrogen hydrolase
166645at33090	Complete single-copy	PGJG174740	1189.7	942	Sister chromatid cohesion protein
166660at33090	Complete single-copy	PGJG258800	234	143	ATP-dependent helicase/deoxyribonuclease subunit B
167076at33090	Complete single-copy	PGJG022730	226.4	158	oxidoreductase activity
167217at33090	Complete single-copy	PGJG357500	424.1	216	tRNA (guanine-N(7)-)-methyltransferase
167325at33090	Complete single-copy	PGJG288270	184	176	chromosome-associated kinesin

167809at33090	Complete single-copy	PGJG323390	336.5	165	Peptidyl-prolyl cis-trans isomerase
167850at33090	Fragmented	PGJG325330	165.5	111	ubiquitin-like-conjugating enzyme ATG10
167932at33090	Complete single-copy	PGJG282280	365.4	191	psbP domain-containing protein 4, chloroplastic
168269at33090	Missing				
168270at33090	Complete single-copy	PGJG337980	435	272	protein TAB2 homolog, chloroplastic
168563at33090	Complete single-copy	PGJG130150	172.4	134	Tetratricopeptide repeat
169007at33090	Complete single-copy	PGJG113410	259.8	144	tRNA (guanine-N1-)-methyltransferase, N-terminal
170939at33090	Complete single-copy	PGJG120970	612.9	338	ATPase, AAA-type, conserved site
170945at33090	Complete single-copy	PGJG345860	516	342	DEAD-box ATP-dependent RNA helicase 47, mitochondrial
171195at33090	Complete single-copy	PGJG022760	310.7	193	urease accessory protein F
171627at33090	Complete duplicated	PGJG320610	187.3	137	protein OPI10 homolog
171627at33090	Complete duplicated	PGJG264210	186.3	137	protein OPI10 homolog
171708at33090	Complete single-copy	PGJG358670	286.6	241	protein SEH1
171767at33090	Complete single-copy	PGJG294950	303.8	178	psbP domain-containing protein 6, chloroplastic
172000at33090	Complete duplicated	PGJG279640	461.8	288	ribosomal RNA small subunit methyltransferase H
172000at33090	Complete duplicated	PGJG279660	440.4	282	ribosomal RNA small subunit methyltransferase H
172535at33090	Complete single-copy	PGJG173830	654.3	398	Ubiquinone biosynthesis monooxygenase COQ6, mitochondrial
172666at33090	Fragmented	PGJG118360	209.6	114	Peptide deformylase
173119at33090	Complete single-copy	PGJG269870	249.6	188	DNA repair protein XRCC3 homolog
173143at33090	Complete single-copy	PGJG169510	241.2	155	Nucleoporin, NSP1-like, C-terminal
173477at33090	Complete single-copy	PGJG061250	192	133	PITH domain
173581at33090	Complete single-copy	PGJG020490	422.6	249	putative deoxyribonuclease TATDN1
174202at33090	Complete single-copy	PGJG096680	182.8	138	putative RNA methyltransferase At5g10620
174213at33090	Fragmented	PGJG007510	132.9	86	protein FRA10AC1
174767at33090	Complete single-copy	PGJG342220	451.2	236	Histidine phosphatase superfamily
175583at33090	Complete single-copy	PGJG301830	182.2	131	probable plastid-lipid-associated protein 11
175792at33090	Complete single-copy	PGJG136550	268.3	184	homologous-pairing protein 2 homolog
175963at33090	Complete duplicated	PGJG167900	350.4	190	Putative plastid-lipid-associated protein 6, chloroplastic
175963at33090	Complete duplicated	PGJG047430	366.5	206	Putative plastid-lipid-associated protein 6, chloroplastic
176016at33090	Complete single-copy	PGJG342370	303.2	157	N-terminal acetyltransferase B complex catalytic subunit NAA20
176328at33090	Complete single-copy	PGJG145160	688.9	454	eukaryotic translation initiation factor 2D
177296at33090	Complete single-copy	PGJG344410	278.4	163	methyltransferase-like protein 5
177720at33090	Complete single-copy	PGJG153680	441.6	379	peptide-N(4)-(N-acetyl-beta- glucosaminyl)asparagine amidase
177964at33090	Complete single-copy	PGJG227940	320.2	195	peptidyl-prolyl cis-trans isomerase FKBP19, chloroplastic
178386at33090	Complete single-copy	PGJG355470	366.5	229	Glucose-6-phosphate 1-epimerase
179975at33090	Fragmented	PGJG286490	193.2	103	Thylakoid lumenal protein
180408at33090	Complete single-copy	PGJG221350	278.2	168	protein COFACTOR ASSEMBLY OF COMPLEX C SUBUNIT B CCB1, chloroplastic
180466at33090	Complete single-copy	PGJG198320	330.5	203	protein SCO1 homolog 1, mitochondrial
180674at33090	Complete single-copy	PGJG336680	423.9	235	predicted protein
181108at33090	Complete single-copy	PGJG361660	286.9	170	probable plastid-lipid-associated protein 8, chloroplastic
182493at33090	Complete single-copy	PGJG012460	219.8	170	Peptidyl-prolyl cis-trans isomerase
182513at33090	Complete duplicated	PGJG068080	479.6	283	Protoheme IX farnesyltransferase
182513at33090	Complete duplicated	PGJG174010	467.9	283	Protoheme IX farnesyltransferase
182936at33090	Fragmented	PGJG279480	233.8	164	protein EI24 homolog
182952at33090	Complete single-copy	PGJG404110	273.2	198	Transcriptional regulator TACO1-like
183313at33090	Fragmented	PGJG284170	264.3	147	Protein-ribulosamine 3-kinase, chloroplastic
183396at33090	Missing				
183719at33090	Fragmented	PGJG056410	288.8	182	Tocopherol cyclase
183931at33090	Complete single-copy	PGJG053640	366.9	239	THO complex subunit 3
184066at33090	Complete single-copy	PGJG358640	167.7	95	1-acyl-sn-glycerol-3-phosphate acyltransferase
184405at33090	Complete single-copy	PGJG052850	454.9	324	adenine DNA glycosylase
184776at33090	Complete single-copy	PGJG101240	257.5	158	nifU-like protein 1, chloroplastic
185172at33090	Complete single-copy	PGJG372090	175.3	111	Molybdopterin synthase catalytic subunit
185214at33090	Missing				
185696at33090	Complete single-copy	PGJG028230	671.5	464	transcription factor 25
185941at33090	Fragmented	PGJG097910	147.7	97	psbP domain-containing protein 3, chloroplastic

186340at33090	Complete single-copy	PGJG202680	336.9	214	psbP domain-containing protein 1, chloroplastic
187462at33090	Complete single-copy	PGJG315820	521.4	382	Cyclophilin-type peptidyl-prolyl cis-trans isomerase
187866at33090	Complete duplicated	PGJG057960	203.2	158	DTW domain-containing protein 2
187866at33090	Complete duplicated	PGJG156550	201	165	DTW domain-containing protein 2
187914at33090	Complete single-copy	PGJG211500	480.5	356	Folylpolyglutamate synthetase
188180at33090	Complete single-copy	PGJG384040	282.4	144	Lactoylglutathione lyase
188436at33090	Complete single-copy	PGJG218460	253	175	protein Mpv17
188583at33090	Complete single-copy	PGJG154620	421.2	261	DNA excision repair protein ERCC-1
188938at33090	Complete single-copy	PGJG339170	337.7	182	Protein of unknown function DUF2854
189891at33090	Complete single-copy	PGJG105220	362.5	209	Tetrapyrrole methylase
190390at33090	Complete single-copy	PGJG106720	290	195	Peroxidase
191125at33090	Complete single-copy	PGJG100570	387.1	249	Pseudouridine synthase
191396at33090	Complete single-copy	PGJG088060	415.8	255	tRNA (cytosine(38)-C(5))-methyltransferase isoform X1
191581at33090	Complete duplicated	PGJG391010	401	224	UPF0603 protein At1g54780, chloroplastic
191581at33090	Complete duplicated	PGJG015550	399.5	224	UPF0603 protein At1g54780, chloroplastic
192090at33090	Complete single-copy	PGJG305990	619.2	458	MIF4G-like domain superfamily
192376at33090	Complete single-copy	PGJG274270	179.8	105	putative peptidyl-tRNA hydrolase PTRHD1
192456at33090	Complete single-copy	PGJG023000	135.6	154	OTU domain
192750at33090	Complete single-copy	PGJG122570	255.7	184	ATP-dependent Clp protease proteolytic subunit
193351at33090	Complete single-copy	PGJG079880	244.1	133	DCC family protein At1g52590, chloroplastic
193437at33090	Complete single-copy	PGJG311430	211.7	131	Molybdopterin cofactor biosynthesis C (MoaC) domain
193683at33090	Complete single-copy	PGJG379510	197.8	131	multiple myeloma tumor-associated protein 2 homolog
193777at33090	Complete single-copy	PGJG398080	420.3	250	Peptidase M41-like
193982at33090	Fragmented	PGJG096710	113.4	119	Ubiquitin domain
194002at33090	Complete single-copy	PGJG377250	248.2	143	Impact, N-terminal
194293at33090	Complete single-copy	PGJG284190	289.7	225	rRNA adenine N(6)-methyltransferase
194372at33090	Complete single-copy	PGJG034440	189	101	uncharacterized protein ycf20
194467at33090	Complete single-copy	PGJG326660	325.4	180	Protein of unknown function DUF4079
195185at33090	Complete single-copy	PGJG135940	364.2	208	50S ribosomal protein L4, chloroplastic
195354at33090	Fragmented	PGJG194380	160.7	95	DNA binding protein
195527at33090	Complete single-copy	PGJG210210	463.9	321	tRNA pseudouridine synthase
195843at33090	Complete single-copy	PGJG132610	485.6	322	Meiotic nuclear division protein 1 homolog
196198at33090	Complete single-copy	PGJG370630	146.1	94	thioredoxin-like protein CITRX, chloroplastic
196248at33090	Complete single-copy	PGJG186260	181.2	122	Transcription termination factor, mitochondrial/chloroplastic
196324at33090	Fragmented	PGJG335550	186.1	135	Alpha/Beta hydrolase fold
196325at33090	Complete single-copy	PGJG395750	285.5	240	WD40-repeat-containing domain
196410at33090	Complete single-copy	PGJG311670	272.3	166	alpha N-terminal protein methyltransferase 1
196443at33090	Complete single-copy	PGJG344910	121	97	thylakoid lumenal 15 kDa protein 1, chloroplastic
196490at33090	Complete single-copy	PGJG294070	300.1	213	YaeB-like, N-terminal domain
196512at33090	Complete single-copy	PGJG314640	595.6	358	Ribosome biogenesis GTPase RsgA
196850at33090	Complete single-copy	PGJG177370	219.8	130	Ribosome-binding factor A
196873at33090	Complete single-copy	PGJG221330	295.2	149	N-alpha-acetyltransferase MAK3
196991at33090	Fragmented	PGJG317920	36.1	51	G patch domain-containing protein 11
197780at33090	Complete single-copy	PGJG377590	339.2	182	tRNA (Guanosine(18)-2'-O)-methyltransferase
197838at33090	Complete single-copy	PGJG187520	232.4	134	Tonoplast intrinsic protein, alpha (Alpha-TIP)
198012at33090	Complete single-copy	PGJG275130	189.3	122	D-aminoacyl-tRNA deacylase
198570at33090	Complete single-copy	PGJG270570	454.5	274	F-box protein 7
199163at33090	Complete single-copy	PGJG046840	383.2	258	Nucleic acid-binding, OB-fold
199618at33090	Complete single-copy	PGJG034690	474.9	325	tRNA (guanine(26)-N(2))-dimethyltransferase
199795at33090	Complete single-copy	PGJG160190	252.3	133	thylakoid lumenal 15.0 kDa protein 2, chloroplastic
200316at33090	Complete single-copy	PGJG312620	414.3	294	Cyclophilin-type peptidyl-prolyl cis-trans isomerase
200488at33090	Complete single-copy	PGJG316940	345.4	259	DNA polymerase I
200499at33090	Complete single-copy	PGJG351210	523.8	323	PPM-type phosphatase domain
200687at33090	Complete single-copy	PGJG016630	340.6	181	thylakoid lumenal 17.4 kDa protein, chloroplastic
201861at33090	Complete single-copy	PGJG140520	336.9	244	Ubiquinol-cytochrome c chaperone, CBP3
202025at33090	Complete single-copy	PGJG380420	230.8	153	predicted protein
203023at33090	Complete single-copy	PGJG133010	234.3	146	Rubredoxin
203383at33090	Complete single-copy	PGJG269010	359.1	246	protein RTF2 homolog
203818at33090	Complete single-copy	PGJG031000	169	118	nucleolar protein 16

204117at33090	Complete single-copy	PGJG113140	233.9	169	predicted protein
204880at33090	Complete single-copy	PGJG333390	369.5	211	Protein of unknown function DUF2301
205308at33090	Complete single-copy	PGJG359280	278.1	172	RNA exonuclease 4
206164at33090	Complete single-copy	PGJG360350	258.9	142	PUA domain
206170at33090	Complete single-copy	PGJG160370	262.8	156	uncharacterized protein LOC103413686
207179at33090	Complete single-copy	PGJG340590	491.5	306	Peroxisome biogenesis protein 12
207898at33090	Complete single-copy	PGJG302330	233.6	158	Ribosomal protein L9
208706at33090	Complete single-copy	PGJG342450	182.8	137	ribosome-recycling factor, chloroplastic
208707at33090	Complete single-copy	PGJG285330	70.3	53	predicted protein
208849at33090	Complete single-copy	PGJG129280	113.3	70	LSM domain, eukaryotic/archaea-type
209015at33090	Fragmented	PGJG390020	292.7	130	Nuclear cap-binding protein subunit 2
209301at33090	Complete duplicated	PGJG204450	376.9	267	tetratricopeptide repeat protein 4 homolog
209301at33090	Complete duplicated	PGJG169050	337.3	247	tetratricopeptide repeat protein 4 homolog
209302at33090	Complete single-copy	PGJG169450	323.6	186	probable mitochondrial import inner membrane translocase subunit TIM21
209516at33090	Complete single-copy	PGJG052300	324.9	166	peptidyl-prolyl cis-trans isomerase FKBP20-2, chloroplastic
210493at33090	Complete single-copy	PGJG074570	186.6	130	protein canopy-1
210551at33090	Complete duplicated	PGJG020030	319.1	183	thioredoxin-like protein HCF164, chloroplastic
210551at33090	Complete duplicated	PGJG285940	320.2	183	thioredoxin-like protein HCF164, chloroplastic
210557at33090	Complete single-copy	PGJG315310	164.4	127	Protein of unknown function DUF3054
210851at33090	Complete single-copy	PGJG235320	255.9	128	Glyoxalase/Bleomycin resistance protein/Dihydroxybiphenyl dioxygenase
211645at33090	Complete single-copy	PGJG232470	363.7	214	Ribosomal RNA small subunit methyltransferase G
212331at33090	Complete single-copy	PGJG332730	474.6	366	nuclear pore complex protein NUP54
212845at33090	Complete single-copy	PGJG030400	249.4	169	psbP domain-containing protein 7, chloroplastic
213827at33090	Missing				
213828at33090	Complete single-copy	PGJG362810	217.2	124	NAD(P)-linked oxidoreductase superfamily protein
214313at33090	Complete single-copy	PGJG001900	107.4	120	UPF0690 protein C1orf52 homolog
215062at33090	Complete single-copy	PGJG186390	219	142	N-acetyltransferase 9-like protein
215487at33090	Complete single-copy	PGJG288020	103.1	91	recQ-mediated genome instability protein 2
215817at33090	Missing				
216228at33090	Complete single-copy	PGJG243610	265.5	176	Protein of unknown function DUF1997
216601at33090	Complete single-copy	PGJG200980	204.4	145	Pyridoxamine 5'-phosphate oxidase, Alr4036 family, FMN-binding domain
217014at33090	Complete single-copy	PGJG327300	282.1	198	protein COFACTOR ASSEMBLY OF COMPLEX C SUBUNIT B CCB4, chloroplastic
217521at33090	Complete single-copy	PGJG108490	169.4	131	cardiolipin synthase (CMP-forming), mitochondrial
218947at33090	Complete single-copy	PGJG385660	176.2	92	predicted protein
219128at33090	Complete single-copy	PGJG108390	124.3	72	predicted protein
219839at33090	Complete single-copy	PGJG147220	90.4	122	Ribosomal protein L18
220165at33090	Complete single-copy	PGJG193550	267.2	180	Dimethylallyl, adenosine tRNA methylthiotransferase
220316at33090	Complete single-copy	PGJG097360	176.3	131	Co-chaperone Hsc20
220587at33090	Complete single-copy	PGJG127810	299.9	204	protein OS-9 homolog
220589at33090	Fragmented	PGJG220560	230.1	185	Rubisco LSMT, substrate-binding domain
220981at33090	Complete single-copy	PGJG402450	257.7	195	26S proteasome non-ATPase regulatory subunit 9
223902at33090	Complete single-copy	PGJG038010	254.2	152	protein disulfide-isomerase 5-1
225336at33090	Complete single-copy	PGJG137300	200.7	140	predicted protein
227848at33090	Complete single-copy	PGJG062740	274.2	190	RWD domain-containing protein 1
228201at33090	Complete single-copy	PGJG203530	349.8	249	predicted protein
229764at33090	Complete duplicated	PGJG158630	460.5	290	probable inactive shikimate kinase like 2, chloroplastic
229764at33090	Complete duplicated	PGJG361910	465.7	294	probable inactive shikimate kinase like 2, chloroplastic
229778at33090	Complete single-copy	PGJG122380	148.5	105	predicted protein
229782at33090	Complete single-copy	PGJG398900	120.4	69	UPF0161 protein At3g09310
230103at33090	Complete single-copy	PGJG254550	489.2	309	28S ribosomal protein S29, mitochondrial
231151at33090	Fragmented	PGJG330130	151.7	109	NAD(P)-binding domain
231707at33090	Complete single-copy	PGJG298050	350.3	213	spindle and kinetochore-associated protein 1 homolog
233744at33090	Complete duplicated	PGJG229310	170	118	protein Iojap-related, mitochondrial
233744at33090	Complete duplicated	PGJG236620	184	116	protein Iojap-related, mitochondrial
234046at33090	Complete single-copy	PGJG137940	168.4	118	prefoldin subunit 1
238204at33090	Missing				

238385at33090	Complete duplicated	PGJG232580	117.6	70	photosystem I reaction center subunit N
238385at33090	Complete duplicated	PGJG232570	117.6	70	photosystem I reaction center subunit N

Supplementary Table S20. Summary of re-mapped short reads to the draft genome assembly of *P. grandiflorus*

Type	Insert size of library	No. of clean reads	No. of mapped reads	(%)	No. mapped paired-end reads	(%)
Short-insert library	270 bp	125,871,360	124,384,746	98.82%	117,562,020	93.40%
		109,696,374	108,367,869	98.79%	102,179,372	93.15%
	500 bp	116,091,356	114,166,425	98.34%	102,890,296	88.63%
		134,998,200	133,094,466	98.59%	119,778,170	88.73%
	700 bp	107,117,594	104,687,225	97.73%	92,499,848	86.35%
		135,409,670	131,331,834	96.99%	114,007,218	84.19%
	360 bp	102,390,348	100,499,812	98.15%	92,771,166	90.61%
Long-mate pairs library	2 kb	24,584,288	23,807,590	96.84%	19,316,106	78.57%
		23,480,720	22,731,573	96.81%	18,575,202	79.11%
		24,040,790	23,328,899	97.04%	19,079,642	79.36%
	5 kb	25,962,898	25,123,711	96.77%	18,170,174	69.99%
		25,698,948	24,895,332	96.87%	18,108,366	70.46%
		22,586,920	21,787,292	96.46%	15,273,388	67.62%
	10 kb	11,534,744	11,031,192	95.63%	6,513,560	56.47%
		7,985,868	7,662,507	95.95%	4,373,272	54.76%
		12,510,444	12,053,345	96.35%	6,911,864	55.25%
